# Injured Myocardium‐Targeted Theranostic Nanoplatform for Multi‐Dimensional Immune‐Inflammation Regulation in Acute Myocardial Infarction

**DOI:** 10.1002/advs.202414740

**Published:** 2025-01-21

**Authors:** Tao Zheng, Jie Sheng, Zhiyue Wang, Haoguang Wu, Linlin Zhang, Sheng Wang, Jianhua Li, Yunming Zhang, Guangming Lu, Longjiang Zhang

**Affiliations:** ^1^ Department of Radiology, Nanjing Jinling Hospital, Affiliated Hospital of Medical School Nanjing University 305 East Zhongshan Road Nanjing 210002 China; ^2^ Department of Radiology, Nanjing Jinling Hospital Nanjing Medical University 305 East Zhongshan Road Nanjing 210002 China; ^3^ Department of Cardiology, Nanjing Jinling Hospital, Affiliated Hospital of Medical School Nanjing University 305 East Zhongshan Road Nanjing 210002 China

**Keywords:** immune response, inflammation, myocardial infarction, nanoplatform, pyroptosis

## Abstract

Pyroptosis is a key mode of programmed cell death during the early stages following acute myocardial infarction (AMI), driving immune‐inflammatory responses. Cardiac resident macrophages (CRMs) are the primary mediators of cardiac immunity, and they serve a dual role through their shaping of both myocardial injury and post‐AMI myocardial repair. To appropriately regulate AMI‐associated inflammation, HM4oRL is herein designed, an innovative bifunctional therapeutic nanoplatform capable of inhibiting cardiomyocyte pyroptosis while reprogramming inflammatory signaling. This HM4oRL platform is composed of a core of 4‐Octyl itaconate (4‐OI)‐loaded liposomes, a middle layer consisting of a metal‐polyphenol network (MPN) film, and an optimized outer hybrid immune‐cell membrane layer. The unique properties of this hybrid membrane layer facilitated HM4oRL targeting to the injured myocardium during early‐stage AMI in mice, whereupon the release of 4‐Ol and modified MPN synergistically inhibited cardiomyocyte pyroptosis while suppressing inflammatory monocytes/macrophage responses at the infarcted site. Mechanistically, HM4oRL preserved cardiac metabolic homeostasis through AMPK signaling activation, establishing favorable microenvironmental conditions for the reprogramming of CRM‐mediated inflammation. Ultimately, HM4oRL treatment is able to resolve inflammation, enhance neovascularization, and suppress myocardial fibrosis, reducing the infarct size and enhancing post‐AMI cardiac repair such that it is an innovative approach to the targeted treatment of AMI.

## Graphical abstract

The multifunctional biomimetic HM4oRL nanoparticles offer a dual function of regulating immune‐inflammation and enabling T1‐weighted MR imaging in acute myocardial infarction (AMI). By suppressing ischemia‐induced cardiomyocyte pyroptosis, preserving cardiac metabolic homeostasis, and reprogramming cardiac monocyte/macrophage‐mediated inflammatory responses, this nanoplatform alleviates acute myocardial injury and promotes post‐AMI cardiac repair.

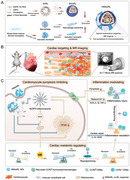

## 1 Introduction

Cardiac ischemia results in the injury and death of cardiomyocytes, eliciting a robust inflammatory response that profoundly shapes subsequent myocardial damage, wound healing, and reparative processes.^[^ ^1^ ^]^ Percutaneous coronary intervention and other forms of reperfusion therapy have significantly lower rates of overall mortality among AMI patients.^[^ ^2^ ^]^ Despite these advances, 12–15% of first‐episode AMI patients nonetheless progress to heart failure (HF) as a consequence of inadequate tissue repair and adverse cardiac remodeling.^[^ ^3^ ^]^ There is thus a pressing need to develop novel treatment strategies capable of both abrogating acute myocardial injury while also optimizing the process of infarct healing to minimize the risk of post‐AMI progression to HF.

In mouse model studies, pyroptosis has recently been shown to play a key role in the pathologic progression that occurs within the first 3 days after MI.^[^ ^4^ ^]^ Pyroptosis is a form of pyrin domain‐containing protein 3 (NLRP3)/Caspase‐1 inflammasome‐ and gasdermin D (GSDMD)‐mediated inflammatory cell death.^[^ ^5^ ^]^ It is also the predominant form of programmed cell death (PCD) observed in AMI lesions such that cardiomyocyte pyroptosis is thought to contribute to both infarct enlargement and declining cardiac function.^[^ ^6^ ^]^ When cardiomyocytes undergo pyroptotic death, the inflammatory mediators that they release, including interleukin‐1β (IL‐1β) can activate monocytes and macrophages to establish a pro‐inflammatory immune microenvironment.^[^ ^7^ ^]^ The prevention of cardiomyocyte pyroptosis following AMI thus has the potential to modulate the broader injured myocardial immune microenvironment.

Cardiac resident macrophages (CRMs) are the most common immune cells in the heart, and they have also been established as central regulators of the immune responses engaged following cardiac injury.^[^ ^8^ ^]^ Murine CRMs can be separated into two functionally specialized subsets according to their expression of the surface markers C‐C chemokine receptor type 2 (CCR2), C‐X3‐C motif chemokine receptor 1 (CX3CR1), and major histocompatibility complex‐II (MHC‐II). These CRMs exhibit dynamic functional and phenotypic properties that depend on local microenvironmental physiological and pathological conditions.^[^ ^9^ ^]^ Altered metabolic activity in the cardiac tissue, notably, has been shown to be closely tied to cardiac macrophage activity, emphasizing the role that tissue metabolism plays in the orchestration of immune responses.^[^ ^10^ ^]^ Glycolytic intermediates, for instance, can function as metabolic signaling intermediaries that can exacerbate the inflammatory activation of macrophages, whereas reparative macrophage functions instead depend on oxidative mitochondrial metabolism and fatty acid β‐oxidation.^[^ ^11^ ^]^ The persistent presence of pro‐inflammatory cytokines, metabolites, and reactive oxygen species (ROS) can trigger further CCR2^+^ monocyte/macrophage infiltration, culminating in prolonged myocardial inflammation after injury. When tissue repair begins, as occurs ≈3–7 days after AMI in mice, the presence of insufficient CCR2^−^ CRMs with reparative functions can lead to insufficient infarct healing, contributing to adverse remodeling and contractile dysfunction.^[^ ^12^ ^]^ These effects are a consequence of the central roles that these CCR2^−^ CRMs play as mediators of neovascularization, inflammatory resolution, and the alleviation of fibrosis in the infarcted tissue. Comprehensive approaches to alleviating the pyroptotic death of cardiomyocytes while also modulating the cardiac immune‐inflammatory response in a manner conducive to optimal tissue repair would thus hold great promise as a treatment for AMI.

Small molecule inhibitors of pyroptosis have been reported to be capable of hampering post‐AMI inflammation, but efforts to successfully translate these therapeutic agents to the clinic are lacking at present.^[^ ^13^ ^]^ This is likely attributable in part to the complex cardiac microenvironment in AMI patients, together with the poor targeting efficiency and insufficient biocompatibility of extant small molecule inhibitors.^[^ ^4^, ^14^ ^]^ Nanoparticle‐based drug delivery systems (nano‐DDSs) have recently emerged as attractive platforms for targeted therapeutic compound delivery.^[^ ^15^ ^]^ The advantages of these nanoparticle (NP)‐based drug carriers include their amenability to functional modification such that they can be targeted to specific sites, their compatibility with combination treatment strategies, and their ability to facilitate controlled drug release to more effectively manage the diseases they are used to treat. The development of a cardiac‐targeted nanoplatform that can both protect cardiomyocytes and control inflammatory responses would thus offer a high degree of value for the management of cascading post‐AMI inflammatory responses.

**Scheme 1:**
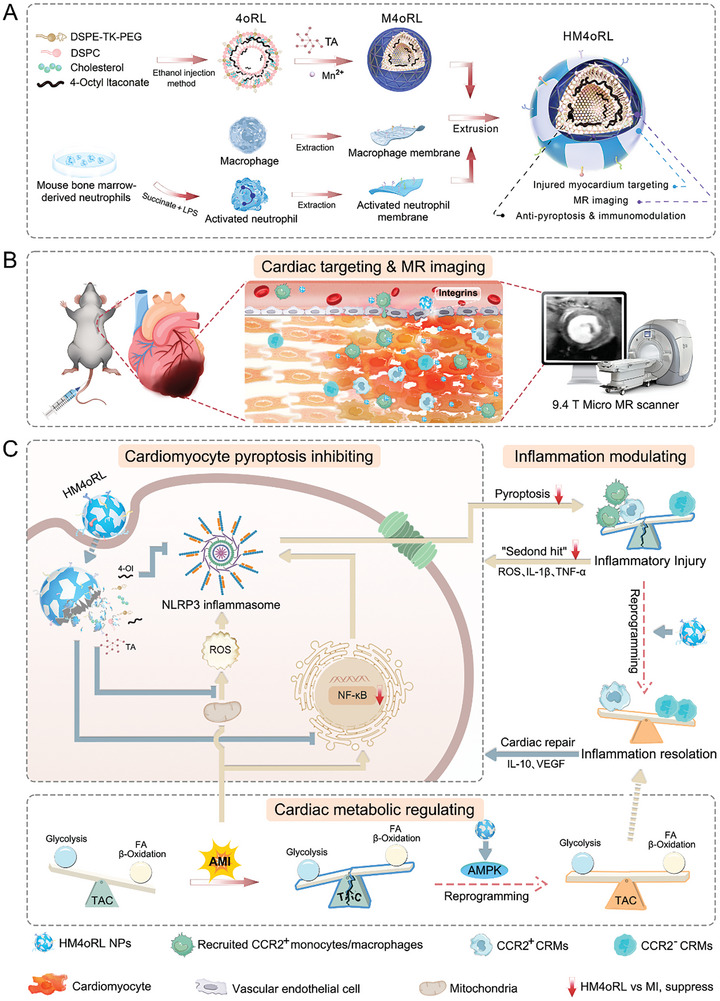
A) Schematic overview of the approach to the preparation of multifunctional biomimetic HM4oRL nanoparticles (NPs). B) Schematic overview of the targeted effects of HM4oRL NPs on the damaged myocardium and as tools T1‐weighted MR imaging with a 9.4 T Micro MR scanner in a murine model of AMI. C) The proposed cardioprotective effects of HM4oRL NPs entail the suppression of ischemia‐induced cardiomyocyte pyroptosis, the preservation of microenvironmental cardiac metabolic homeostasis, and the modulation of cardiac monocyte/macrophage‐mediated inflammatory responses. CRMs: cardiac‐resident macrophages; TAC: tricarboxylic acid cycle.

Itaconate is a tricarboxylic acid cycle (TAC) byproduct with promising utility as a treatment for inflammatory diseases.^[^ ^16^ ^]^ The itaconate derivative 4‐OI can suppress NLRP3 inflammasome activity and regulate glycolysis and fatty acid β‐oxidation.^[^ ^17^ ^]^ Given these properties, 4‐OI may be a valuable agent for therapies seeking to control pyroptotic and metabolic activity in AMI. The poor bioavailability of 4‐Ol, however, remains a persistent barrier to its clinical application. Liposomes are a class of nanocarriers that are widely employed in different nanomedicine platforms owing to their track record of successful clinical utilization and the ease with which they can be functionalized in an application‐specific manner.^[^ ^18^ ^]^ Here, 4‐OI‐loaded liposomes (4‐OI@liposomes, 4oRL) were fabricated in an effort to augment the bioavailability of 4‐Ol. Elevated levels of ROS production in the injured myocardial microenvironment play a key role in the induction and persistence of inflammatory activity. To leverage the robust antioxidant activity of MPN to scavenge these excessive ROS,^[^ ^19^ ^]^ the prepared 4oRL NPs were coated with an MPN film prepared through the self‐assembly of tannic acid (TA) and Mn^2+^, yielding MPN@4‐OI@liposomes (M4oRL). As an added advantage, to good r1 relaxivity of the Mn^2+^ ions used in these M4oRL NPs endowed them with utility in T1‐weighted magnetic resonance (MR) imaging applications. Many studies have sought to improve nanoplatform targeting to injured myocardial tissues given that such targeting would simultaneously improve drug bioavailability while also improving the quality of MR imaging.^[^ ^20^ ^]^ The use of cell‐derived membranes as a biomimetic coating for drug delivery platforms holds great promise, as these membranes can facilitate reliable homing to target lesions.^[^ ^21^ ^]^ Neutrophils are the first immune cells recruited to infarcted sites after AMI, highlighting their intrinsic sensitivity to myocardial damage.^[^ ^22^ ^]^ Strikingly, prior clinical studies have highlighted a direct correlation between the severity of myocardial ischemia and significantly elevated circulating levels of succinate, a pro‐inflammatory metabolite released from the injured myocardium.^[^ ^23^ ^]^ In this study, succinate treatment was used to activate neutrophils in vitro, after which the membranes from these neutrophils were leveraged to sense myocardial damage. Our final platform consisted of a highly novel system of M4oRL encapsulated within a biomimetic hybrid membrane (HM) prepared from a mixture of the membranes of untreated macrophages and activated neutrophils, yielding HM@MPN@4‐OI@liposome (HM4oRL) NPs (**Scheme** **1**).

The unique HM coating of these NPs enabled the successful targeting of these HM4oRL NPs to the injured myocardium, and their utility in MR imaging applications was further confirmed with a 9.4 T Micro MR scanner. In mice, the impact of 4‐OI, 4oRL, M4oRL, and HM4oRL NP treatment on post‐MI infarct size and cardiac function was evaluated, while transcriptomic sequencing was used to systematically explore the mechanistic basis for the cardioprotective benefits of HM4oRL treatment. Together, these analyses revealed that these NPs exert their effects through the regulation of pyroptotic cell death and metabolism, shaping cardiac monocyte/macrophage‐mediated post‐AMI inflammatory responses. These effects highlight the ability of this uniquely, intelligently engineered multifunctional biomimetic nanoplatform to protect cardiomyocytes while restraining immune‐inflammatory responses, providing an opportunity to treat AMI with an unprecedented level of regulatory control.

## 2 Results

### 2.1 HM4oRL NP Synthesis and Characterization

**Figure 1:**
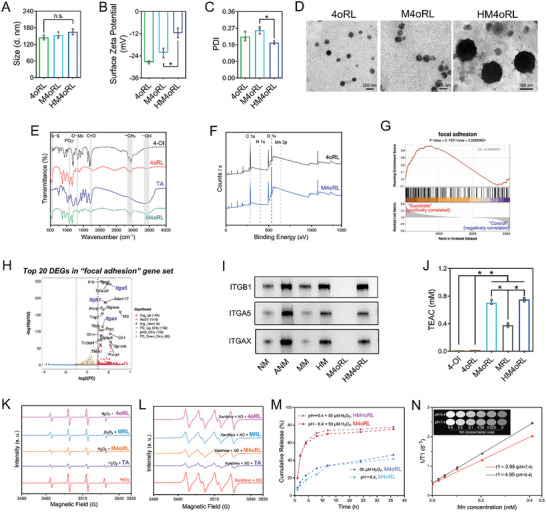
Preparation and characterization of HM4oRL nanoparticles. A) Particle sizes, B) surface Zeta potential, and C) polydispersity index (PDI) of 4oRL, M4oRL, and HM4oRL nanoparticles. n = 3. D) TEM images of 4oRL, M4oRL, and HM4oRL nanoparticles in water, with HM4oRL nanoparticles negatively stained using phosphotungstic acid. E) FTIR spectra of 4‐OI, TA, 4oRL, and M4oRL nanoparticles. F) XPS survey scan spectra for 4oRL and M4oRL nanoparticles. (G) GSEA analysis of “focal adhesion” related enrichment plots derived from transcriptome sequencing in succinate activated neutrophils in vitro. n = 3. H) Top 20 DEGs in the “focal adhesion” gene set. I) Western blots of ITGB1, ITGA5, and ITGAX in neutrophil membrane (NM), activated neutrophil membrane (ANM), macrophage membrane (MM), hybrid membrane (HM), M4oRL, and HM4oRL nanoparticles. ITGB1: Integrin β‐1; ITGA5: Integrin α 5; ITGAX: Integrin α X. J) Antioxidant capacity of 4‐OI, 4oRL, M4oRL, MRL, and HM4oRL nanoparticles measured by ABTS free radical scavenging assays. n = 5. K) ^•^OH and L) ^•^O_2_^−^ scavenging capacity of TA, 4oRL, M4oRL, and MRL nanoparticles by the electron paramagnetic resonance spectra analysis. M) In vitro 4‐OI release profiles from M4oRL and HM4oRL nanoparticles under various conditions. The pH value in the PBS samples was buffered with dilute hydrochloric acid, and the H_2_O_2_ concentration was 50 µM. N) T1‐weighted MR images of various concentrations of HM4oRL nanoparticles in PBS with different pH levels at a 9.4 T Micro MR scanner, and the corresponding regression curves between 1/T1 (s^−1^) and Mn^2+^ concentration (mM). Data are presented as mean ± SD. Statistical methods: One‐way ANOVA with Tukey's post‐test (A‐C), or Tamhane's post‐test (J). In all panels, * indicates *p* < 0.05, and “n. s.” indicates no significance.

A three‐step process was used to synthesize HM4oRL NPs (Scheme 1A). First, an ethanol injection method was used to synthesize 4oRL (4‐OI@liposomes, 4oRL) NPs.^[^ ^24^ ^]^ When 1 mg of 4‐OI and distearoylphosphatidylcholine (DSPC)/cholesterol/distearoyl phosphatidylethanolamine‐thioketal‐polyethylene glycol (DSPE‐TK‐PEG) were mixed at a 0.57:0.38:0.05 molar ratio, the resultant particles exhibited a drug loading efficiency of 10.77 ± 2.59%, were 143.57 ± 7.45 nm in diameter, and had a polydispersity index (PDI) of 0.24 ± 0.01 (Figure S1A, Supporting Information), with a powder yield of ≈76%. In the second step, MPN film‐coated 4oRL (MPN@4‐OI@liposomes, M4oRL) NPs were prepared through TA and Mn^2+^ self‐assembly.^[^ ^25^ ^]^ Higher Mn^2+^ concentrations were associated with increased MPN film thickness for the resultant M4oRL NPs (Figure S1B, Supporting Information). At a TA to MnCl_2_ mass ratio of 1:0.25, M4oRL particles measured 153.17 ± 10.76 nm in size (**Figure** **1**A), with a surface Zeta potential of ‐22.24 ± 2.50 mV (Figure 1B), and a PDI of 0.26 ± 0.02 (Figure 1C). The M4oRL lyophilized powder yield was ≈65%. In the third step, murine bone marrow neutrophils were isolated (Figure S1C, Supporting Information) and activated through succinate and lipopolysaccharide (LPS) stimulation. Activated neutrophil and macrophage membranes (ANM and MM, respectively) were mixed in an equal mass ratio.^[^ ^26^ ^]^ Hybrid membrane (HM)‐coated M4oRL (HM@MPN@4‐OI@liposomes, HM4oRL) NPs were then prepared with an Avanti filter, using a confocal laser scanning microscope (CLSM) approach to confirm their successful fabrication. Clear fluorescent colocalization was observed between Dio‐labeled ANM, Dil‐labeled MM, and IR780‐labeled MIrRL (MPN@IR780@liposomes, MIrRL), thus confirming the HM coating of these HMIrRL NPs following extrusion (Figure S1D, Supporting Information). The HM4oRL NPs measured 165.6 ± 9.45 nm (Figure 1A), with a zeta potential of ‐11.66 ± 2.48 mV, and a PDI of 0.19 ± 0.01, providing further confirmation of the HM coating of these NPs (Figure 1B‐C). The lyophilized powder yield for HM4oRL NPs was ≈51%. When visualized via transmission electron microscopy (TEM), the 4oRL, M4oRL, and HM4oRL NPs exhibited good monodispersivity and were spherical in shape. Negative phosphotungstic acid staining also revealed the presence of an outer membrane shell on the surfaces of these HM4oRL NPs (Figure 1D).

Fourier transform infrared spectra (FT‐IR) analyses revealed that 4‐Ol exhibited absorption peaks at 1724 cm^−1^ and 2850–2961 cm^−1^ respectively attributable to the C = O stretching vibration and the hydrophobic ‐CH_2_ group (Figure 1E). While the spectrum for 4oRL NPs exhibited these characteristic 4‐Ol peaks, their intensity levels were reduced, and additional characteristic peaks were evident at 1068 cm^−1^ and 545 cm^−1^ that were potentially attributable to PO_2_^−^ stretching vibrations and the S‐S bond of DSPE‐TK‐PEG.^[^ ^27^ ^]^ This combination of decreased 4‐Ol peaks and the emergence of additional peaks was consistent with successful liposomal 4‐Ol encapsulation. The spectrum for M4oRL NPs exhibited the characteristic 4oRL NP peaks without noticeable positional changes together with a novel peak at 1317 cm^−1^ attributable to O‐H bending vibrations in conjunction with Mn^2+^,^[^ ^28^ ^]^ suggesting that the internal structure of 4oRL was likely not altered by MPN modification (Figure 1E). X‐ray photoelectron spectroscopy scan spectra further revealed the presence of C, N, and O in 4oRL and M4oRL NPs at 290.5, 406.27, and 540.18 eV, respectively, with varying signal intensity levels (Figure 1F). The high‐resolution Mn 2p spectra for M4oRL NPs exhibited a peak at 640 eV consistent with the spectra of Mn (2p3/2) (Figure S1G, Supporting Information).^[^ ^29^ ^]^ Moreover, a significant increase in O 1s binding energy was noted for M4oRL NPs relative to 4oRL NPs, consistent with the complexation of the phenolic hydroxyl group with Mn^2+^ ions (Figure 1F).

Membrane proteins play a central role in the ability of cells to recognize and adhere to lesions. To clarify how neutrophil membrane protein expression was influenced by succinate exposure, transcriptomic analyses of total RNA from activated neutrophils were conducted. Succinate stimulation‐related functional changes were initially investigated through a Kyoto Encyclopedia of Genes and Genomes (KEGG) enrichment analysis, with the top 20 pathways most enriched for differentially expressed genes (DEGs) being presented in Figure S1E (Supporting Information). These activated neutrophils exhibited high expression of genes such as Ccl3, Cxcl2, and Icam1 (Figure S1F, Supporting Information). Succinate treatment was also associated with the induction of many genes associated with focal adhesion and ECM‐receptor interactions in neutrophils, including several upregulated integrins such as integrin α5 (ITGA5), integrin β1 (ITGB1), and integrin αX (ITGAX) (Figure 1H, G). Western blotting further confirmed that the majority of ANM‐ and MM‐derived membrane proteins were preserved in HM4oRL NPs (Figure 1I). These results highlight the successful use of succinate‐activated neutrophil membranes and macrophage membranes as the outer encapsulating layer of HM4oRL NPs.

The MPN film‐derived ROS scavenging capacity of the prepared HM4oRL NPs was next assessed, using MPN‐coated unloaded liposomes (MPN@unloaded@liposomes, MRL) of similar size and shape as a control (Figure S2, Supporting Information). An ABTS (2,2′‐azino‐bis (3‐ethylbenzothiazoline‐6‐sulfonic acid)) analysis of antioxidant activity revealed that M4oRL, MRL, and HM4oRL NPs all exhibited Trolox‐equivalent antioxidant capacity (TEAC) values exceeding 0.3 mM, while the values for 4‐Ol and 4oRL were below the limit of detection (Figure 1J). When the ^•^OH and ^•^O_2_^−^ scavenging capacity of these different preparations was assessed through an electron paramagnetic resonance (EPR) assay, reduced DMPO/^•^OH signal intensity was evident in the TA, MRL, and M4oRL NPs groups relative to the H_2_O_2_ group (Figure 1K), whereas there were no significant differences in signal intensity between the 4oRL NP and H_2_O_2_ groups (Figure 1K). Consistently, relative to the Xanthine + XO group, no apparent difference in BMPO/^•^O_2_^−^ signal intensity was evident in the 4oRL group, whereas it was lower in the TA, MRL, and M4oRL groups (Figure 1L). M4oRL and HM4oRL NPs are thus capable of robust ROS scavenging through a mechanism attributable to the TA within the MPN coating.

Analyses of the release of 4‐Ol from HM4oRL and M4oRL NPs in response to various stimuli were next conducted. A smooth 4‐Ol release profile from M4oRL NPs was noted in PBS (pH 6.4) or PBS containing 50 µM H_2_O_2_ (Figure 1M). Under conditions of dual exposure to an acidic pH and H_2_O_2_, cumulative 4‐OI release from M4oRL and HM4oRL NPs within 4 h reached 58.96% and 60.91%, respectively. The T1‐weighted MR signal‐enhancing performance of these HM4oRL NPs was further examined at different pH levels with a 9.4 T Micro MR scanner. Using different Mn concentrations (mM) and associated T1‐relaxation times, a regression equation was calculated in PBS at a pH of 7.4 as follows: y = 3.9464x + 0.4085 (R^2^ = 0.9948). When the pH of PBS was reduced to 6.4, the equation was as follows: y = 4.9464x + 0.4267 (R^2^ = 0.9996) (Figure 1N). HM4oRL NP stability was further assessed by storing these particles in PBS (pH 7.4), water, or cell culture medium containing 10% fetal bovine serum. Dynamic light scattering analyses revealed no significant changes in particle size or PDI over a 72‐h storage period, consistent with good HM4oRL NP stability (Figure S3, Supporting Information).

### 2.2 In Vitro Analyses of NP Cytotoxicity and Uptake

H9c2 cardiomyocytes, RAW 264.7 macrophages, and human umbilical vein endothelial cells (HUVECs) were used as models to test the cytotoxic effects of 4oRL, M4oRL, and HM4oRL NPs in vitro. At a 0.5 mg mL^−1^ dose level, all three of these NPs were associated with >80% H9c2, RAW 264.7, and HUVEC viability at 24 h in a CCK‐8 assay (Figure S4A‐C, Supporting Information).

IR780‐labeled MIrRL (MPN@IR780@liposomes, MIrRL) and HMIrRL (HM@MPN@IR780@liposomes, HMIrRL) NPs were next used to evaluate the uptake of these NPs by impaired cardiomyocytes (hypoxia‐exposed H9c2 cells) and inflammatory macrophages (LPS‐treated RAW 264.7 cells). Confocal imaging revealed time‐dependent increases in red fluorescence within LPS‐stimulated RAW 264.7 cells (Figure S5A, Supporting Information) and hypoxia‐induced H9c2 cells (Figure S5C, Supporting Information). At 12 h of treatment, flow cytometry revealed a 2.04‐fold increase in average fluorescence intensity for the HMIrRL group relative to the MIrRL group in RAW 264.7 cells (Figure S5B, Supporting Information) with a corresponding 1.45‐fold increase in H9c2 cells (Figure S5D, Supporting Information). These data thus support the superior affinity of modified HM‐coated NPs for impaired cardiomyocytes and inflammatory macrophages in vitro, consistent with potentially enhanced injured myocardial targeting activity.

### 2.3 HM4oRL NPs Attenuate Hypoxia‐Induced Cardiomyocyte Pyroptosis and Enhance Resistance to the Inflammatory Microenvironment In Vitro

**Figure 2:**
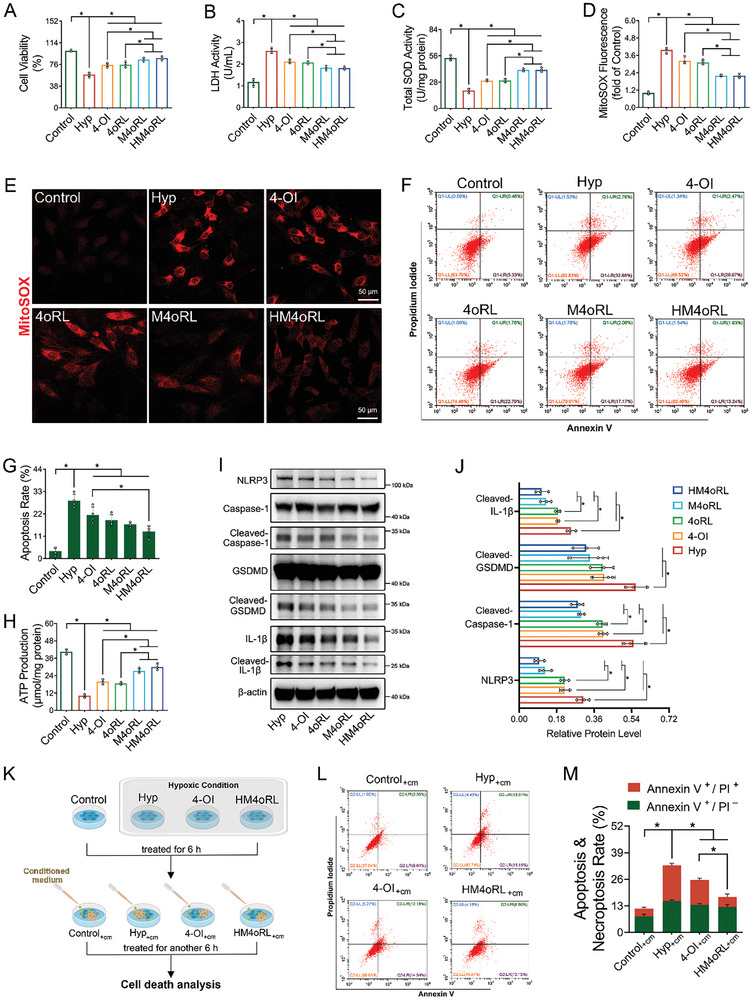
HM4oRL NPs attenuate hypoxia‐induced cardiomyocyte pyroptosis and enhance resistance to the inflammatory microenvironment in vitro. A) CCK‐8 for the relative viability of H9c2 cells. n = 5. B) LDH activity derived from the media of hypoxia‐induced H9c2 cells. n = 4. C) Total SOD activity in H9c2 cells. n = 4. Mitochondrial superoxide was revealed by the red fluorescence of MitoSOX via E) confocal fluorescence images and D) corresponding analysis using ImageJ software. n = 4. Cell apoptosis was assessed by flow cytometry F), and the apoptosis rate was presented in bar graphs G). n = 4. H) Analytic result of intracellular ATP levels in H9c2 cells. n = 4. I) Western blotting analysis of NLRP3, Caspase‐1, GSDMD, and IL‐1β protein expression in hypoxia‐induced H9c2 cells after different treatments. J) Quantitative analysis of NLRP3, Cleaved‐Caspase‐1, Cleaved‐GSDMD, and Cleaved‐ IL‐1β protein levels in each group of H9c2 cells. n = 3. K) Schematic illustration of the H9c2 cell model subjected to continuous stimuli in a post‐hypoxic inflammatory medium. The conditioned medium (cm) was obtained from the supernatants collected after RAW 264.7 cells were stimulated with 200 ng/mL of LPS for 24 h. L‐M) Cell death was analyzed by flow cytometry after various treatments indicated. n = 4. Data are presented as mean ± SD. Statistical methods: One‐way ANOVA with Tukey's post‐test (A‐D, G‐H, J, M). In all panels, * indicates *p* < 0.05.

Hypoxia is a central component of ischemia that can arise in the context of MI, coronary occlusion, or heart failure.^[^ ^30^ ^]^ To simulate this ischemic myocardial microenvironment, H9c2 cells were subjected to serum starvation and hypoxia as reported previously.^[^ ^31^ ^]^ After exposure to hypoxic conditions for 12 h, a sharp reduction in H9c2 cell viability to 59.12 ± 3.52% was noted (Figure S6A, Supporting Information), together with a pronounced increase in lactate dehydrogenase (LDH) levels in the culture medium to 2.79 ± 0.10 U mL^−1^ (Figure S6B, Supporting Information), consistent with cellular injury that was more severe than at earlier time points. When H9c2 cells were incubated with 4‐OI, 4oRL, M4oRL, or HM4oRL NPs from the onset of hypoxia, significantly improved viability was evident at 12 h, particularly among M4oRL and HM4oRL NP‐treated cells (**Figure** **2**A), with consistent results in an LDH release assay (Figure 2B). As the MPN coating of the M4oRL and HM4oRL NPs confers ROS scavenging activity, the antioxidant capacity and mitochondrial ROS levels in the hypoxia‐induced H9c2 cells in different treatment groups were assessed. Hypoxia markedly suppressed superoxide dismutase (SOD) antioxidant activity (Figure 2C) while enhancing mitochondrial ROS biogenesis (Figure 2D,E). Enhanced SOD activity and reduced mitochondrial ROS production, however, were observed when these hypoxia‐exposed H9c2 cells were treated with 4‐OI, 4oRL, M4oRL, or HM4oRL NPs. As ROS can trigger apoptotic and pyroptotic cell death, flow cytometry was used to evaluate apoptosis in these cells, revealing a significant rise in the frequency of apoptotic cells in the hypoxia model group to 28.43 ± 3.26% relative to the control group (Figure 2F,G). The 4‐OI, 4oRL, M4oRL, and HM4oRL treatments, however, markedly reduced these rates of apoptosis, with respective frequencies of 16.74 ± 1.21% and 13.29 ± 2.84% in the M4oRL and HM4oRL groups. Hypoxia exposure for 12 h was also associated with impaired ATP production in H9c2 cells consistent with the impairment of metabolic energy homeostasis, whereas 4‐OI, 4oRL, M4oRL, and HM4oRL NPs all enhanced ATP production, with the most robust benefits being evident in the M4oRL and HM4oRL groups (Figure 2H). Together, these data highlight the ability of 4‐Ol and 4‐Ol‐loaded NPs to mitigate hypoxia‐induced H9c2 cellular injury. Strikingly, the cardioprotective benefits of M4oRL and HM4oRL NPs were superior to the other tested treatments, as evidenced by significant reductions in mitochondrial ROS production and apoptotic cell death.

Western blotting analyses of NLRP3 inflammasome‐mediated pyroptosis‐related proteins were next conducted in hypoxia‐induced H9c2 cells, revealing marked reductions in NLRP3, cleaved‐Caspase‐1, cleaved‐GSDMD, and cleaved‐IL‐1β in H9c2 treated with HM4oRL NPs relative to the hypoxia model group (Figure 2I,J). Hypoxia also induced IL‐1β secretion from H9c2 cells, whereas 4‐OI, 4oRL, M4oRL, and HM4oRL NP treatments curtailed this IL‐1β secretion (Figure S7, Supporting Information). Together, these data highlight the ability of HM4oRL to suppress NLRP3 inflammasome‐mediated pyroptosis in hypoxia‐exposed H9c2 cells.

The effects of inflammation on injured cardiomyocyte viability were modeled in vitro by treating hypoxia‐injured H9c2 cardiomyocytes with inflammatory medium (Figure 2K). Significantly increased necroptosis (Annexin V^+^/Pl^+^) was evident among hypoxia‐damaged necroptosis upon inflammatory medium exposure (Figure 2L‐M), suggesting that injured cardiomyocytes are more sensitive to the inflammatory microenvironment, contributing to more severe cellular damage. HM4oRL NPs treatment, however, protected against this inflammation‐mediated exacerbation of cytotoxicity as evidenced by a reduction in the frequency of necroptotic cardiomyocytes (Figure 2L‐M). HM4oRL administration can thus strengthen the ability of injured cardiomyocytes to target exposure to an inflammatory microenvironment.

To confirm the cardioprotective benefits of HM4oRL treatment, H9c2 cells were subjected to prolonged hypoxia, followed by delated intervention (Figures S8A and S9A, Supporting Information). CCK‐8, LDH, and PI staining results revealed that prolonged hypoxia induced significantly more severe H9c2 cellular injury (Figure S8B‐E, Supporting Information), with necroptosis affecting 22.31 ± 1.13% of these H9c2 cells (Figure S9B‐C, Supporting Information). Even when administered in a delayed manner after hypoxia, HM4oRL was able to rescue hypoxia and inflammation‐induced H9c2 cell death, as demonstrated by reduced Pl staining positivity (Figure S8D, Supporting Information) and necroptosis (Figure S9B, Supporting Information).

### 2.4 HM4oRL Treatment Restrains LPS‐Induced RAW 264.7 Macrophage Inflammation

As macrophages are the key cellular mediators of both injury and repair in the myocardium,^[^ ^12^ ^]^ the impact of 4‐Ol and the NPs prepared above on LPS‐stimulated macrophage functions was next assessed. LPS‐stimulated RAW 264.7 macrophages exhibited significantly increased intracellular ROS biogenesis (Figure S10A‐C, Supporting Information) coupled with the enhanced secretion of IL‐1β (Figure S10D, Supporting Information), tumor necrosis factor (TNF)‐α (Figure S10E, Supporting Information), and IL‐10 (Figure S10F, Supporting Information). While treatment with 4‐OI or with 4oRL, M4oRL, or HM4oRL NPs significantly suppressed ROS, IL‐1β, and TNF‐α levels in this system, significantly higher IL‐10 levels were noted in the M4oRL and HM4oRL groups relative to the LPS, 4‐OI, or 4oRL groups. These results suggest that treatment with HM4oRL NPs can directly regulate pro‐inflammatory macrophage activity.

### 2.5 HM4oRL NPs Exhibit Cardiac Targeting and T1‐Weighted MR Imaging Enhancement in AMI Model Mice

**Figure 3:**
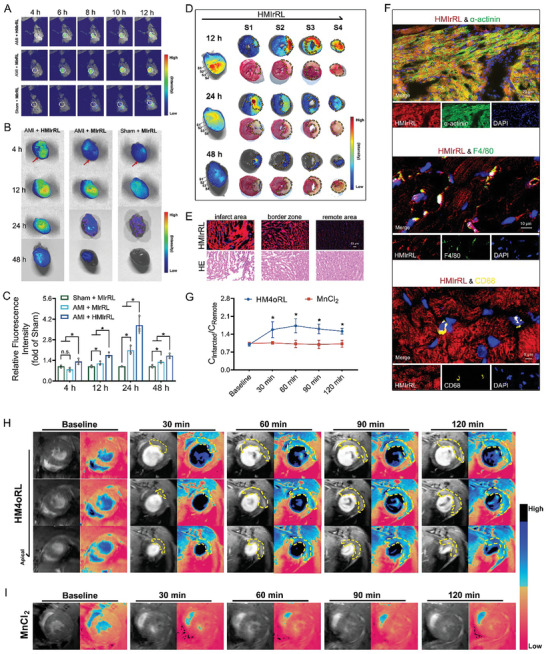
Targeted delivery and cardiac T1‐weighted MR imaging of HM4oRL in AMI model mice. A) In vivo NIRF fluorescence imaging of AMI or Sham‐operated mice at the indicated time point after intravenous administration of HMIrRL or MIrRL nanoparticles. B) Ex vivo NIRF fluorescence imaging of heart samples harvested from AMI or Sham‐operated mice at the indicated time point after the onset of coronary ligation, and C) quantification analysis of the fluorescence intensity in the infarcted area by ImageJ software. n = 3. * indicates *p* < 0.05. D) Heart samples were collected at the indicated time point after intravenous administration of HMIrRL in AMI mice. Subsequently, ex vivo NIRF fluorescence imaging of the collected heart and its slices was performed, followed by TTC staining of the corresponding slices. E) Representative fluorescence images and corresponding HE‐stained images of the infarct area, border zone, and remote area of the AMI heart after injection of HMIrRL nanoparticles. F) Heart samples were collected 12 h after intravenous administration of HMIrRL in AMI mice. Immunofluorescent staining was performed for cardiomyocytes (α‐actinin) and monocytes/macrophages (CD68, F4/80). Green, α‐actinin or F4/80; yellow, CD68; red, nanoparticles; blue, DAPI. H) Representative in vivo MR imaging of AMI (yellow dashed area) before (baseline) and post i.v. injection of HM4oRL for 30, 60, 90, and 120 min on 9.4 T Micro MR scanner. I) In vivo cardiac MR imaging of AMI before (baseline) and post i.v. injection of MnCl_2_ for the first 120 min. G) Quantitative analysis of CNR in the infarcted versus remote myocardium of MI mice, during the first 120 minutes following the injection of HM4oRL nanoparticles and MnCl_2_. * indicates when compared with Baseline, *p* < 0.05. n = 4. Data are presented as mean ± SD. Statistical methods: One‐way ANOVA with Tamhane's post‐test (C) and Independent Samples Tests (G). In all panels, * indicates *p* < 0.05, and “n. s.” indicates no significance.

The ability of the prepared HM‐coated NPs to target the infarcted myocardium during the early phase of AMI was next investigated in a mouse model of AMI. An in vivo imaging system (IVIS) revealed that intravenously administered MIrRL and HMIrRL NPs accumulated in the precordial region (yellow dashed area) from 4 to 12 h post‐injection in AMI‐operated mice (**Figure** **3**A). Isolated cardiac tissues were further analyzed to assess the fluorescent signal attributable to these MIrRL and HMIrRL NPs in order to assess their early‐stage myocardial targeting and retention at 4, 12, 24, and 48 h post‐AMI. At 4 h post‐injection, the average fluorescence intensity at the infarcted site in the AMI + MIrRL group was lower than that in the Sham + MIrRL group whereas it was significantly higher in the AMI + HMIrRL group (Figure 3B‐C). Infarcted tissue fluorescent signal intensity at 12, 24, and 48 h was significantly higher in the AMI + HMIrRL group relative to the AMI + MIrRL group, suggesting that HM modification endowed these NPs with properties amenable to superior cardiac accumulation and retention. Ischemic targeting activity was further assessed through TTC staining at 12, 24, and 48 h post‐injection, revealing a strong correlation between TTC‐positive areas (yellow dashed regions) and fluorescence intensity (Figure 3D). Red fluorescent HMIrRL signal was detectable at both the infarcted sizes and border regions within the heart after AMI (Figure 3E), supporting the specific accumulation of HMIrRL NPs in infarcted areas. Broader analyses of NP biodistributions across major organs revealed that the liver was the primary site of IR780 in all three experimental groups (Figure S11, Supporting Information), in line with findings for many nanoformulations.^[^ ^32^ ^]^ Analyses of the cellular co‐localization of HMIrRL NPs were performed using marker proteins for cardiomyocytes (α‐actinin) and monocytes/macrophages (CD68, F4/80), revealing the presence of these NPs within both of these cell populations consistent with successful HMIrRL NP uptake in the ischemic region (Figure 3F). Together, these results highlight the ability of HM modification to enhance NP targeting to the injured myocardium during the early post‐AMI phase.

Given the striking cardiac targeting activity of HM4oRL and its high r1 values, a 9.4 T Micro MR scanner was next used for in vivo MR imaging, using MnCl_2_ with an equivalent Mn concentration as a control treatment. At 30–120 min after HM4oRL injection, the infarcted site exhibited striking signal enhancement (marked with a yellow dashed line) (Figure 3H), whereas MnCl_2_ contrast enhancement was less effective as a means of identifying the infarcted sites (Figure 3I). HM4oRL injection rapidly increased the contrast‐to‐noise ratio (CNR) for infarcted myocardium relative to remote myocardial tissue after injection (Figure 3G). Beyond any cardioprotective benefits, these cardiac‐targeted HM4oRL NPs can thus provide additional value as a novel T1‐weighted MRI contrast agent to support AMI diagnosis.

### 2.6 HM4oRL Suppresses the Priming and Activation of Post‐MI NLRP3 Inflammasome‐Mediated Pyroptosis

Pyroptosis is the predominant form of PCD observed in cardiac myocytes during the early stages of AMI.^[^ ^4^ ^]^ In light of the dynamics of pyroptotic induction, the duration of the acute inflammatory phase, and the observed HM4oRL retention in the injured myocardium, an HM4oRL multi‐injection plan was developed (Figure S12A, Supporting Information). Following the planned administration of a range of HM4oRL NP doses in MI model mice, therapeutic efficacy was evaluated via echocardiography. These analyses led to the selection of the administration of 10 mg kg^−1^ HM4oRL NPs cross four injections as a safe and effective therapeutic approach, as this dosing strategy was associated with significantly improved left ventricular ejection fraction (LVEF) without any apparent adverse reactions (Figure S12B‐M, Supporting Information).

**Figure 4:**
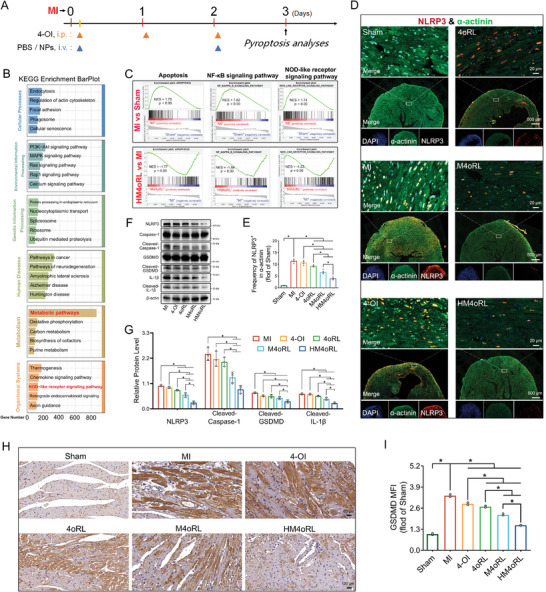
HM4oRL suppresses the priming and activation of post‐MI NLRP3 inflammasome‐mediated pyroptosis. A) Schematic illustration of the experimental timelines for short‐term studies of MI mice. B) KEGG enrichment analyses of differentially expressed genes, obtained from transcriptome sequencing of infarcted myocardium in the Sham versus MI versus HM4oRL groups. n = 4. C) GSEA analysis of “Apoptosis”, “NF‐κB signaling pathway”, “NOD‐like receptor signaling pathway” related enrichment plots derived from transcriptome sequencing in MI versus Sham and HM4oRL versus MI groups. n = 4. D) Representative immunofluorescence micrographs of NLRP3^+^ and α‐actinin^+^ in the infarct regions, and E) quantification of NLRP3‐positive within α‐actinin‐positive cells of each group. n = 4. Green, α‐actinin; red, NLRP3; blue, DAPI. F) Western blotting of NLRP3, Caspase‐1, GSDMD, and IL‐1β protein expression in the infarcted myocardium from each group. G) Corresponding quantification of NLRP3, Cleaved‐Caspase‐1, Cleaved‐GSDMD, and Cleaved‐IL‐1β protein levels in each group. n = 3. H) Immunohistochemical staining and I) quantification of GSDMD‐positive fluorescence intensity in the infarcted area following different treatments. n = 4. Data are presented as mean ± SD. Statistical methods: One‐way ANOVA with Tukey's post‐test (E, G, I). In all panels, * indicates *p* < 0.05.

We then comprehensively explored the mechanisms that underlie the cardioprotective effects of 4‐OI and 4‐OI‐loaded NP in a mouse model of MI. On day 3 post‐MI (Figure **4**A), significantly increased serum creatine kinase‐MB (CK‐MB) levels were observed (Figure S13A, Supporting Information), together with significantly reduced SOD activity in the infarcted myocardium (Figure S13B, Supporting Information). Treatment with HM4oRL NPs markedly abrogated this increase in CK‐MB levels while increasing SOD activity in these MI model animals. Transcriptomic sequencing was subsequently used to gain a broader perspective on the mechanisms of cardioprotection mediated by these HM4oRL NPs. In total, comparisons of the Sham versus MI versus HM4oRL treatment groups led to the identification of 10 095 DEGs (*p* < 0.05 & *q* < 0.05) (Figure 4B). KEGG pathway analyses suggested that these DEGs were enriched in metabolic pathways (Figure 4B). Strikingly, the NOD‐like receptor signaling pathway was ranked third in the organismal systems category, encompassing a total of 122 DEGs (Figure 4B). This suggests that HM4oRL treatment may significantly influence post‐MI pyroptotic activity. Moreover, the top 30 significantly enriched gene set enrichment analysis (GSEA) pathways between the groups are presented in Figure S14 (Supporting Information). It is well‐known that NF‐κB plays a regulatory role in the priming of the NLRP3 inflammasome.^[^ ^33^ ^]^ Then, GSEA analysis was performed on pyroptosis and its priming‐related signaling pathways, including the “NF‐κB signaling pathway” and “Apoptosis”. The results of the GSEA analysis on the “NOD‐like receptor signaling pathway” indicated a normalized enrichment score (NES) of ‐1.23 and a *p*‐value of 0.09, in the comparison between HM4oRL and MI groups (Figure 4C). Treatment with HM4oRL effectively suppressed the upregulation of “NF‐κB signaling pathway” and “Apoptosis”‐associated genes induced by MI (Figure 4C). Collectively, the results of these transcriptomic analyses suggest that HM4oRL may mediate pyroptotic priming through NF‐κB‐related gene inhibition.

The mechanistic basis for pyroptotic activation was also confirmed, as MI modeling was associated with a dramatic increase in the frequency of apoptotic cardiomyocytes (Figure S13C‐D, Supporting Information), coinciding with increased NLRP3 expression (Figure 4D,E). Analyses of NLRP3 and α‐actinin co‐localization revealed high NLRP3 expression levels around the ischemic border zone in the MI group, while the same was not evident in the 4oRL, M4oRL, or HM4oRL groups (Figure 4D). Post‐MI cardiomyocyte apoptosis was markedly suppressed by 4oRL, M4oRL, or HM4oRL NP treatment (Figure S13C, Supporting Information), with a corresponding reduction in NLRP3 expression (Figure 4E). Pyroptosis‐related protein expression was also assessed in the infarcted tissue, revealing significant reductions in NLRP3, cleaved‐Caspase‐1, cleaved‐GSDMD, and cleaved IL‐1β in the HM4oRL group relative to the MI group (Figure 4F,G). Furthermore, Anti‐GSDMD staining revealed a significant reduction in GSDMD mean fluorescence intensity under conditions of 4‐OI, 4oRL, M4oRL, or HM4oRL NP treatment (Figure 4H,I). Together, these data suggest that the cardioprotective activity of HM4oRL treatment is partially attributable to the inhibition of NLRP3 inflammasome priming and activation following MI.

### 2.7 HM4oRL Preserves Cardiac Metabolic Microenvironment Homeostasis and Inhibits Pro‐Inflammatory Macrophage Responses after MI

**Figure 5:**
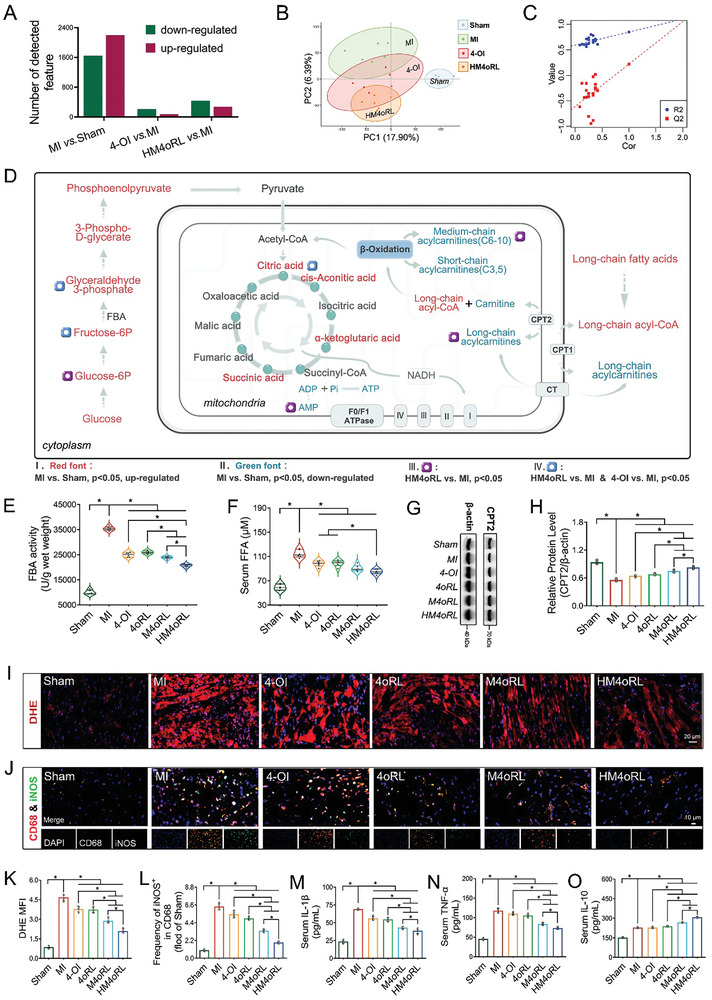
HM4oRL preserves cardiac metabolic microenvironment homeostasis and inhibits pro‐inflammatory macrophage responses after MI. A) The numbers of identified metabolites from metabolomics of infarcted myocardium in each group 3 days after MI. B) The PLS‐DA score plot of the Sham, MI, 4‐OI, and HM4oRL groups, and C) the corresponding validation plot. D) Potential metabolic pathways for glucose, fatty acid β‐oxidation and tricarboxylic acid cycle derived from metabolomics of the infarcted myocardium. Glucose‐6P: glucose‐6‐phosphate; Fructose‐6P: fructose‐6‐phosphate; Acetyl‐CoA: acetyl‐coenzyme A; Succinyl‐CoA: succinyl‐coenzyme A; CPT1: carnitine palmitoyltransferase 1; CPT2: carnitine palmitoyltransferase 2; I‐IV: mitochondrial complex I‐IV; NADH: nicotinamide adenine dinucleotide. n = 6. E) Levels of FBA activity in injured myocardium after different treatments. n = 6. F) Levels of serum FFA after different treatments. n = 6. G‐H) Western blotting analysis of CPT2 protein content alterations in injured myocardium after different treatments. n = 4. I) The ROS production assessed by DHE staining of mice heart sections, and K) quantification of ROS levels in the infarcted area of each group by ImageJ software. Red, DHE. n = 4. J) Representative double‐immunofluorescence staining for CD68 and iNOS in infarct tissue after MI, and L) quantification of iNOS^+^ CD68 macrophages for each group by ImageJ software. Green, iNOS; red, CD68; blue, DAPI. n = 4. M‐O) Serum IL‐1β, TNF‐α, and IL‐10 levels from each group. n = 4. Data were presented as mean ± SD. Statistical methods: One‐way ANOVA with Tukey's post‐test (E‐F, H, K‐O). In all panels, * indicates *p* < 0.05.

In transcriptomic analyses, HM4oRL treatment was associated with significant changes in metabolic pathway‐related gene expression following MI. Metabolic activity is central to cellular physiology, and changes in metabolite accumulation can shape local macrophage functionality.^[^ ^34^ ^]^ Thus, we explored the impact of HM4oRL on the metabolic processes of the infarcted myocardial tissue via untargeted metabolomics. The identified metabolites from each group are shown in Figure **5**A, while the heat map of hierarchical cluster analysis is presented in Figure S15A (Supporting Information). The partial least squares discriminant analysis (PLS‐DA) model exhibited no evidence of overfitting (R^2^X > 0.5, Q^2^ < 0), and the model was able to significantly discriminate among the Sham, MI, and HM4oRL groups (Figure 5B,C). Differentially abundant metabolites (DAMs) (*p* < 0.05, FC ≥ 2 or ≤ 0.5, and VIP ≥ 2) in the infarcted myocardium of MI, 4‐OI, and HM4oRL groups are presented in Figure S15B‐D (Supporting Information), respectively. Pronounced enrichment of metabolites classified in the “lipids and lipid‐like molecules” category was observed, followed by those in the “nucleosides, nucleotides, and analogues” and “organic oxygen compounds” categories. Under basal conditions, energy generation in the adult myocardium is primarily mediated by fatty acid (FA) β‐oxidation. Under hypoxic and ischemic conditions, however, the myocardium shifts to favor glucose as the primary substrate for energy generation. The specific metabolites of interest and potential 4‐Ol targets identified in this study were used to generate a pathway model including glycolysis, TAC, and FA β‐oxidation as key metabolic mechanisms relevant in this system (Figure 5D; Figure S16, Supporting Information). Relative to the sham group, the MI group presented with increases in the levels of most glycolysis metabolism pathway‐related metabolites, including glucose 6‐phosphate (Glucose‐6P), fructose 6‐phosphate (Fructose‐6P), and glyceraldehyde 3‐phosphate. While long‐chain fatty acids levels were also significantly elevated in the MI group, a significant decrease in the levels of downstream metabolites associated with the FA β‐oxidation pathway, including acylcarnitines and carnitine, was also noted. A significant increase in several TAC‐related metabolites was also noted, including citric acid, cis‐aconitic acid, and α‐ketoglutaric acid. Meanwhile, the nucleotide metabolites in the MI group, including AMP, Pi, ADP, and ATP, demonstrated a consistent and significant reduction. Treatment with 4‐Ol alone significantly reduced Fructose‐6P, glyceraldehyde 3‐phosphate and citric acid levels, whereas HM4oRL NP treatment additionally had a significant effect on Glucose‐6P, acylcarnitines, and AMP levels (Figure S16, Supporting Information). These findings align well with the cardiac‐targeted delivery of 4‐Ol facilitating significant improvements in the ability of these NPs to regulate metabolic activity within the injured myocardium.

Further GSEA analyses of transcriptomic data were conducted to validate the metabolism‐related transcriptomic signatures associated with HM4oRL treatment, revealing that the administration of these NPs effectively reversed MI‐related changes in metabolic processes including “ATP biosynthetic process”, “Fatty acid beta‐oxidation”, “Citrate cycle (TCA cycle)”, “Glycolysis/gluconeogenesis”, and “Oxidative phosphorylation” (Figure S17, Supporting Information).

Besides, we observed a significant elevation in the levels of Glucose‐6P and glyceraldehyde 3‐phosphate within the glycolytic pathway following MI. Previous studies have confirmed that fructose‐bisphosphate aldolase (FBA) is a key enzyme catalyzing the conversion of Fructose‐6P to glyceraldehyde‐3‐phosphate and is also a target of 4‐OI.^[^ ^16b^ ^]^ Consequently, we quantified FBA activity in myocardial tissue. As shown in Figure 5E, the FBA activity in the MI group increased by more than threefold, while it demonstrated varying degrees of decline across the treatment groups. Furthermore, we integrated transcriptomics to gain deeper insights into the potential mechanisms by which HM4oRL affects lipid metabolism in injured myocardium. GSEA analysis revealed a significant downregulation of genes associated with fatty acid metabolism (Figure S18, Supporting Information). Notably, the carnitine palmitoyltransferase 2 (CPT2), a key protein that facilitates myocardial fatty acid β‐oxidation, showed the most pronounced decrease.^[^ ^35^ ^]^ However, treatment with HM4oRL significantly enhanced CPT2 expression in the injured myocardium (Figure 5G,H) and reduced serum free fatty acid (FFA) levels following MI (Figure 5F). Collectively, these results provide support for the ability of HM4oRL NPs to preserve the stability of the metabolic microenvironment through properties attributable to their ability to modulate cardiac metabolic activity.

Macrophages can undergo various forms of phenotypic and functional polarization in response to local microenvironmental conditions.^[^ ^36^ ^]^ Pro‐inflammatory macrophages are more reliant on glycolytic activity for energy generation, exhibiting impaired TAC and oxidative phosphorylation activity.^[^ ^11^ ^]^ These metabolic features contribute to increased ROS levels within these cells coupled with higher levels of inflammatory IL‐1β and TNF‐α secretion.^[^ ^37^ ^]^ Dihydroethidium (DHE) staining revealed markedly elevated ROS levels in the infarcted myocardium relative to sham‐operated mice (Figure 5I,K), while 4‐OI, 4oRL, M4oRL, or HM4oRL NP treatment reduced ROS levels within the damaged myocardium. Given that HM4oRL NPs affect the metabolic microenvironment and ROS biogenesis, these NPs may affect the post‐MI phenotypic transformation of cardiac macrophages. Relative to sham control mice, PBS‐treated MI model animals presented with a significant increase in the CD68^+^/iNOS^+^ macrophage ratio in the infarcted area (Figure 5J,L), together with substantially enhanced IL‐1β (Figure 5M), TNF‐α (Figure 5N), and IL‐10 secretion (Figure 5O). HM4oRL NP treatment, however, significantly reduced the CD68^+^/iNOS^+^ macrophage ratio and suppressed IL‐1β and TNF‐α secretion more readily than 4‐Ol alone while increasing post‐MI IL‐10 serum levels.

### 2.8 HM4oRL Activated AMPK Pathway Signaling In Vivo and In Vitro

**Figure 6:**
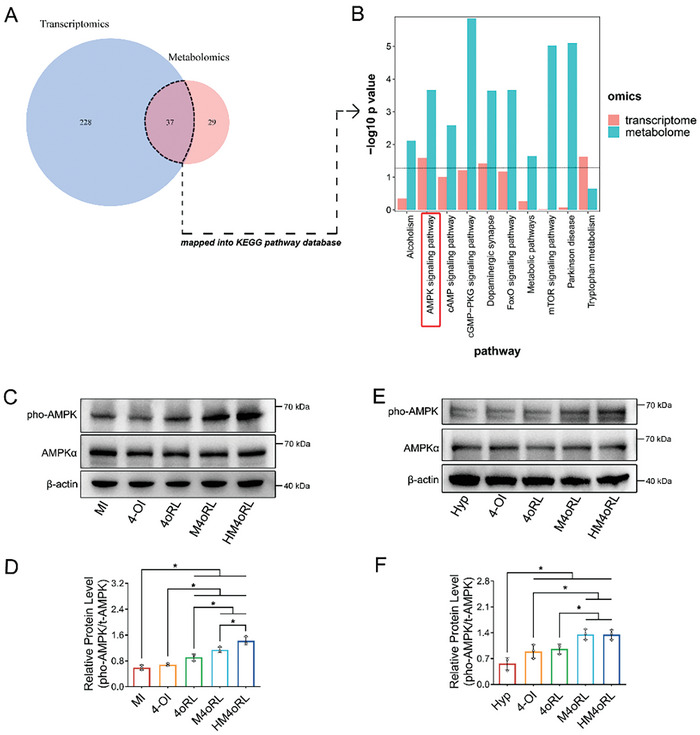
HM4oRL activated AMPK pathway signaling in vivo and in vitro. A) A Venn diagram intersecting the differentially expressed genes from transcriptomics with differentially expressed metabolites from metabolomics in MI versus HM4oRL groups. B) KEGG pathway enrichment analyses of 37 differentially expressed genes and differentially expressed metabolites. The AMPK and pho‐AMPK protein expression in C‐D) the infarcted myocardium, and E‐F) the hypoxia‐treated H9c2 cells were detected by Western blotting and quantified by ImageJ software. n = 3. Data are presented as mean ± SD. Statistical methods: One‐way ANOVA with Tukey's post‐test (D, F). In all panels, * indicates *p* < 0.05.

To clarify the molecular mechanisms through which HM4oRL regulates post‐AMI cardiac pyroptosis and metabolic activity, integrated metabolomics and transcriptomics analyses were next performed. In total, 265 DEGs were identified when comparing the HM4oRL and MI groups (*p* < 0.05 and FC ≥ 2 or ≤ 0.5), as were 66 DAMs (*p* < 0.05, FC ≥ 2 or ≤ 0.5, and VIP ≥ 1). Then, 37 DAMs and DEGs were then combined for KEGG database mapping (**Figure** **6**A), yielding 10 common signaling pathways (Figure 6B). The AMP‐activated protein kinase (AMPK) signaling and dopaminergic synapse pathways exhibited significance in both the metabolomics and transcriptomic analyses. AMPK is a master regulator of metabolic activity that is also closely tied to NLRP3 inflammasome activation.^[^ ^38^ ^]^ The transcriptomic analysis did not reveal any significant changes in AMPK messenger RNA levels; therefore, we performed Western blotting to investigate the post‐transcriptional modification of AMPK. It was found that 4oRL, M4oRL, and HM4oRL NPs treatment significantly increased the phosphorylation levels of AMPK in the infarcted myocardial tissue (Figure 6C‐D) and hypoxic cardiomyocytes (Figure 6E‐F). Together, these data support the ability of HM4oRL to mediate AMPK pathway activation via the enhancement of AMPK phosphorylation.

### 2.9 HM4oRL Suppresses Myocardial Ly6C^high^CCR2^high^CX3CR1^low^ Monocyte Infiltration and Favors Reparative CRM Phenotypes after MI

**Figure 7:**
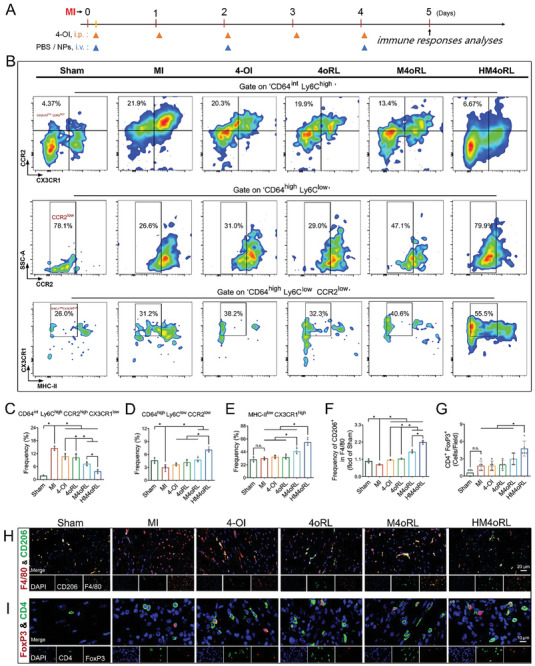
HM4oRL suppresses myocardial Ly6C^high^CCR2^high^CX3CR1^low^ monocyte infiltration and favors reparative CRM phenotypes after MI. A) Schematic illustration of the experimental timelines for short‐term studies of MI mice. B) Gating strategy for cardiac monocytes/macrophages and its subsets in mice hearts of each group after MI. C) Flow cytometry–based quantification of recruited monocytes CD64^int^Ly6C^high^CCR2^high^CX3CR1^low^ in live CD45^+^ population. n = 4. D) Flow cytometry–based quantification of CD64^high^Ly6C^low^CCR2^low^ monocytes/macrophages in live CD45^+^ population. n = 4. E) Flow cytometry–based quantification of MHC‐II^low^CX3CR1^high^ cardiac‐resident macrophages in CD64^high^Ly6C^low^CCR2^low^ monocytes/macrophages population. n = 4. H) Representative double‐immunofluorescence staining for F4/80 and CD206 in infarct tissue after MI, and F) quantification of CD206^+^ macrophages for each group by ImageJ software. Green, CD206; red, F4/80; blue, DAPI. n = 4. I) Representative double‐immunofluorescence staining for CD4 and FoxP3 in infarct tissue after MI, and G) quantification of CD4^+^ FoxP3^+^ cells for each group. Green, CD4; red, FoxP3; blue, DAPI. n = 5. Data are presented as mean ± SD. Statistical methods: One‐way ANOVA with Tukey's post‐test (C‐G). In all panels, * indicates *p* < 0.05, and “n. s.” indicates no significance.

As HM4oRL NPs prevented pyroptotic induction during the early phases of AMI, preserved metabolic homeostasis, and altered macrophage functionality, we hypothesized that these NPs may influence immune response regarding the infiltration and differentiation of monocytes in infarcted heart tissues. Recruited circulation‐derived Ly6C^high^CCR2^high^ monocytes, which express lower levels of CX3CR1, are a major source of immune cells that exacerbate inflammation within the injured myocardium.^[^ ^39^ ^]^ Here, flow cytometry was next used to analyze monocyte (CD64^int^Ly6C^high^CCR2^high^CX3CR1^low^) recruitment from the ventricular myocardium at day 5 post‐MI (**Figure** **7**A; Figure S19, Supporting Information). Relative to Sham control mice, the MI group exhibited significantly higher proportions of CD64^int^Ly6C^high^CCR2^high^CX3CR1^low^ monocytes among live CD45^+^ cells (Figure 7B‐C). However, 4‐OI, 4oRL, M4oRL, or HM4oRL NP treatment reversed these changes in monocyte frequencies. In contrast, cardiac Ly6C^low^CCR2^low^ monocytes/macrophages are the major subtype of cells involved in myocardial healing.^[^ ^40^ ^]^ Mice treated with M4oRL and HM4oRL NPs exhibited significant increases in the frequencies of CD64^high^Ly6C^low^CCR2^low^ monocytes among live CD45^+^ cells relative to the MI model animals (Figure 7B,D). Moreover, CRMs, which are a unique subset of cardiac immune cells that arise from both circulation and local self‐renewal, are key mediators of homeostatic maintenance within the immune microenvironment.^[^ ^41^ ^]^ Recently, Chen et al. classified CRMs into four subsets based on the macrophage surface makers Ly6C, CCR2, CX3CR1, and MHC‐II.^[^ ^42^ ^]^ The yolk sac, fetal liver, and bone marrow‐derived Ly6C**^−^** CCR2**^−^** CX3CR1**^+^** MHC‐II**^−^** subset plays important roles in cardiac development, homeostasis, and regeneration. Following AMI, these Ly6C**^−^** CCR2**^−^** CX3CR1**^+^** MHC‐II**^−^** cells transition into Ly6C**^−^** CCR2**^−^** CX3CR1**^+^** MHC‐II**^+^** cells that stimulate inflammation development. Efforts were thus made to differentiate MHC‐II^low^CX3CR1^high^ CRMs into CD64^high^Ly6C^low^CCR2^low^ monocytes/macrophages. A significantly higher proportion of MHC‐II^low^CX3CR1^high^ CRMs was observed in the HM4oRL group relative to the MI, 4‐OI, 4oRL and M4oRL groups (Figure 7B,E).

CD206 is a surface marker for reparative macrophages. In co‐localization assays staining for CD206 and F4/80, 4‐OI, 4oRL, M4oRL, or HM4oRL NP treatments all led to a significant increase in CD206^+^/F4/80^+^ macrophage numbers in the infarcted area (Figure 7F,H). T cells, particularly regulatory T cells, have been widely studied as key suppressors of myocardial inflammation that help maintain immunological homeostasis following MI. HM4oRL treatment significantly increased the frequency of CD4^+^Foxp3^+^ cells in the infarcted area (Figure 7G,I). Together, these data support the ability of HM4oRL NPs to significantly suppress cardiac CD64^int^Ly6C^high^CCR2^high^CX3CR1^low^ monocyte infiltration while increasing the abundance of cardiac CD64^high^Ly6C^low^CCR2^low^ monocytes/macrophages and MHC‐II^low^CX3CR1^high^ CRMs.

### 2.10 HM4oRL Reduces Infarct Sizes and Promotes Cardiac Repair after MI

**Figure 8:**
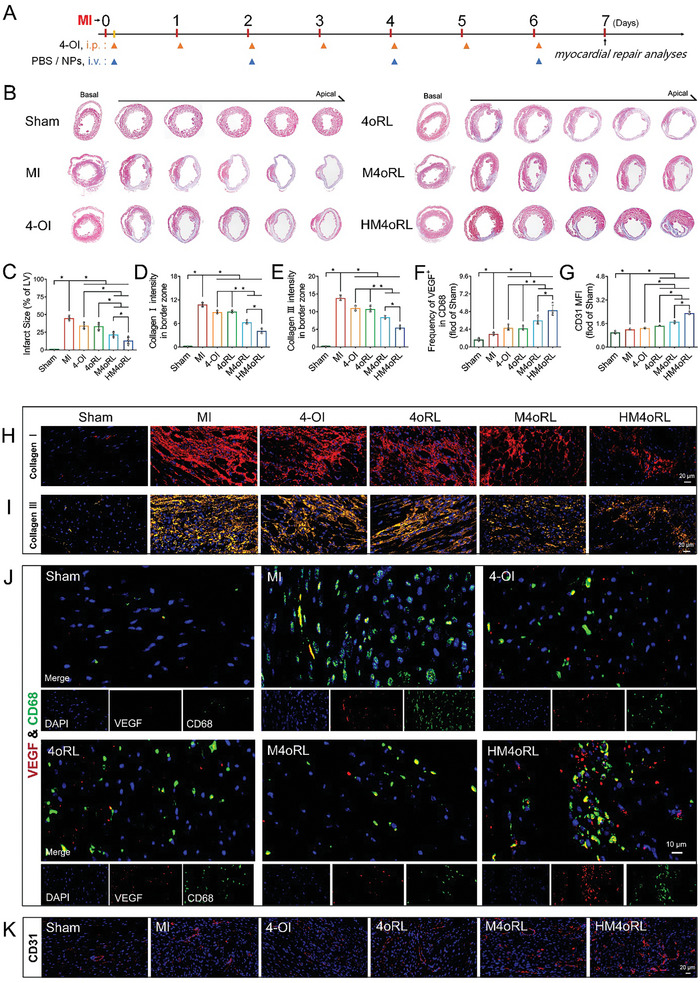
HM4oRL reduces infarct sizes and promotes cardiac repair after MI. A) Schematic illustration of the experimental timelines for short‐term studies of MI mice. B) Representative images of Masson's trichrome‐stained sections of infarcted hearts 7 days after treatment, and C) quantification of scar size for each group by ImageJ software. n = 6. Immunostaining analyses of D, H) collagen I (red), and E, I) collagen III (yellow) in border zone of infarct tissue. Blue, DAPI. n = 4. J) Representative double‐immunofluorescence staining for VEGF and CD68 in infarcted tissue after MI, and F) quantification of VEGF^+^ macrophages for each group by ImageJ software. Green, CD68; red, VEGF; blue, DAPI. n = 4. G, K) Immunostaining analyses of CD31 in the infarct zone. Red, CD31; blue, DAPI. n = 4.Data are presented as mean ± SD. Statistical methods: One‐way ANOVA with Tukey's post‐test (C‐G). In all panels, * indicates *p* < 0.05.

As HM4oRL NPs exhibited pronounced cardioprotective effects and were capable of reprogramming immune inflammatory responses, myocardial injury and repair were next assessed during the acute phase of MI. At day 7 post‐MI (**Figure** **8**A), the infarct size in the MI group was 45.14 ± 2.55%, whereas treatment with 4‐OI, 4oRL, M4oRL, and HM4oRL NPs reduced the scar size to 34.40 ± 4.10%, 33.71 ± 4.88%, 21.97 ± 3.12%, and 13.37 ± 4.63%, respectively (Figure 8B,C). The neovascularization of the infarcted area helps restore oxygenated blood flow to the local area, thereby mediating cardiac repair. Moderate collagen deposition also helps preserve the structural integrity of the infarcted myocardium.^[^ ^43^ ^]^ Collagen I and III density in the border zone was significantly increased in the MI group relative to the Sham group (Figure 8H,I), while 4‐OI, 4oRL, M4oRL, and HM4oRL NPs decreased the density of these two collagen subtypes relative to the MI group (Figure 8D,H,E,I). Cardiac reparative macrophages are key drivers of angiogenic activity, which is vital for post‐AMI myocardial repair.^[^ ^44^ ^]^ HM4oRL NPs were able to readily suppress pro‐inflammatory monocyte infiltration and to increase the frequency of CRMs with a reparative phenotype, suggesting that these NPs may shape angiogenic activity. VEGF, which is primarily secreted by reparative macrophages, is a key mediator of angiogenesis.^[^ ^45^ ^]^ The HM4oRL NP treatment group exhibited the highest VEGF‐positive macrophages (Figure 8F,J). CD31 staining for coronary capillary density in the infarcted area additionally revealed significantly higher levels of CD31 fluorescence density in the 4oRL, M4oRL, and HM4oRL groups relative to the MI group (Figure 8G,K). These results suggest that intravenous HM4oRL NP delivery can effectively reduce infarct size, suppress excessive collagen deposition in the border zone, and promote angiogenesis following MI.

### 2.11 HM4oRL Enhances Long‐Term Cardiac Function after MI

**Figure 9:**
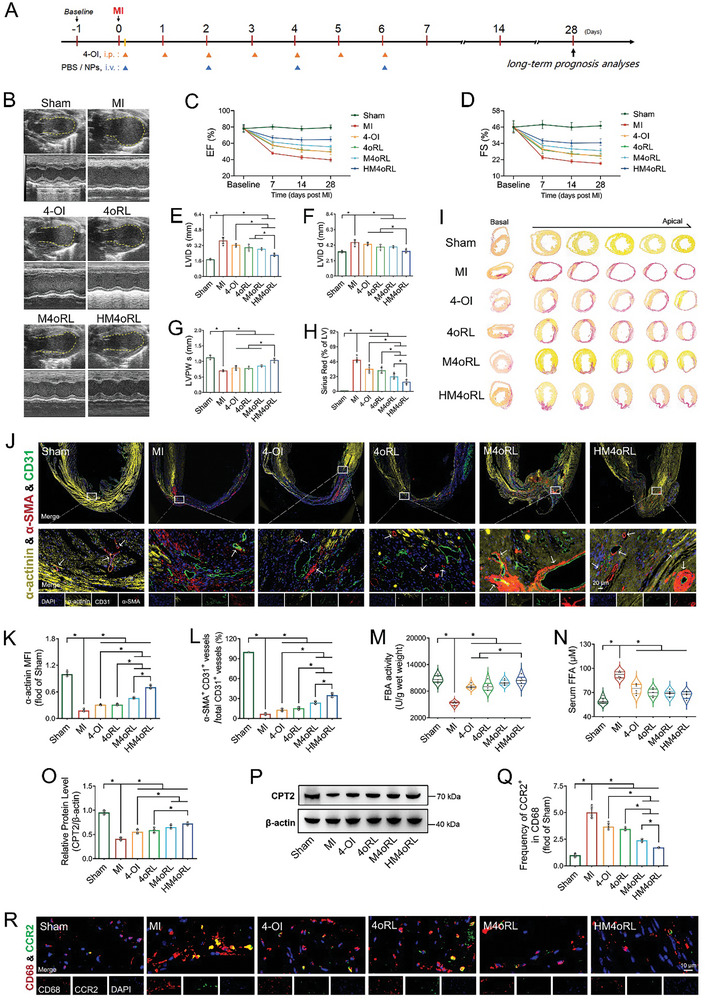
HM4oRL enhances long‐term cardiac function after MI. A) Schematic illustration of the experimental timelines for long‐term studies of MI mice. B) Representative M‐mode echocardiographic images of each group at 4 weeks post‐MI. Left ventricle EF C) and FS D) assessed by echocardiography at the indicated time points before and post MI for each group. n = 5. Quantification analysis for LVIDs E), LVIDd F), and LVPWs G) based on the M‐mode echocardiographic images at 4 weeks post‐MI. n = 5. Representative Sirius red staining images of myocardial sections I) and quantitative analysis of myocardial fibrosis size H) at 4 weeks post‐MI. n = 5. J) Representative immunofluorescence images of heart sections triple‐stained with fluorescein‐labeled α‐actinin (yellow), α‐SMA (red), CD31(green) and nuclei (blue) at 4 weeks post‐MI. Quantification of cardiomyocyte density (α‐actinin‐positive) K) and the number of vessels (both α‐SMA and CD31 positive) L) in the infarct zone. n = 5. M) Levels of FBA activity in injured myocardium after different treatments. n = 6. N) Levels of serum FFA after different treatments. n = 6. O‐P) Western blotting analysis of CPT2 protein content alterations in injured myocardium after different treatments. n = 4. R) Representative double‐immunofluorescence staining for CCR2 and CD68 in infarct tissue at 4 weeks post‐MI, and Q) quantification of CCR2^+^ macrophages for each group by ImageJ software. Green, CCR2; red, CD68; blue, DAPI. n = 5. Data are presented as mean ± SD. Statistical methods: One‐way ANOVA with Tukey's post‐test (E‐H, K‐O, Q). In all panels, * indicates *p* < 0.05.

To clarify the link between the anti‐pyroptotic and immune‐inflammatory regulatory activity of HM4oRL NPs and long‐term prognostic outcomes, post‐MI cardiac function was monitored by echocardiography (**Figure** **9**A). Following AMI, sustained reductions in LVEF and left ventricular fractional shortening (FS) (Figure 9B‐D). By day 28, significant increases in both left ventricular internal diameter at end‐systole (LVIDs) (Figure 9E) and left ventricular internal diameter at end‐diastole (LVIDd) were observed (Figure 9F), together with a marked reduction in the left ventricular posterior wall at end‐systole (LVPWs) (Figure 9G). Sirius red staining additionally confirmed that the extent of myocardial fibrosis area in the MI group was as high as 48.83 ± 4.37% (Figure 9H‐I). These findings are consistent with post‐MI adverse ventricular remodeling. HM4oRL NP treatment significantly enhanced cardiac function in these mice, effectively suppressing progressive adverse ventricular remodeling after MI (Figure 9C‐I).

Long‐term therapeutic benefits of HM4oRL treatment were also confirmed through analyses of cardiomyocytes, metabolic activity, and inflammatory cells. Immunofluorescence revealed that the infarcted area exhibited pronounced cardiomyocyte loss (α‐actinin staining), together with fewer structurally mature vessels (CD31/α‐SMA double‐positive vessels) (Figure 9J‐L). In the HM4oRL NP‐treated group, however, significantly greater cardiomyocyte levels and a greater number of structurally mature vessels were noted. Intriguingly, rather than remaining elevated in the infarcted myocardium, FBA activity levels were significantly reduced (Figure 9M), whereas serum FFA levels remained elevated (Figure 9N), and CPT2 protein levels in the infarcted heart were strikingly reduced (Figure 9O‐P). These results may be indicative of a metabolic shift in the infarcted myocardial tissue from the compensatory to decompensatory phase. HM4oRL NPs were able to significantly attenuate the reduction in post‐MI FBA activity while enhancing CPT2 expression and preserving lower serum FFA levels (Figure 9M‐P). Immunohistochemical staining for CCR2^+^ macrophages revealed that the frequency of these cells decreased markedly after HM4oRL NP treatment (Figure 9Q‐R). These results support the ability of HM4oRL NPs to readily rescue cardiomyocyte damage and to foster enhanced cardiac repair through the regulation of immune‐inflammatory responses, providing an extremely effective cardioprotective approach to MI treatment.

As treating some patients within the optimal 6‐h post‐AMI window may be infeasible, the effects of HM4oRL salvage therapy were also assessed through the delayed administration of these NPs. Even when they were administered in two injections 48 h post‐MI, HM4oRL NPs still presented with striking cardioprotective activity (Figure S20, Supporting Information). Additionally, mice that received delayed treatment (two injections administered 48 h post‐MI) showed a reduction in EF and FS to 52.81 ± 3.26% and 26.84 ± 2.16%, respectively, when compared to the early treatment group (four injections). However, these cardiac function parameters remained markedly higher than those observed in the MI group, which EF and FS were 38.63 ± 3.56% and 18.59 ± 1.98%, respectively (Figure S21, Supporting Information). These findings further demonstrated the cardioprotective effects of HM4oRL NPs and highlighted their potential for clinical translation.

### 2.12 Evaluation of the Biosafety Profile of HM4oRL NPs

Lastly, HM4oRL NPs were evaluated for potential toxicity by administering them to mice every other day for two weeks at three times the therapeutic dose level, monitoring changes in body weight and serum biochemical indexes (Figure S22A, Supporting Information). No significant differences were observed in body weight (Figure S22B, Supporting Information) or serum markers of liver, kidney, or hematological toxicity between the HM4oRL and control groups (Figure S22C‐H, Supporting Information). As the surfaces of HM4oRL NPs contain inflammatory receptors, inflammatory cytokine levels were additionally examined in these mice, revealing that HM4oRL treatment did not induce any significant systemic inflammation (Figure S22I,J, Supporting Information). Moreover, hematoxylin and eosin staining revealed no apparent histopathological changes in the major organs (heart, liver, spleen, lung, and kidney) following HM4oRL NP administration (Figure S22K, Supporting Information).

## 3 Discussion

In this study, we successfully employed an evidence‐based approach to design novel neutrophil‐macrophage hybrid membrane‐coated, multifunctional TA‐Mn film‐modified nanocarriers suitable for targeted AMI MR imaging and drug delivery. According to our best knowledge, our study has significant novelties: (1) Injured myocardium targeting. The engineered succinate‐activated neutrophil‐macrophage HM coating enabling these HM4oRL NPs to preferentially target infarcted sites during the early stages of AMI, as confirmed through fluorescence imaging and corresponding TTC staining. The responsive properties of these NPs further facilitated the selective release of payloads specifically within the infarcted myocardium, effectively bolstering local 4‐OI bioavailability and T1‐weighted contrast enhancement. (2) MR imaging‐based theranostic nanoplatform. The TA‐Mn modification of these coated liposomes afforded greater diagnostic utility, as the resultant HM4oRL NPs presented with a good r1 relaxivity of 4.95 Mm^−1^s^−1^, making them well suited for use as tools for in vivo cardiac T1‐weighted MR imaging. (3) Cardioprotection is achieved through robust anti‐pyroptotic properties, metabolic regulation, and the reprogramming of cardiac immune‐inflammatory pathways. Targeted NP‐mediated 4‐OI delivery to the injured myocardial tissue was sufficient to protect cardiomyocytes against pyroptotic induction, thereby disrupting the cascading immune‐inflammatory responses that arise following AMI. Moreover, these HM4oRL NPs preserved cardiac metabolic homeostasis through the activation of AMPK signaling activity, contributing to the reprogramming CRM‐mediated inflammatory pathways. Through these mechanisms, HM4oRL NPs were able to abrogate post‐AMI myocardial injury while promoting better infarct healing and preventing adverse ventricular remodeling. (Scheme 1B).

Cardiac‐specific ligands including antibodies, proteins, peptides, and small molecules have previously been used to produce modified nanocarriers that can preferentially accumulate in infarcted areas.^[^ ^46^ ^]^ The complex, dynamic microenvironmental and pathophysiological conditions associated with MI, however, remain a persistent barrier to the ability of these approaches to effectively target the damaged myocardium. Here, a bespoke biomimetic targeting strategy was designed with the aim of achieving superior targeting activity in AMI. Some overlap was noted when comparing the top 20 DEGs in succinate‐activated neutrophils and the top DEGs identified in single‐cell transcriptomic analyses of myocardium‐infiltrating neutrophils, including Ccl3, Cxcl2, and Icam1.^[^ ^47^ ^]^ Cardiac Ccl3^hi^ neutrophils have been established as a subset that readily infiltrates the myocardium at early time points following myocardial injury. Based on these results, the in vitro succinate‐mediated activation of neutrophils used in this study may at least partially recapitulate the microenvironmental features of MI. Over 20 integrin types have been identified to date, with β1 subfamily integrins primarily facilitating extracellular matrix adhesion, while α5 interacts predominantly with fibronectin.^[^ ^48^ ^]^ Interestingly, fibronectin expression reported increases significantly within 3 h post‐MI in the infarcted region, and its expression peaks at earlier time points than many other extracellular matrix network‐related proteins.^[^ ^49^ ^]^ Here, HM4oRL NPs were found to effectively preserve the expression of membrane‐derived integrins including α5, β1, and αX, thus enhancing the ability of these NPs to adhere to the extracellular matrix in the damaged myocardium. Relative to unmodified M4oRL NPs, these HM4oRL NPs thus presented with superior retention and aggregation in the injured myocardium after AMI. HM modification thus imbues these NPs with the ability to more readily target infarcted tissues, making them ideal tools for cardioprotective treatment in an acute care setting.

MPN‐based NPs and coatings have recently emerged as promising tools for cancer diagnosis and treatment.^[^ ^50^ ^]^ This study is, to the best of our knowledge, the first in which an MPN‐modified nanoplatform was successfully used to aid in the diagnosis and treatment of AMI. Combined FT‐IR, XPS, and TEM analyses suggest that the utilized TA‐Mn complex may be able to encapsulate 4oRL without affecting the morphological structure of this cargo. Formed from coordination bonds between TA and Mn, this TA‐Mn film can disassemble rapidly when exposed to a low pH as a result of hydroxyl group protonation.^[^ ^51^ ^]^ As HM4oRL NPs were able to preferentially target injured myocardial tissue and to release their cargo in response to local conditions, this facilitated a specific accumulation of Mn^2+^ in the infarcted myocardium, providing enhanced contrast for these infarcted regions when imaged with a 9.4 T Micro MR scanner. MPN encapsulation also imbued these NPs with superior ROS scavenging activity, as confirmed through ABTS and in vitro assays. This enhanced ROS clearance, coupled with the ability of 4‐Ol to directly inhibit the activation of NLRP3,^[^ ^17^ ^]^ led to the effective HM4oRL‐mediated suppression of post‐MI NLRP3/Caspase‐1/GSDMD‐mediated cardiomyocyte pyroptosis. Relative to 4oRL alone, HM4oRL NPs presented with better pyroptosis‐inhibiting activity together with greater cytoprotective benefits for hypoxia‐exposed H9c2 cells. This suggests a potential synergistic effect between the ROS‐scavenging properties of the MPN and the loaded 4‐OI within HM4oRL. As a result, the superior pyroptosis‐regulating activity of these HM4oRL NPs serves as a robust tool for the disruption of cascading inflammatory signaling following AMI, thereby more favorably shaping consequent immune response induction.

The biodistribution and clearance of nanoparticles are intricately influenced by physicochemical properties such as particle size, surface charge, and shape. Regarding non‐target organ biodistribution, HM4oRL NPs primarily accumulate in the liver, aligning with distribution patterns commonly observed in other nanoformulations.^[^ ^52^ ^]^ This hepatic accumulation occurs because these nanoparticles are readily taken up by the reticuloendothelial system (RES). While immune cell membrane‐based biomimetic nanomedicines demonstrate potential to evade immune surveillance during systemic circulation, our findings indicate a decreasing trend in the hepatic accumulation of HM4oRL NPs. This reduction in liver uptake may be owing to the specific composition of the hybrid membrane used in these nanoparticles. While some accumulation in non‐target organs is inevitable, our study nonetheless confirms the favorable biosafety of HM4oRL NPs. Additionally, the particle size of HM4oRL NPs, which exceeds 100 nm, combined with IVIS results, suggests that the liver may serve as primary clearance pathway for these nanoparticles.^[^ ^53^ ^]^ Future refinements, such as adjusting the ratio of hybrid cell membranes, reducing the size of the nanoparticles, would be explored to minimize nonspecific uptake by the RES and optimizing the targeting of the nanoparticles to the MI lesion.

Several reports have stressed the relevance of metabolic activity as a crucial nexus between cardiac and immune cells, as metabolic disorders can give rise to the accumulation of abnormal levels of metabolites that can impact immune cell functionality.^[^ ^10^ ^]^ Metabolic dysregulation emerged early in the pathogenesis of MI and coincided with extensive pro‐inflammatory CCR2^+^ monocyte/macrophage infiltration in the present study. Integrated omics analyses revealed that this metabolic dysregulation was characterized at least in part by abnormal increases in glycolytic activity, together with impaired TAC and FA β‐oxidation activity. Strikingly, this metabolic profile closely mirrors the metabolic phenotypes exhibited by pro‐inflammatory macrophages.^[^ ^54^ ^]^ These inflammatory immune cells rely more heavily on high levels of glycolytic flux to fuel their effector functions.^[^ ^11^ ^]^ Simultaneously, during the acute MI phase, targeted HM4oRL NP‐mediated 4‐Ol delivery to the heart markedly suppressed FBA activity. This inhibition resulted in a marked reduction in specific glycolytic intermediates, including Fructose‐6P and glyceraldehyde‐3‐phosphate, which would be particularly unfavorable for pro‐inflammatory macrophage populations. Consistently, HM4oRL treatment significantly reduced CD64^int^Ly6C^high^CCR2^high^CX3CR1^low^ inflammatory monocyte/macrophage accumulation in the injured myocardium. These data suggest that treatment with HM4oRL markedly suppressed the activity of pro‐inflammatory monocytes/macrophages within the injured myocardium during the acute MI phase, potentially owing to the ability of these NPs to suppress pyroptosis and modulate metabolic activity.

Many factors influence AMPK pathway signaling, and AMP can activate this pathway via multiple complementary mechanisms.^[^ ^55^ ^]^ In metabolomics analyses, AMP levels rose significantly upon HM4oRL treatment consistent with potential AMPK pathway activation. AMPK activation in infarcted cardiac tissue has recently been shown to play a role in the maintenance of cardiac metabolic homeostasis after MI.^[^ ^56^ ^]^ Here, HM4oRL was found to markedly increase AMPK phosphorylation and to improve the expression of key metabolic pathway‐related genes in the post‐MI infarcted heart, including those linked to FA β‐oxidation, TAC, and oxidative phosphorylation. HM4oRL also reversed the reduction in CPT2 expression, supporting the ability of these NPs to maintain enhanced β‐oxidation within the infarcted myocardium. HM4oRL thus appears to activate AMPK and to preserve post‐MI cardiac metabolic homeostasis, thereby helping to establish a microenvironment that is likely more conducive to reparative monocyte/macrophage activity.

Cardiac Ly6C^low^CCR2^low^ monocytes/macrophages are central coordinators of reparative activity after cardiac injury through their ability to alleviate inflammation, promote angiogenesis, and modulate collagen deposition. Ly6C^low^CCR2^low^ monocyte/macrophage abundance and VEGF secretion increased following HM4oRL administration, leading to significantly enhanced post‐MI angiogenesis. CX3CR1^+^ CRMs can suppress pro‐inflammatory monocyte recruitment and expedite the resolution of inflammation.^[^ ^42^ ^]^ Consistently, HM4oRL NPs significantly increased the frequency of MHC‐II^low^CX3CR1^high^ CRMs among Ly6C^low^CCR2^low^ monocytes/macrophages in AMI model mice. During the cardiac repair phase, markedly reduced CCR2^+^ macrophage counts were evident in HM4oRL‐treated mice, further underscoring the ability of these NPs to resolve inflammation. Strikingly, HM4oRL treatment afforded long‐term benefits following AMI, including reductions in myocardial fibrosis and pathological ventricular dilation, together with enhanced cardiac function. These superior therapeutic outcomes are attributable to the meticulously designed cascading cardioprotective effects of these NPs afforded by their ability to interact with multiple targets. In addition to rapidly rescuing cardiomyocytes, these particles can also reprogram the metabolic‐immune microenvironment to readily prevent the onset or progression of post‐AMI adverse ventricular remodeling.

Here, HM4oRL NPs were administered intravenously given the simplicity and clinical feasibility of this dosing strategy. Under delayed treatment conditions, the myocardial fibrosis area increased from 14.83 ± 3.19% in the immediate treatment group to 24.96 ± 3.43%. However, this value remained significantly lower than the value (50.39 ± 4.19%) observed in the PBS‐treated group, underscoring the robust cardioprotective efficacy of HM4oRL NPs. Adult mammalian cardiomyocytes exhibit a limited regenerative capacity, and even a brief period of ischemia, as short as 15 min, can induce irreversible myocyte injury.^[^ ^57^ ^]^ This continuous damage triggers the release of damage‐associated molecular patterns (DAMPs), which strongly activate immune‐inflammatory responses, further exacerbating tissue injury and resulting in maladaptive healing and remodeling. These findings partially support the hypothesis that timely intervention following AMI not only mitigates acute myocardial damage but also modulates the subsequent inflammatory response, thereby influencing the long‐term outcomes of AMI. In patients with impaired endogenous cardiac repair activity, including individuals with chronic kidney disease and diabetes, the sustained delivery of therapeutic agents using cardiac patches or similar approaches may be necessary to achieve beneficial clinical outcomes.^[^ ^58^ ^]^ Future investigations could focus on optimizing the patch material, size, and drug release kinetics to improve myocardial uptake and ensure sustained therapeutic action at the injury site. Furthermore, preclinical models should be used to assess the safety, biocompatibility, and long‐term effects of this delivery approach, in comparison with traditional approaches like intravenous administration, thus establishing a robust foundation for potential clinical translation.

This study is subject to some limitations. First, additional experimental validation with preclinical models of AMI, which entail multi‐site inflammation and an adequate sample size, will be necessary to further validate both the specificity of myocardial targeting and the cardioprotective effects. Emerging evidence suggests a frequent coexistence and bidirectional interaction between HF and liver disease.^[^ ^59^ ^]^ Building upon these insights, we propose to construct a multi‐organ inflammation model by leveraging LPS to induce hepatic inflammation in a setting of MI. This model, combined with whole‐body fluorescence imaging and detailed histological analysis, will enable a comprehensive investigation of the myocardial targeting specificity of HM4oRL and its potential off‐target effects, advancing the clinical translatability of HM4oRL. Given the potential for the off‐target effects of HM4oRL treatment, we will also work to further optimize this nanoplatform through the adjustment of hybrid membrane ratios or the use of a multi‐level targeting strategy with superior myocardial specificity to mitigate non‐specific tissue effects. Second, the utility of HM4oRL NPs was only tested in the context of 9.4T high‐field‐strength MR imaging. As 3T MRI systems are commonly used in clinical settings, additional tests of our NPs with these standard clinical MRI systems are warranted, as is their combination with various biomarker candidates, thereby providing a more effective approach to AMI diagnosis.^[^ ^60^ ^]^ Third, this study has offered limited insights into the potential synergistic effects of the active compounds within HM4oRL NPs for MI treatment. Future research should employ rigorous experimental designs to quantify these effects, using tools like CompuSyn software and the Chou‐Talalay method to calculate synergy indices.^[^ ^61^ ^]^ Both in vivo and in vitro experiments, combined with multi‐omics analysis, will be essential for further elucidating these synergistic effects. Lastly, we acknowledge the importance of sex‐based differences in MI treatment, as substantial evidence indicates that biological differences, such as hormonal influences and genetic factors, can affect MI pathophysiology and treatment response.^[^ ^62^ ^]^ Future studies should systematically include both male and female subjects to better understand how sex may modulate the effects of HM4oRL NPs and enhance the generalizability of our findings.

## 4 Conclusion

In summary, a novel cardiac‐targeted theranostic nanoplatform was herein developed for the multi‐dimensional regulation of AMI‐associated inflammation. The MPN modification of these NPs additionally makes them ideal as an advanced nano‐delivery platform for the treatment of other forms of inflammatory disease. Our systematic experimental efforts highlighted the ability of HM4oRL to synergistically influence many different facets of the cardiac immune response to ultimately protect against AMI‐associated cardiac injury and adverse ventricular remodeling. Together, HM4oRL NPs thus hold immense promise as a cardioprotective therapeutic tool for the targeted mitigation of immune inflammation‐associated myocardial injury, providing an unprecedented level of control with the potential for future clinical application.

## 5 Experimental Section

For a full description of the materials used for this study, liposome preparation, MPN‐coated liposome NP preparation, bone marrow‐derived neutrophil extraction and activation, hybrid membrane‐coated NP preparation, NP characterization, cell culture, cell treatment, MI modeling and treatment, and associated references, see the Supporting Information.

### Animal Sources

The animal experiments described in the study were conducted in compliance with the National Institutes of Health for the care and use of laboratory animals. Male C57BL/6 mice, aged 6–8 weeks, were procured from GemPharmatech (Nanjing, China) and used under protocols approved by the Ethical Review of Animal Experiments at Nanjing Jinling Hospital (2019JLHGKJDWLS‐050).

### Statistical Analysis

Statistical analyses were conducted using IBM SPSS Statistics (version 26.0.0.0). Quantitative data with a normal distribution are presented as the mean ± standard deviation (SD). The normality of the data was evaluated using the Shapiro‐Wilk test. Comparisons between two groups were performed using Independent Samples Tests, including a two‐tailed unpaired Student’ s *t*‐test or the nonparametric Mann‐Whitney *U* test. Comparisons among three or more independent groups were conducted using one‐way analysis of variance (ANOVA), followed by Tukey's post hoc test when the assumptions of normality and homogeneity of variance were satisfied. In cases where the assumption of homogeneity of variance was not met, Tamhane's post hoc test was applied. A *p*‐value < 0.05 was considered statistically significant. Data visualization was performed using GraphPad Prism (version 8.0).

## Conflict of Interest

The authors declare no conflict of interest.

## Author Contributions

T.Z. and J.S. contributed equally to this article. T.Z., G.L., and L.Z. designed the research. T.Z., H.W., L.Z., and Y.Z. carried out the experiments. J.S. and H.W. performed data analysis. Z.W. and S.W. participated in part of the experiments. J.L. provided suggestions on animal model constructions. T.Z. wrote the original manuscript. J.S. and Z.W. revised the manuscript. G.L. and L.Z. had supervision of all study. L.Z. conceived the project. All of the authors have read and approved the final manuscript.

## Supporting information



Supporting Information

## Data Availability

The data that support the findings of this study are available on request from the corresponding author.
