# Supplementary material for: Injured Myocardium‐Targeted Theranostic Nanoplatform for Multi‐Dimensional Immune‐Inflammation Regulation in Acute Myocardial Infarction
Source: Adv Sci (Weinh). 2025 Jan 21;12(10):2414740. doi: 10.1002/advs.202414740 (PMC11904987; doi:10.1002/advs.202414740)
Supplement: Supplementary file 1 — Supporting Information [file ADVS-12-2414740-s001.docx]

**Injured Myocardium-Targeted Theranostic Nanoplatform for Multi-Dimensional Immune-Inflammation Regulation in Acute Myocardial Infarction**

Tao Zheng, Jie Sheng, Zhiyue Wang, Haoguang Wu, Linlin Zhang, Sheng Wang, Jianhua Li, Yunming Zhang, Guangming Lu, and Longjiang Zhang*

T. Zheng, J. Sheng, Z. Wang, H. Wu, L. Zhang, Y. Zhang, G. Lu, L. Zhang

Department of Radiology

Nanjing Jinling Hospital

Affiliated Hospital of Medical School

Nanjing University

305 East Zhongshan Road, Nanjing 210002, China

E-mail: kevinzhlj@nju.edu.cn

S. Wang

Department of Radiology

Nanjing Jinling Hospital

Nanjing Medical University

305 East Zhongshan Road, Nanjing 210002, China

J. Li

Department of Cardiology

Nanjing Jinling Hospital

Affiliated Hospital of Medical School

Nanjing University

305 East Zhongshan Road, Nanjing 210002, China

**Supplement-Figures**


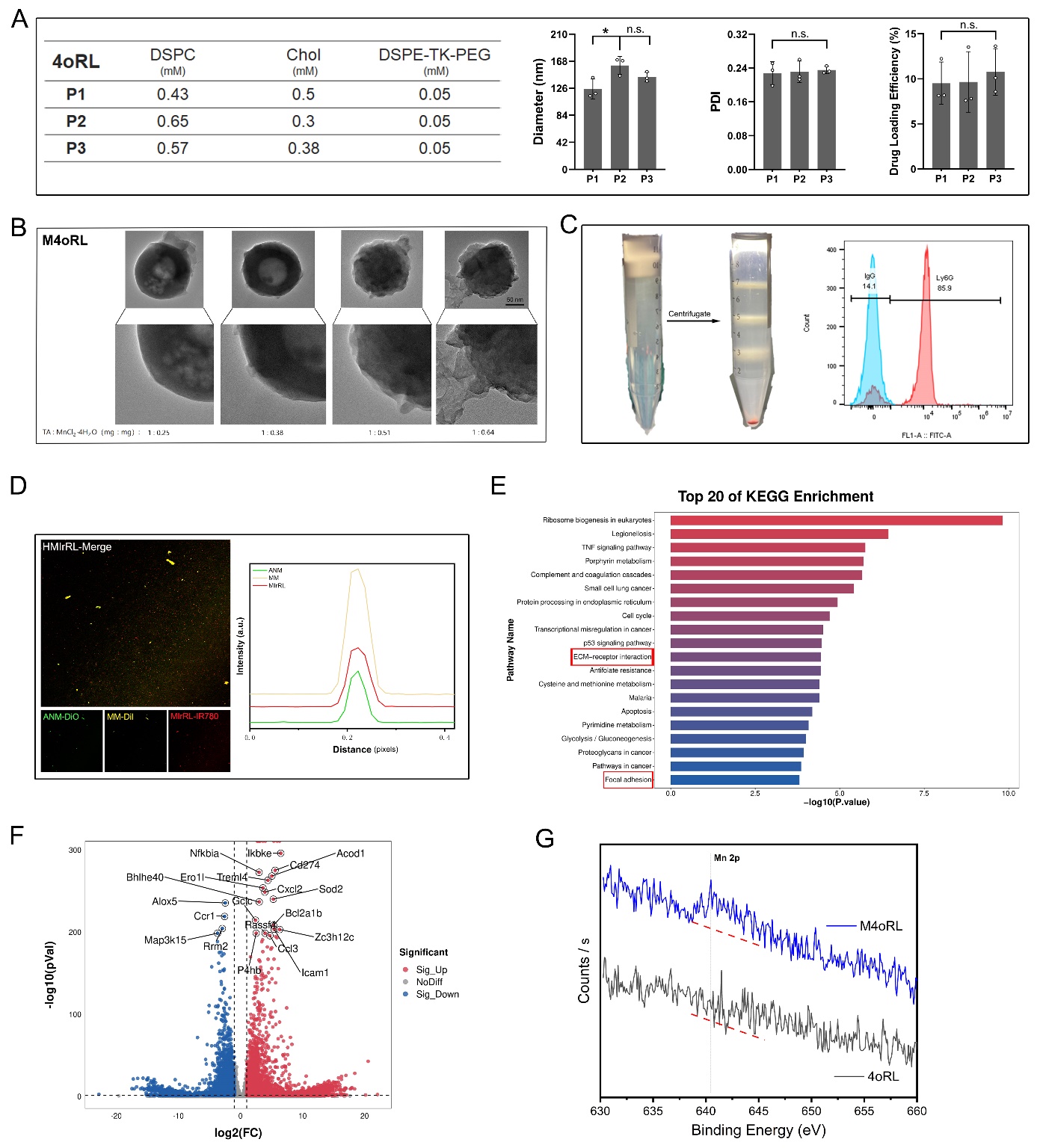
**Figure S1.** (A) Characterizations of 4oRL including diameter, PDI, and drug loading efficiency under different proportions of DSPC:Cholesterol:DSPE-TK-PEG. n=3. (B) Representative TEM images of M4oRL nanoparticles under different proportions of TA: MnCl_2_ in water. (C) Isolation of neutrophils from mouse bone marrow before and after density gradient centrifugation, followed by identification of neutrophil purity using flow cytometry. (D) Confocal laser scanning microscopy images of activated neutrophil membrane (ANM) doped with Dio (green), macrophage membrane (MM) doped with Dil (yellow), IR780-labeled MIrRL (red). (E) Top 20 Kyoto Encyclopedia of Genes and Genomes (KEGG) pathways in succinate activated neutrophils *in vitro*. n=3. (F) Volcano plot illustrating the top 20 DEGs in the transcriptomic analysis of succinate activated neutrophils *in vitro*. (G) High-resolution XPS spectra of Mn 2p in 4oRL and M4oRL nanoparticles. Data are presented as mean ± SD. Statistical methods: One-way ANOVA with Tukey’s post-test (A). In all panels, * indicates *p* < 0.05, and 'n. s.' indicates no significance.


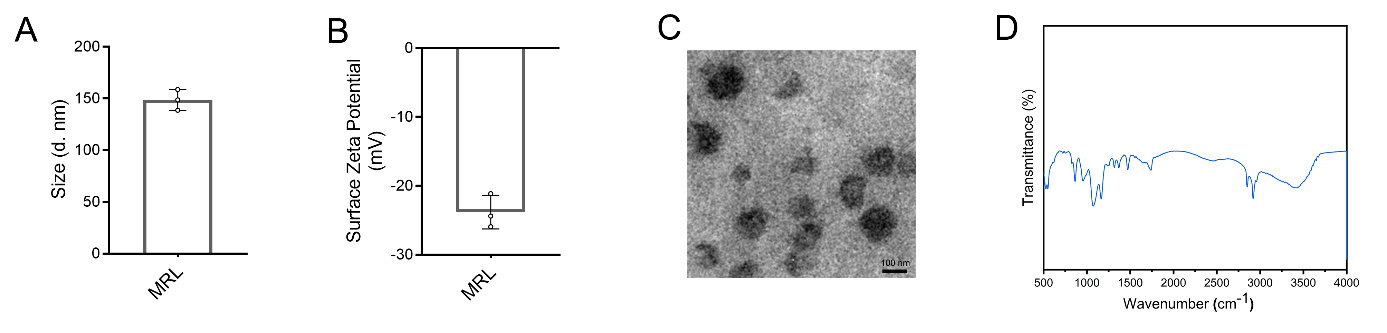


**Figure S2.** (A) Particle sizes and (B) surface Zeta potential of MRL nanoparticles. n=3. (C) Representative TEM images of MRL nanoparticles in water. (D) FTIR spectra of MRL nanoparticles. Data are presented as mean ± SD.


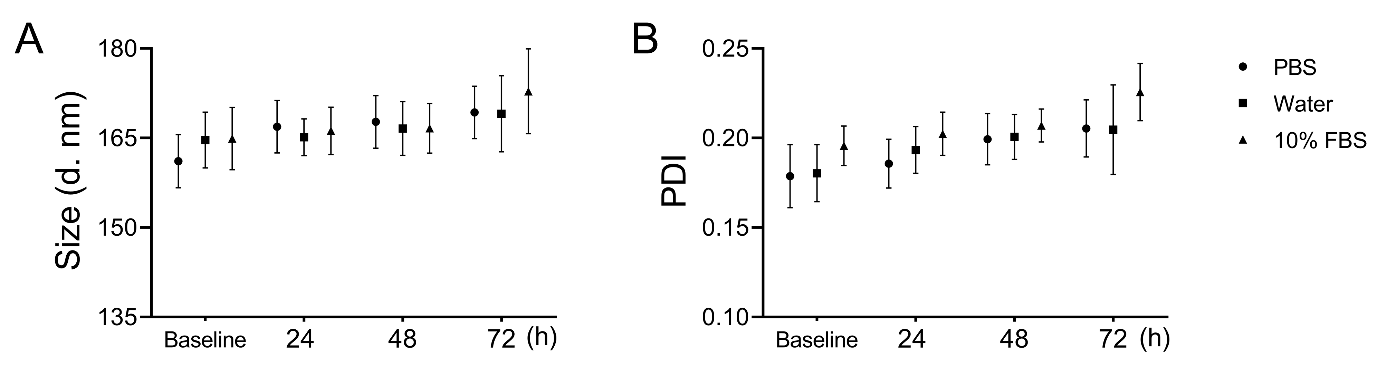


**Figure S3.** (A-B) The time-dependent colloidal stability of HM4oRL in PBS, water, and DMEM containing 10% FBS, measured by dynamic light scattering. n=3. Data are presented as mean ± SD.

**
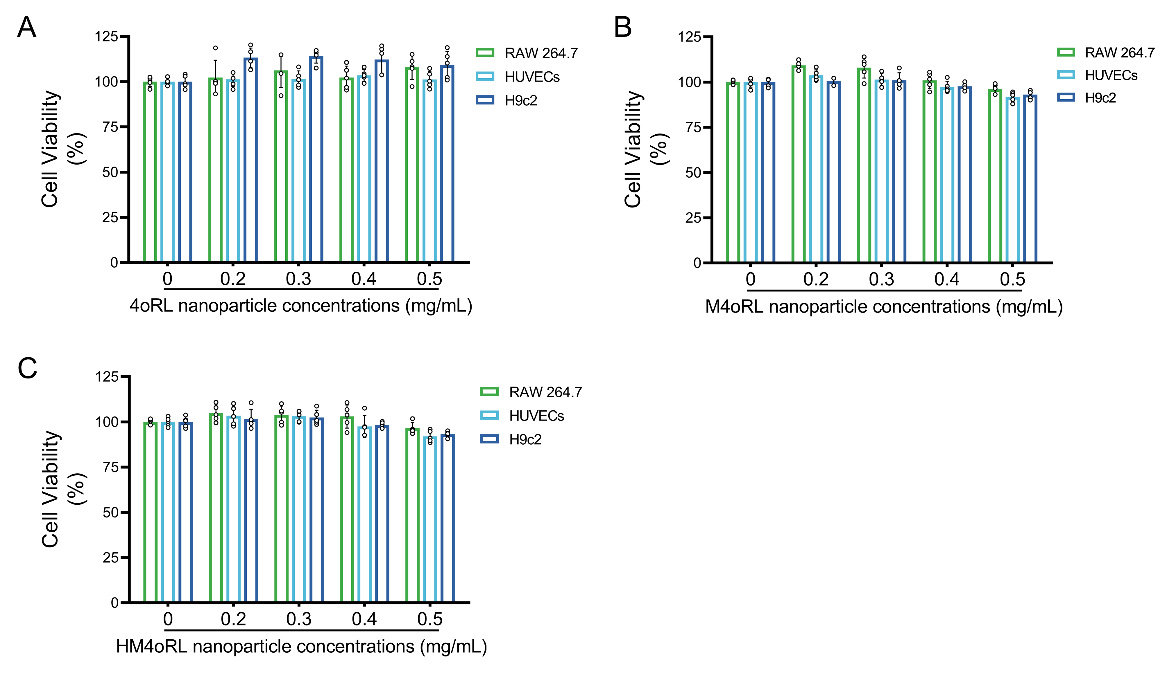
Figure S4.** Cell viability of (A) 4oRL, (B) M4oRL, and (C) HM4oRL in RAW 264.7 macrophages, HUVECs, and H9c2 cardiomyocytes after 24 hours co-incubation. n=5. Data are presented as mean ± SD.

**
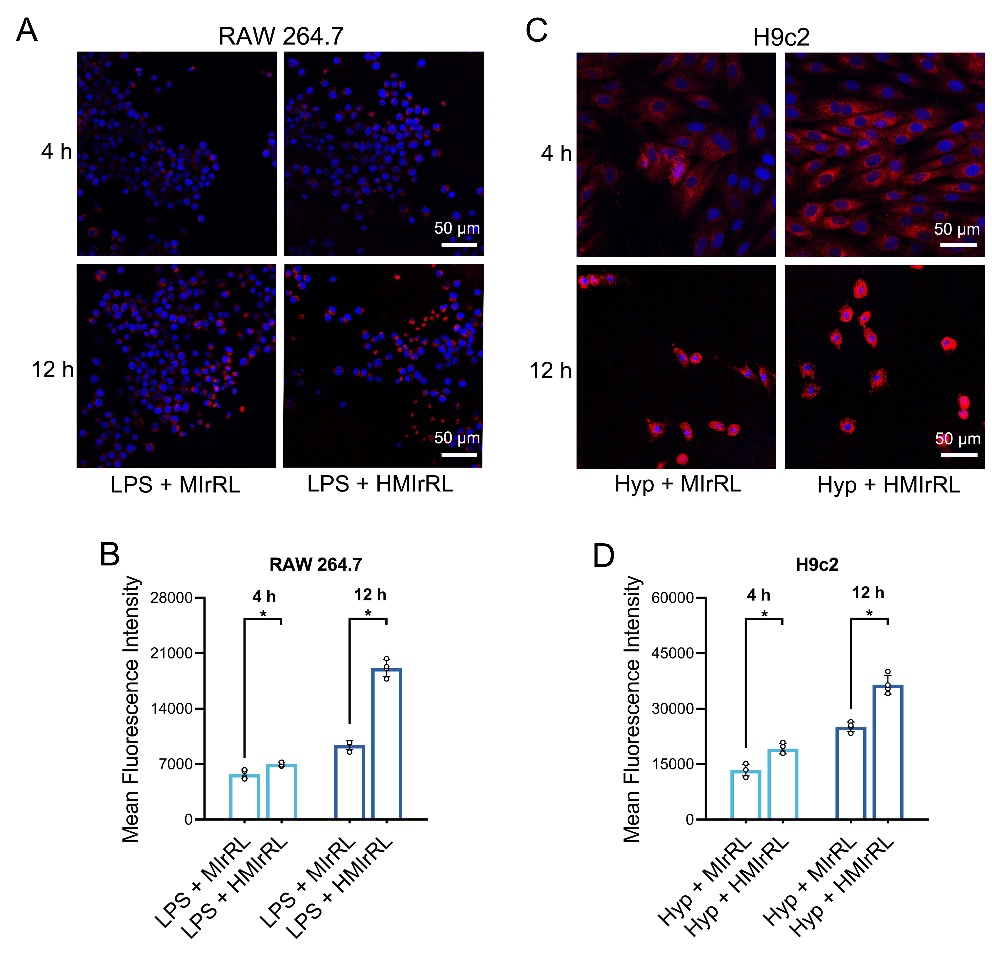
**

**Figure S5.** (A) Fluorescent images and (B) flow cytometry data of LPS-treated RAW 264.7 cells after co-incubation with MIrRL or HMIrRL for 4 or 12 hours. (C) Fluorescent images and (D) flow cytometry data of hypoxia-treated H9c2 cells after co-incubation with MIrRL or HMIrRL for 4 or 12 hours. n=4. Data are presented as mean ± SD. Statistical methods: Independent Samples Tests (B, D). In all panels, * indicates *p* < 0.05.

**
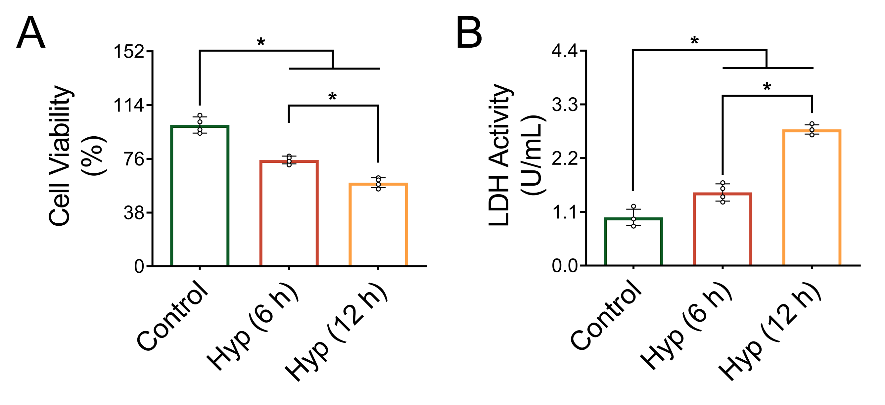
**

**Figure S6.** Cellular injury in H9c2 cells was assessed using the CCK-8 (A) and LDH assays (B) after exposure to varying durations of hypoxia. n=4. Data are presented as mean ± SD. Statistical methods: One-way ANOVA with Tukey’s post-test (A, B). In all panels, * indicates *p* < 0.05.


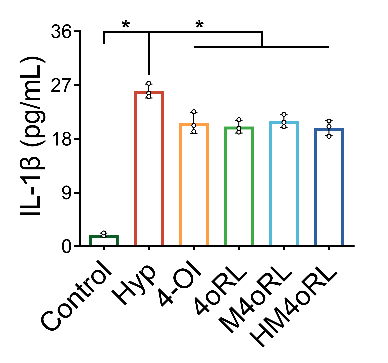


**Figure S7.** Quantification of IL-1β levels in the media of hypoxia-induced H9c2 cells. n=3. Data are presented as mean ± SD. Statistical methods: One-way ANOVA with Tukey’s post-test. In all panels, * indicates *p* < 0.05.


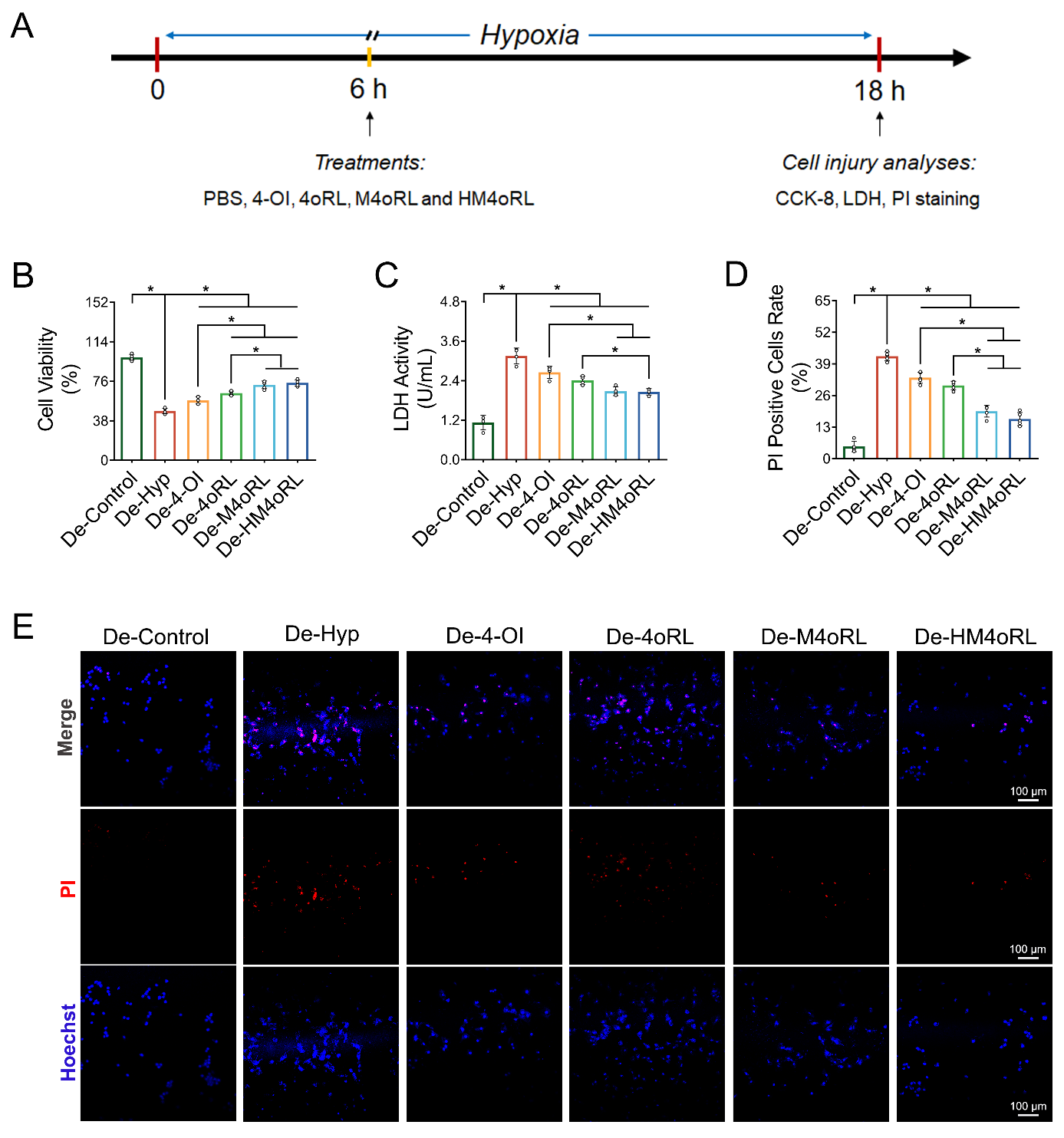
**Figure S8.** (A) Schematic illustration of prolonged hypoxia treatment and delayed drug administration *in vitro*. (B) CCK-8 assay for assessing H9c2 cell viability. n=5. (C) LDH activity measured in the media of H9c2 cells following prolonged hypoxia. n=4. (D-E) Representative images of PI-stained H9c2 cells from each group after prolonged hypoxia treatment. n=5. Data are presented as mean ± SD. Statistical methods: One-way ANOVA with Tukey’s post-test (B-D). In all panels, * indicates *p* < 0.05.


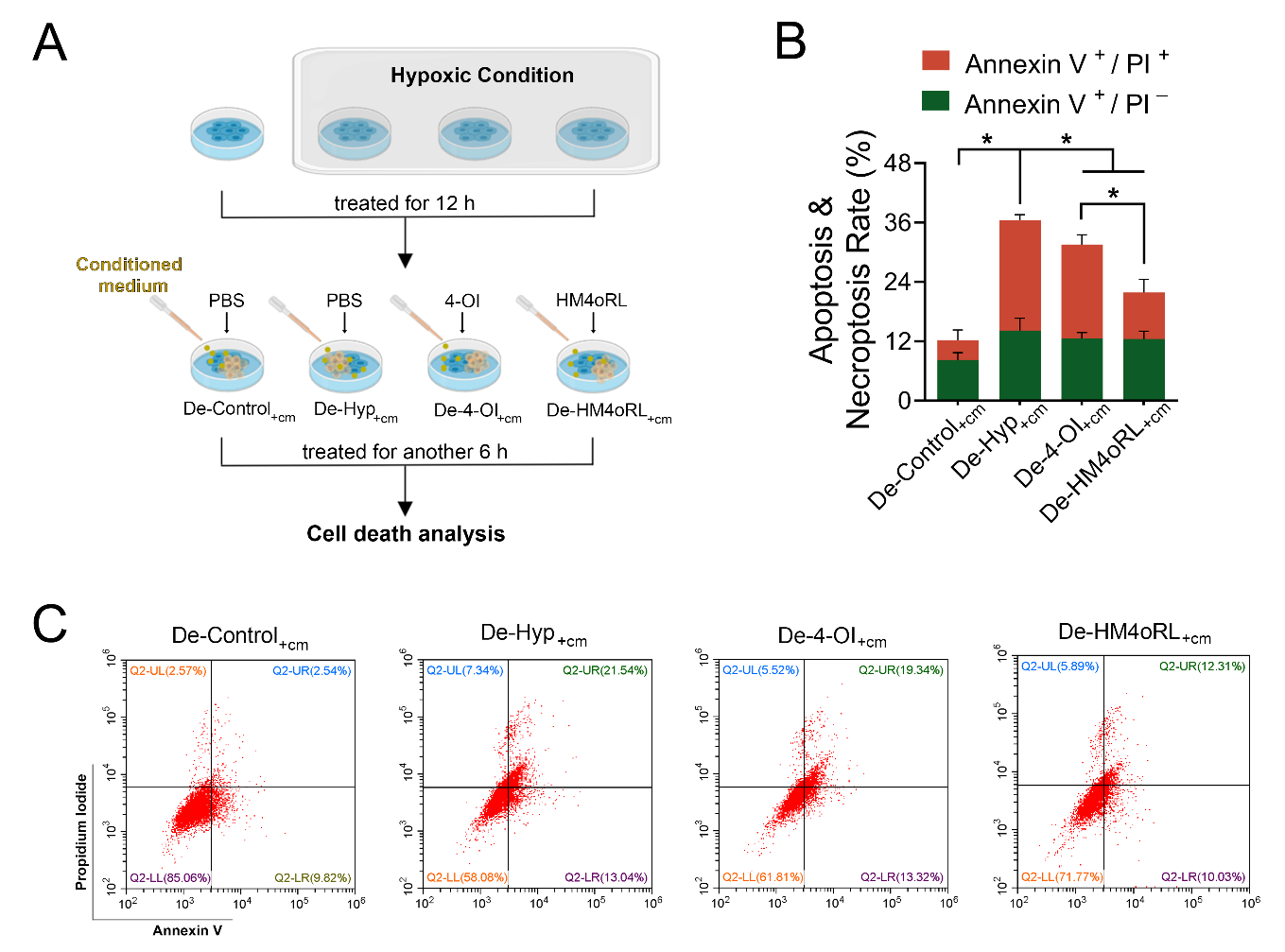
**Figure S9.** (A) Schematic illustration of prolonged hypoxia-induced H9c2 cells exposed to continuous inflammatory stimulation with conditioned medium. The conditioned medium (cm) was obtained from the supernatants collected after RAW 264.7 cells were stimulated with 200 ng/mL of LPS for 24 hours. (B-C) Cell death analysis by flow cytometry following the indicated treatments. n=4. Data are presented as mean ± SD. Statistical methods: One-way ANOVA with Tukey’s post-test. In all panels, * indicates *p* < 0.05.


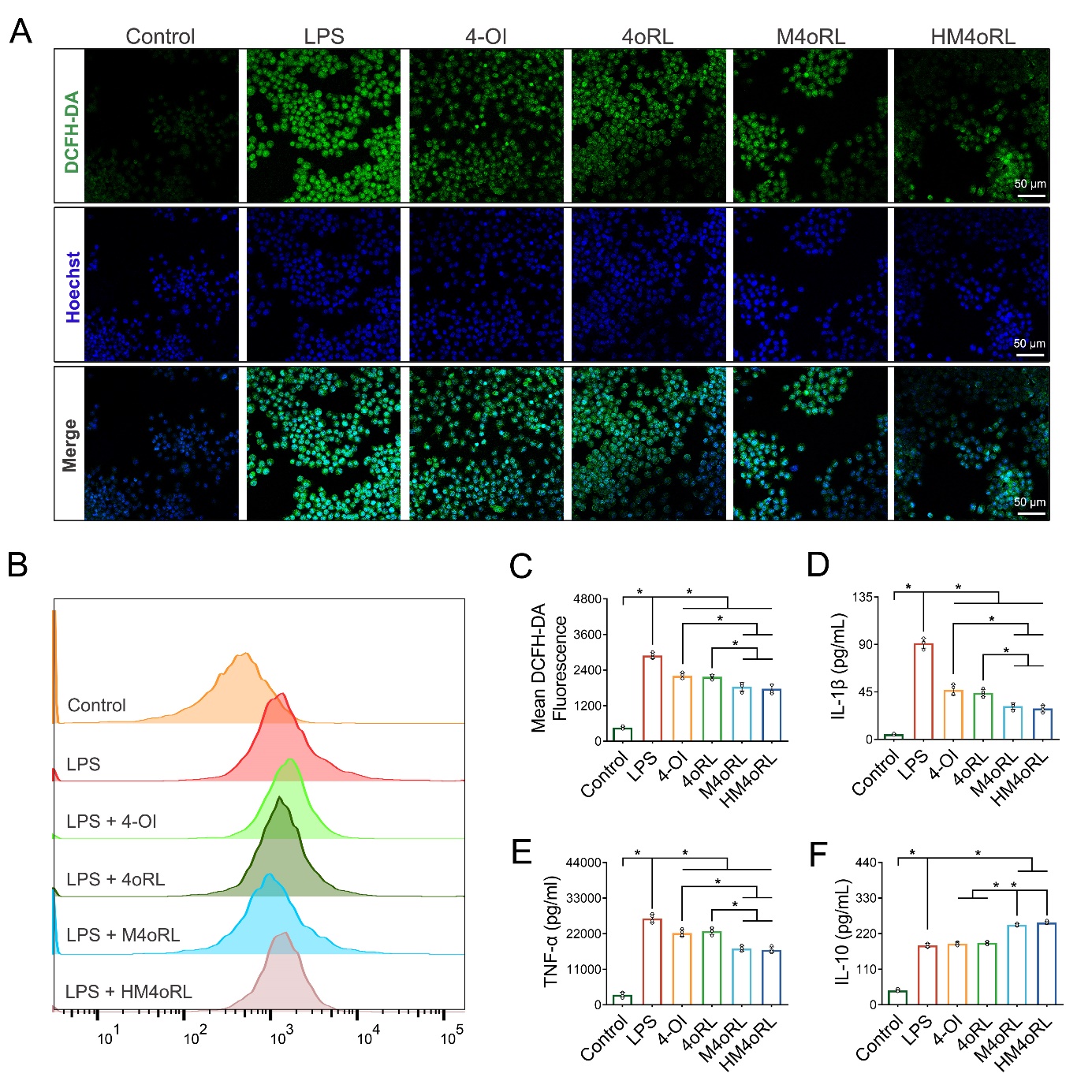


**Figure S10.** (A) Fluorescent images of intracellular ROS levels stained with DCFH-DA probe in LPS-activated RAW 264.7 cells following different treatments. Green, DCFH-DA; blue, Hoechst. (B) FlowJo analysis of ROS mean fluorescence intensity values for each group based on the flow cytometry data. n=4. (C) Corresponding quantification of ROS levels by flow cytometry. (D) IL-1β, (E) TNF-α, and (F) IL-10 levels in the media of LPS-activated RAW 264.7 cells following different treatments. n=4. Data are presented as mean ± SD. Statistical methods: One-way ANOVA with Tukey’s post-test (C-F). In all panels, * indicates *p* < 0.05.


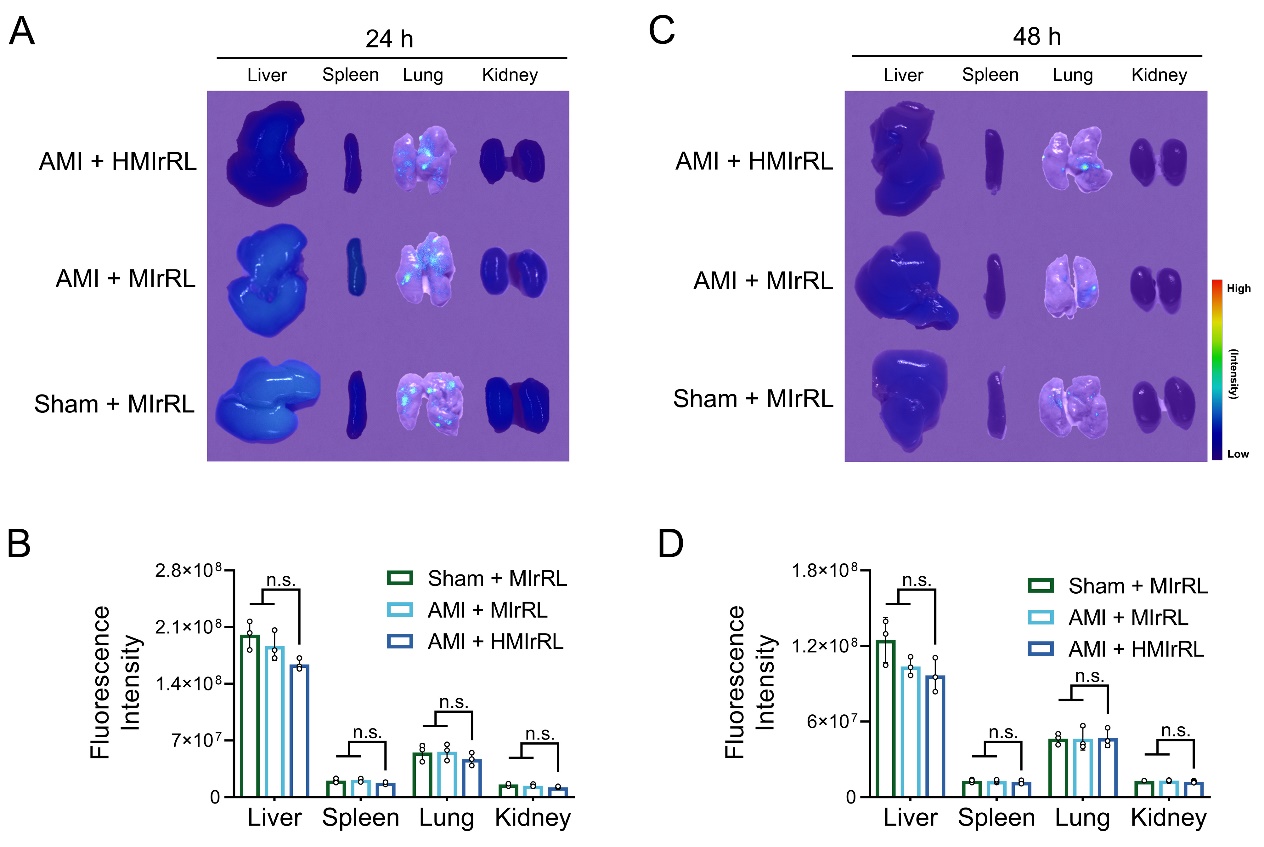
**Figure S11.** Representative *ex vivo* NIRF fluorescence images of major organs from different groups at 24 h (A, B) and 48 h (C, D) post-intravenous injection in mice, along with fluorescence intensity quantification in these organs using ImageJ software. n=3. Data are presented as mean ± SD, * indicates *p* < 0.05. Statistical methods: One-way ANOVA with Tukey’s post-test (B-D). In all panels, 'n. s.' indicates no significance.


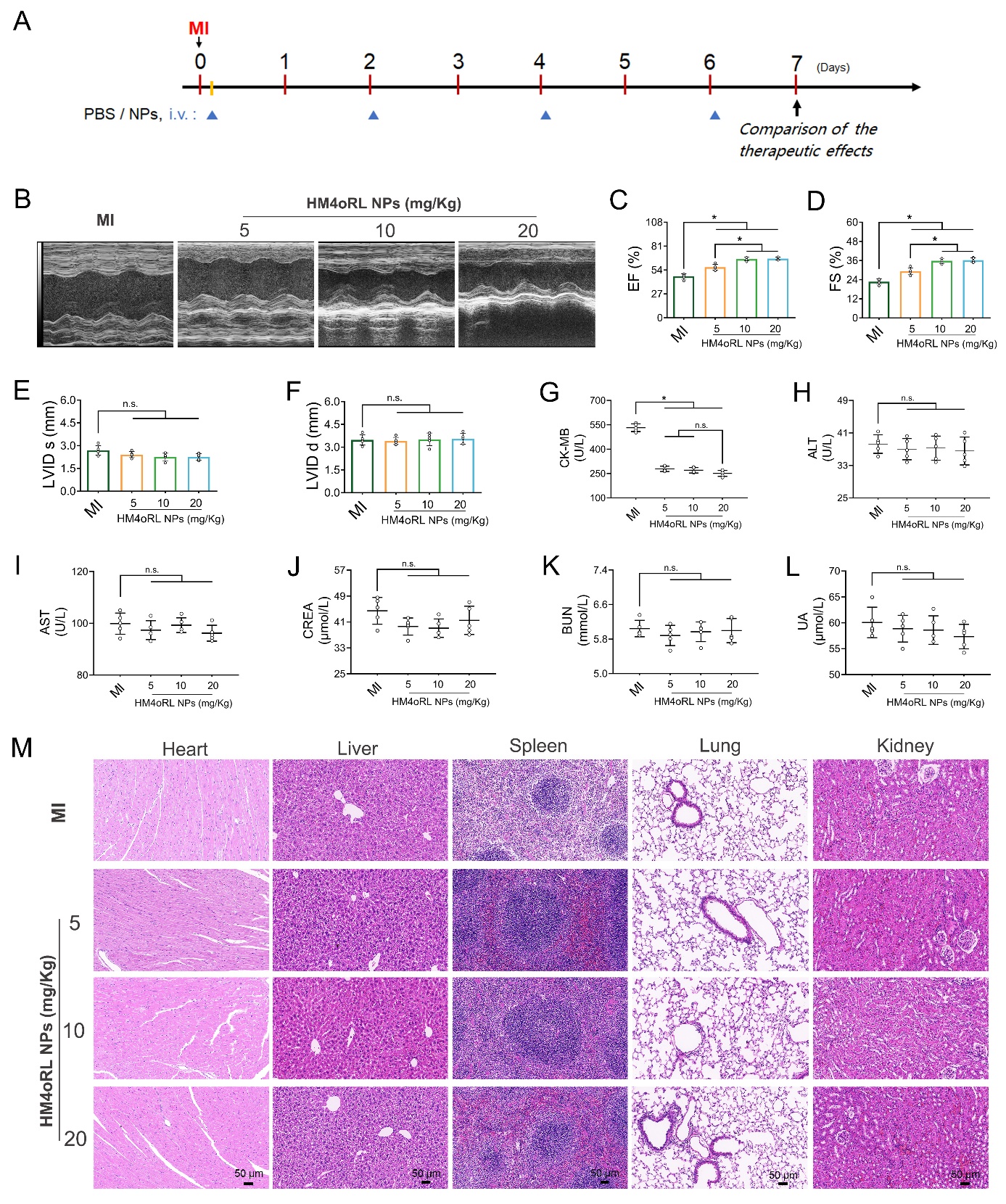


**Figure S12.** (A) Schematic illustration of *in vivo* experiment with varying doses of HM4oRL NPs. (B) Representative echocardiography images of cardiac function in different groups 7 days post-MI. (C-F) Quantification of left ventricular ejection fraction (EF), fractional shortening (FS), left ventricular internal diameter at systole (LVIDs), and diastole (LVIDd) in each group, 7 days post-MI. n=5. (G) Serum myocardial enzyme CK-MB levels after indicated treatments. n=5. (H-L) Quantitative analysis of serum hematological and biochemical parameters after indicated treatments. n=4. (M) H&E staining images of heart, lung, liver, spleen, and kidney collected from MI-operated mice after indicated treatments. Data are presented as mean ± SD. Statistical methods: One-way ANOVA with Tukey’s post-test (C-L). In all panels, * indicates *p* < 0.05, and 'n. s.' indicates no significance.


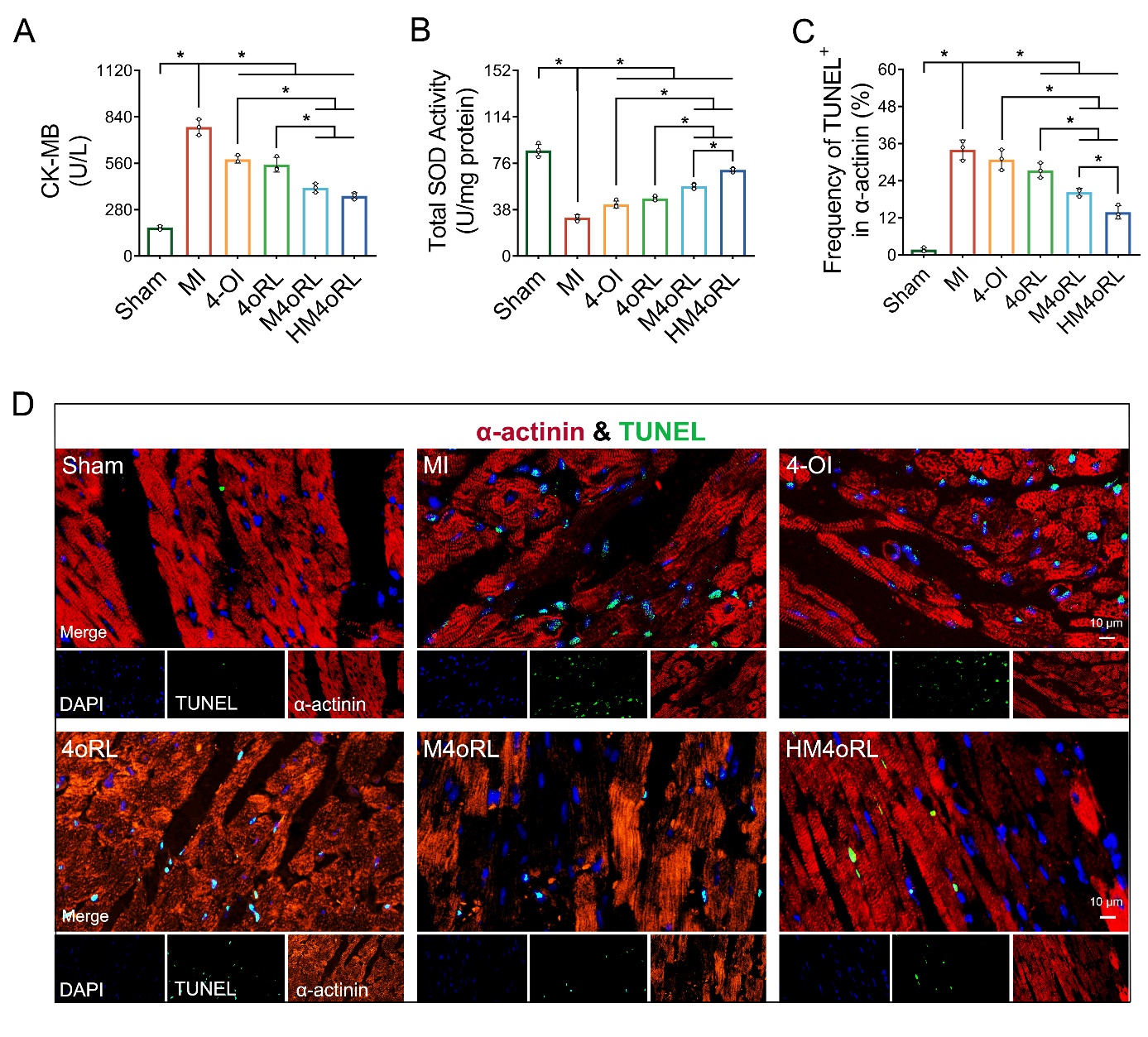


**Figure S13.** (A) Serum myocardial enzyme CK-MB levels after indicated treatments. n=3. (B) Levels of antioxidant enzyme SOD in infarcted myocardial tissue of each group. n=4. (D) Representative immunofluorescent staining images of TUNEL and α-actinin in the heart section, and (C) quantification of TUNEL-positive cells within α-actinin-positive cells in each group. Green, TUNEL; red, α-actinin; blue, DAPI. n=3. Data are presented as mean ± SD. Statistical methods: One-way ANOVA with Tukey’s post-test (A-C). In all panels, * indicates *p* < 0.05.


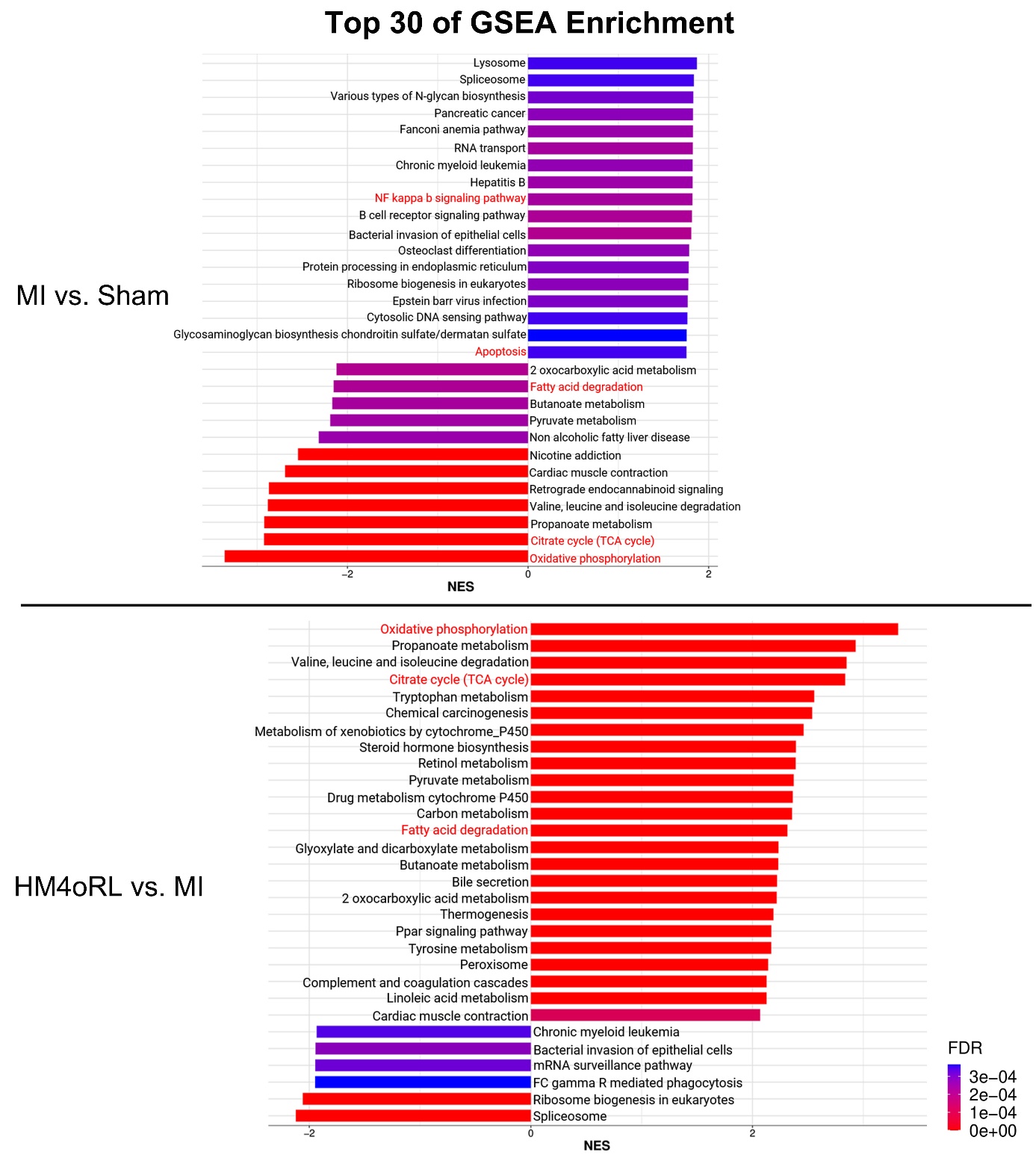
**Figure S14.** Top 30 Gene Set Enrichment Analysis (GSEA) results from transcriptomic data comparisons: (Upper) MI vs. Sham group and (Lower) HM4oRL vs. MI group.


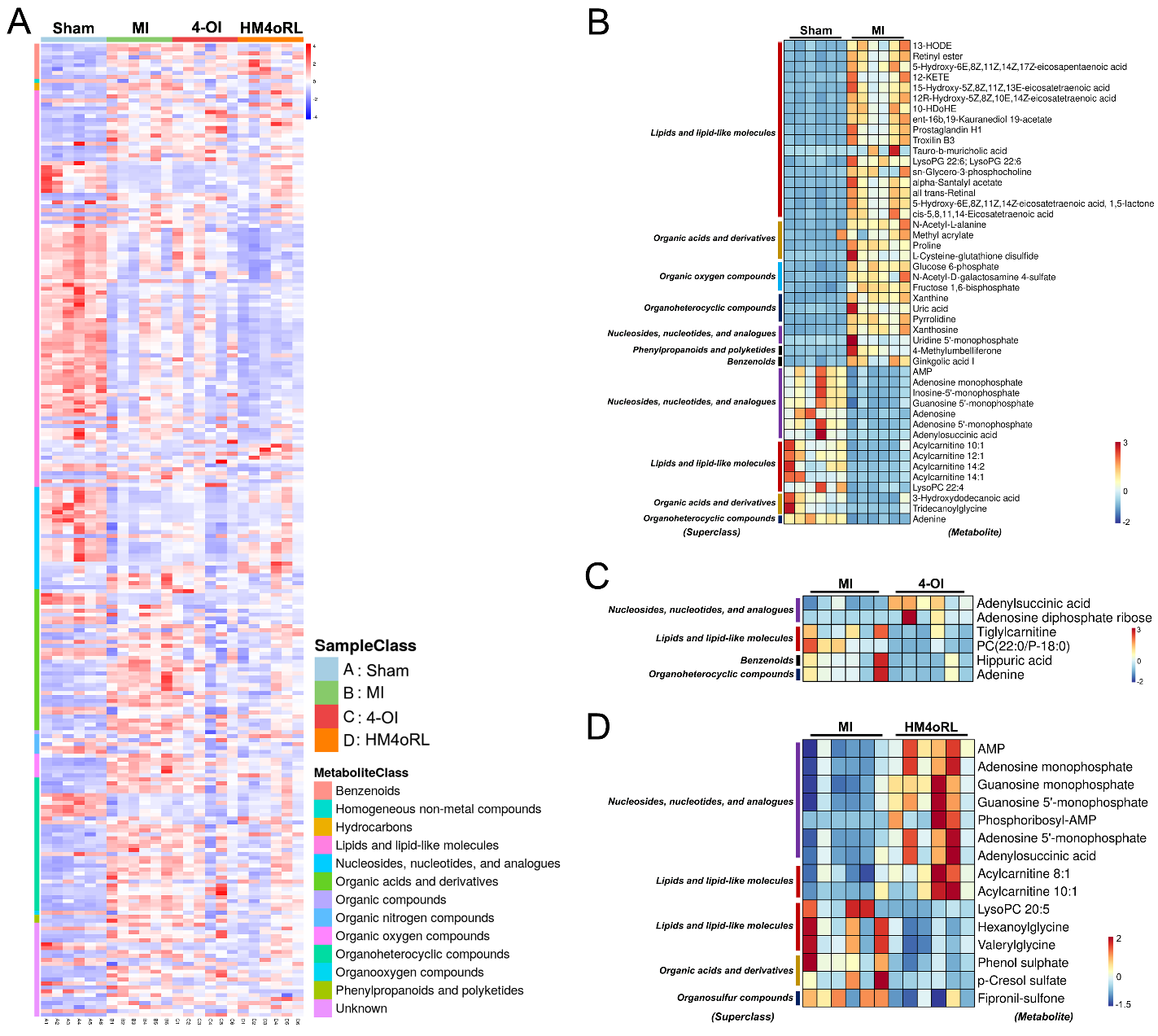


**Figure S15.** (A) Heat map of hierarchical cluster analysis for the Sham, MI, 4-OI, and HM4oRL groups. (B-D) Heatmap showing differentially abundant metabolites derived from metabolomic analysis of the infarcted myocardium in each group. n=6.


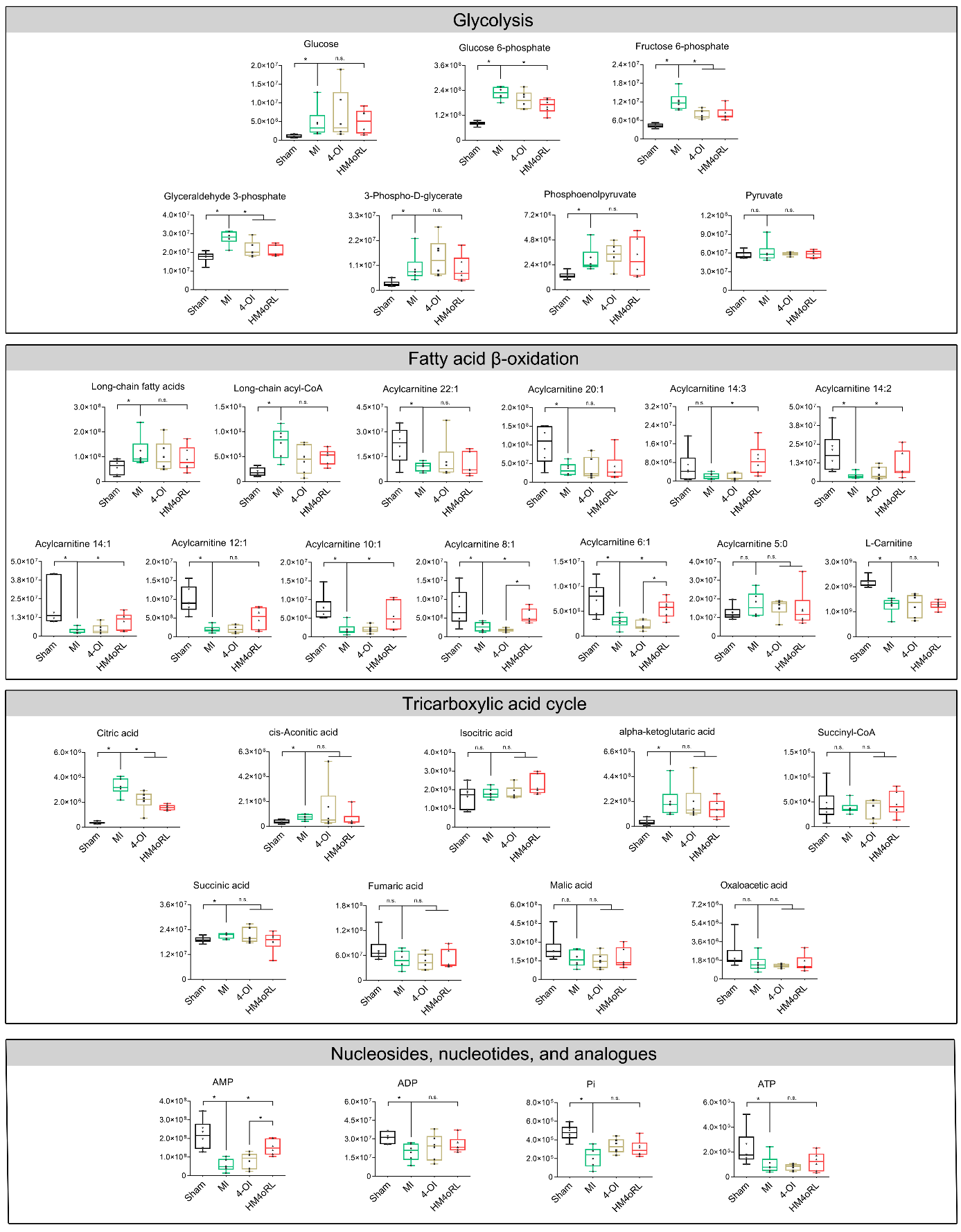


**Figure S16.** Quantitative analysis of metabolites involved in glycolysis, fatty acid β-oxidation, tricarboxylic acid cycle, and nucleosides, nucleotides, and analogues in the 4-OI and HM4oRL groups. n=6. Data are presented as mean ± SD. Statistical methods: One-way ANOVA with Tukey’s post-test, or Tamhane’s post-test. In all panels, * indicates *p* < 0.05, and 'n. s.' indicates no significance.


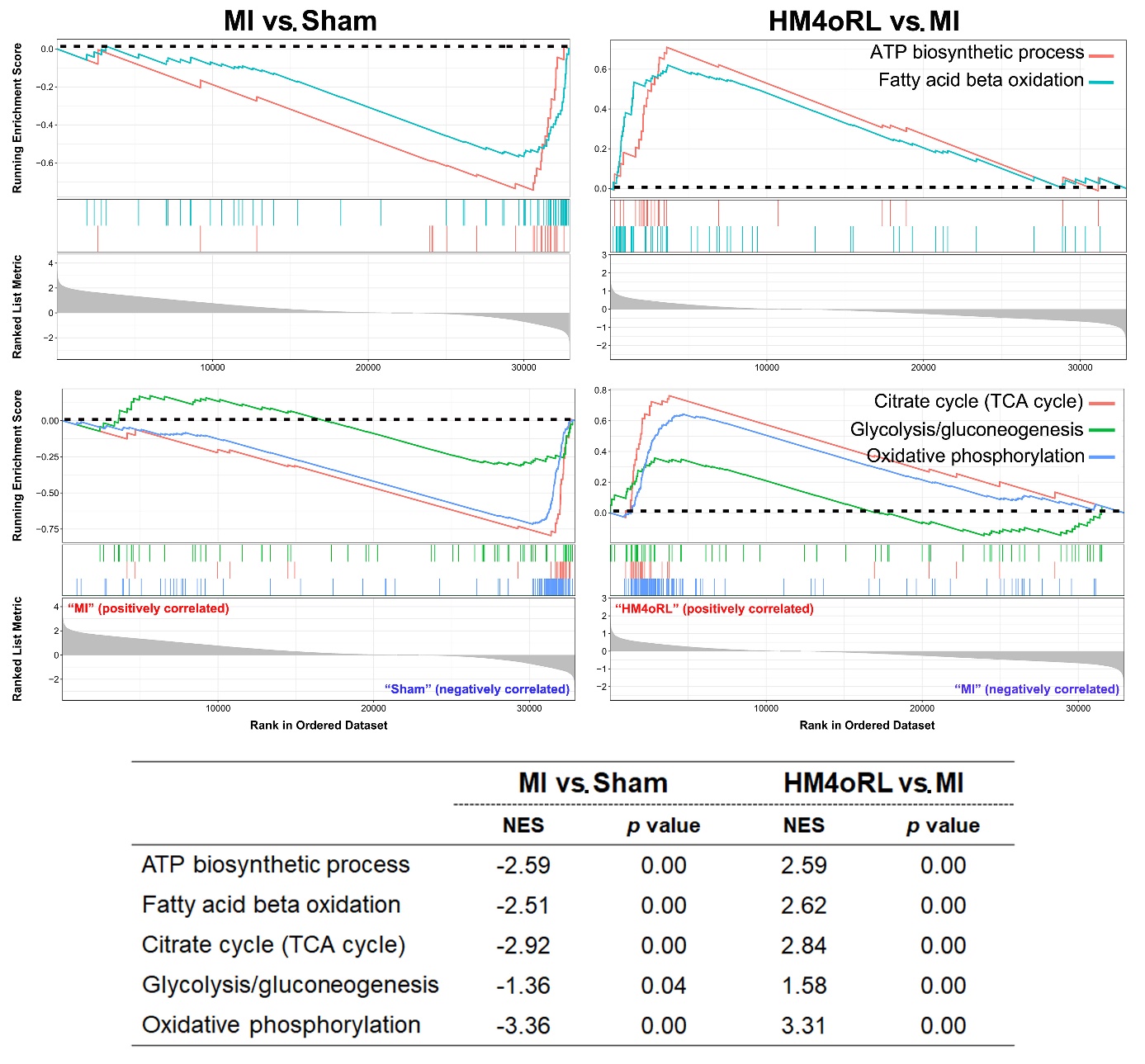
**Figure S17.** Gene Set Enrichment Analysis (GSEA) of the enrichment plots for 'ATP biosynthetic process,' 'fatty acid beta-oxidation,' 'citrate cycle (TCA cycle),' 'glycolysis/gluconeogenesis,' and 'oxidative phosphorylation,' derived from transcriptomic sequencing of the infarcted myocardium in the Sham, MI, and HM4oRL groups. n=4.


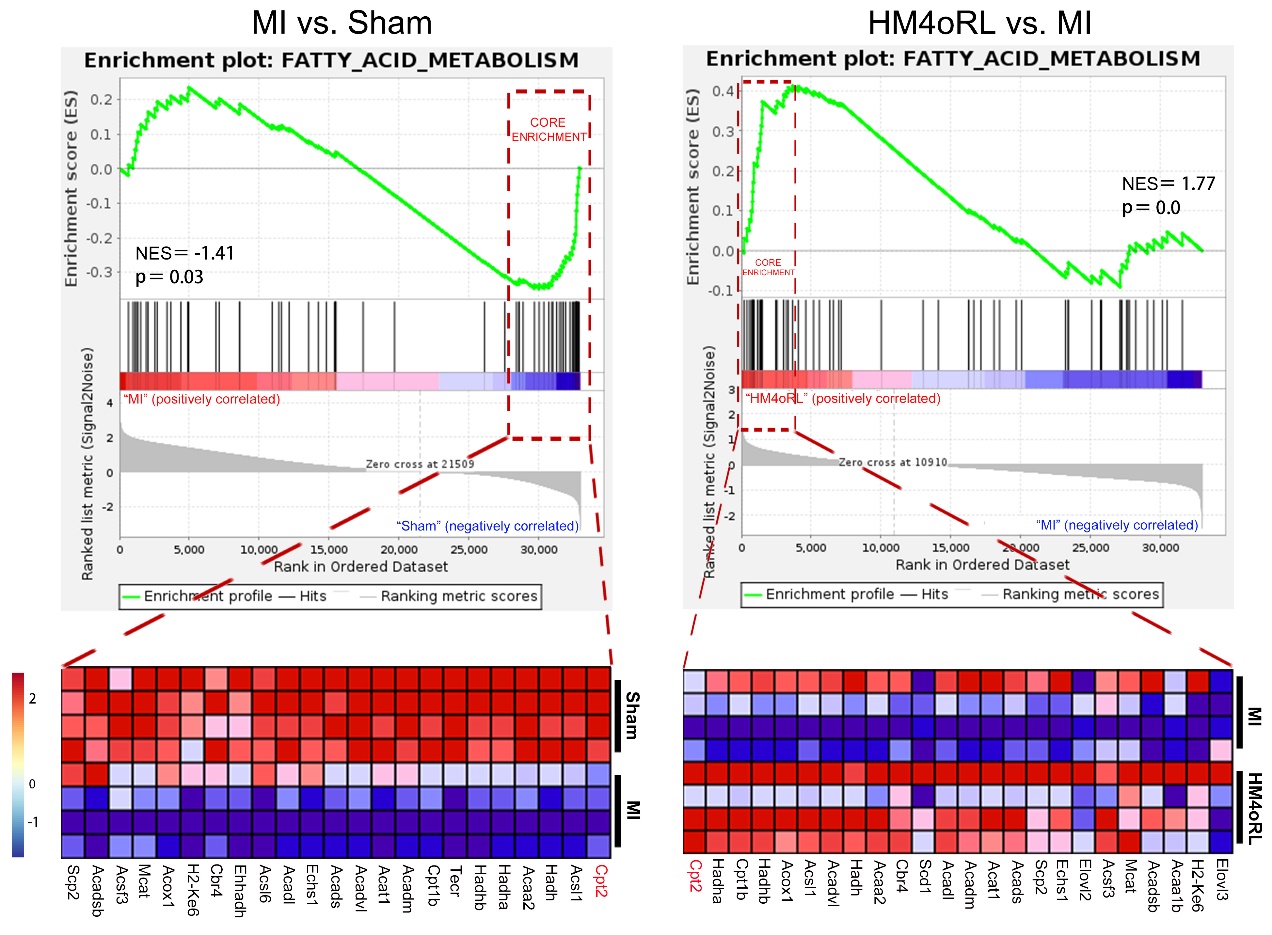


**Figure S18.** Gene Set Enrichment Analysis (GSEA) analysis of enrichment plots related to 'fatty acid metabolism' derived from transcriptome sequencing of the infarcted myocardium in the Sham, MI, and HM4oRL groups, with the corresponding core enrichment genes displayed below. n=4.

**
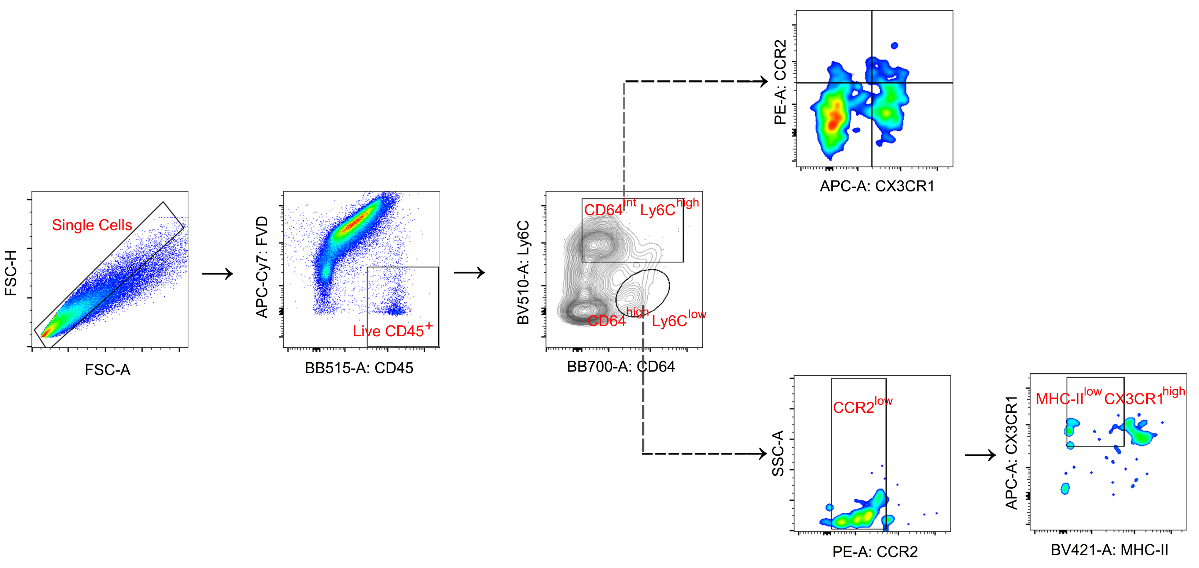
**

**Figure S19.** Gating strategy for identifying live cardiac monocyte (CD45^high^ CD64^int^ Ly6C^high^), monocyte/macrophage (CD45^high^ CD64^high^ Ly6C^low^), and cardiac-resident macrophages (CD45^high^ CD64^high^ Ly6C^low^ CCR2^low^ MHC-II^low^ CX3CR1^high^) in mice hearts 5 days after MI.


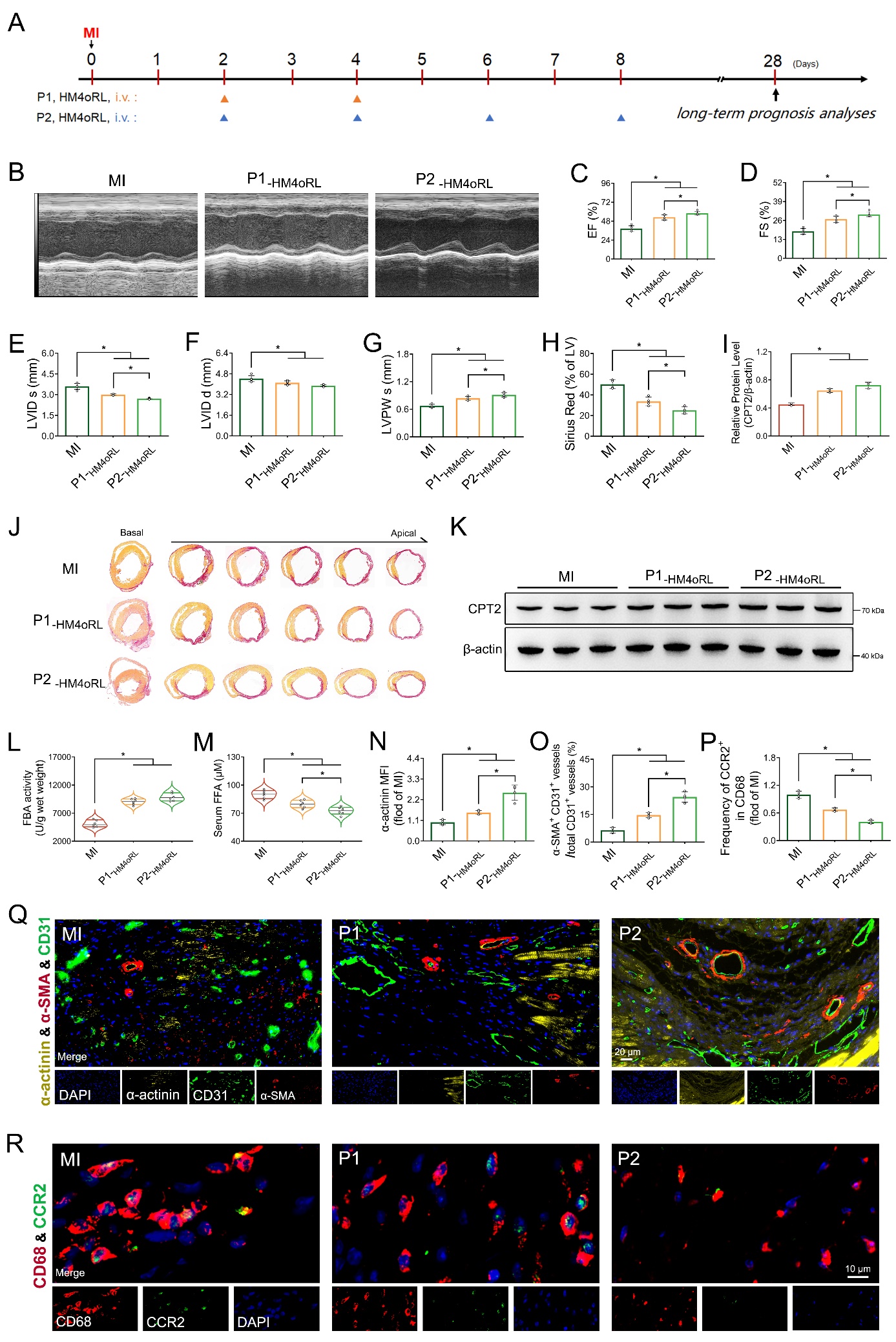


**Figure S20.** (A) A schematic showing the experiment design for delayed injection therapy. (B) Representative M-mode echocardiographic images for each group, captured 4 weeks post-MI. Left ventricular ejection fraction (EF) (C) and fractional shortening (FS) (D) assessed by echocardiography at specified time points post-MI for each group. n=5. Quantification analysis for LVIDs (E), LVIDd (F), and LVPWs (G) based on the M-mode echocardiographic images at 4 weeks post-MI. n=5. Representative Sirius red staining images of myocardial sections (J) and quantitative analysis of myocardial fibrosis size (H) at 4 weeks post-MI. n=4. (I, K) Western blotting analysis of CPT2 protein content alterations in injured myocardium after different treatments. n=3. (L) Levels of FBA activity in injured myocardium after different treatments. n=6. (M) Levels of serum FFA after different treatments. n=6. (N) Representative immunofluorescence images of heart sections triple-stained with fluorescein-labeled α-actinin (yellow), α-SMA (red), CD31(green) and nuclei (blue) at 4 weeks post-MI. Quantification of cardiomyocyte density (α-actinin-positive) (N) and the number of arteries (both α-SMA and CD31 positive) (O) in the infarct zone. (R) Representative double-immunofluorescence staining for CCR2 and CD68 in infarct tissue at 4 weeks post-MI, and (P) quantification of CCR2^+^ macrophages for each group by ImageJ software. Green, CCR2; red, CD68; blue, DAPI. n=4. Data are presented as mean ± SD. Statistical methods: One-way ANOVA with Tukey’s post-test (C-I, L-P). In all panels, * indicates *p* < 0.05.


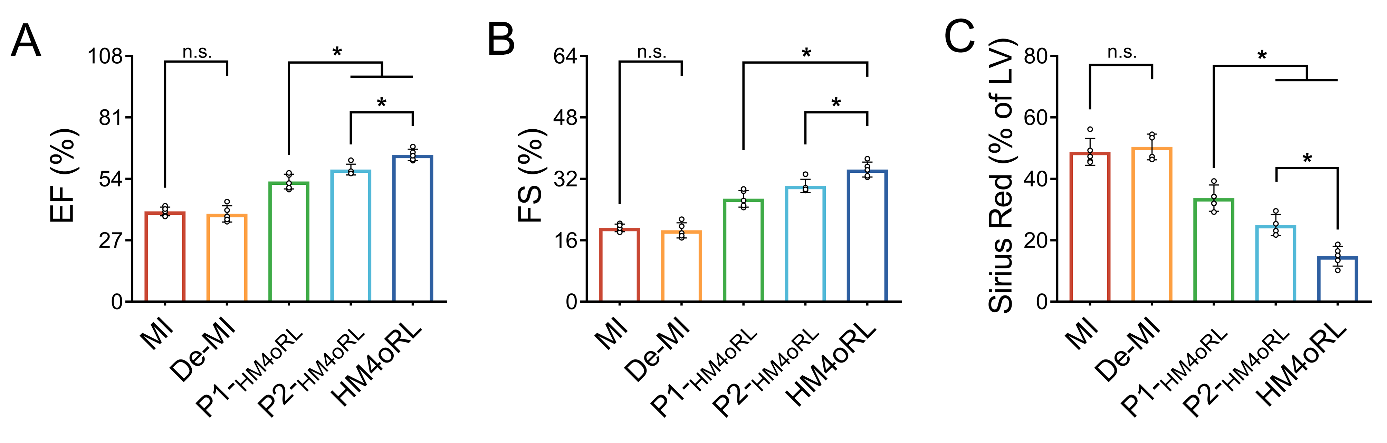
**Figure S21.** Quantitative comparisons of left ventricular ejection fraction (EF) (A), fractional shortening (FS) (B), and Sirius Red staining (C) were performed across various groups. Data for the De-MI, P1-HM4oRL, and P2-HM4oRL groups are derived from panels C, D, and H in Figure S20; data for the MI and HM4oRL groups are obtained from panels C, D, and H in Figure 9. Data are presented as mean ± SD. Statistical methods: One-way ANOVA with Tukey’s post-test (A-C). In all panels, * indicates *p* < 0.05, and 'n. s.' indicates no significance.


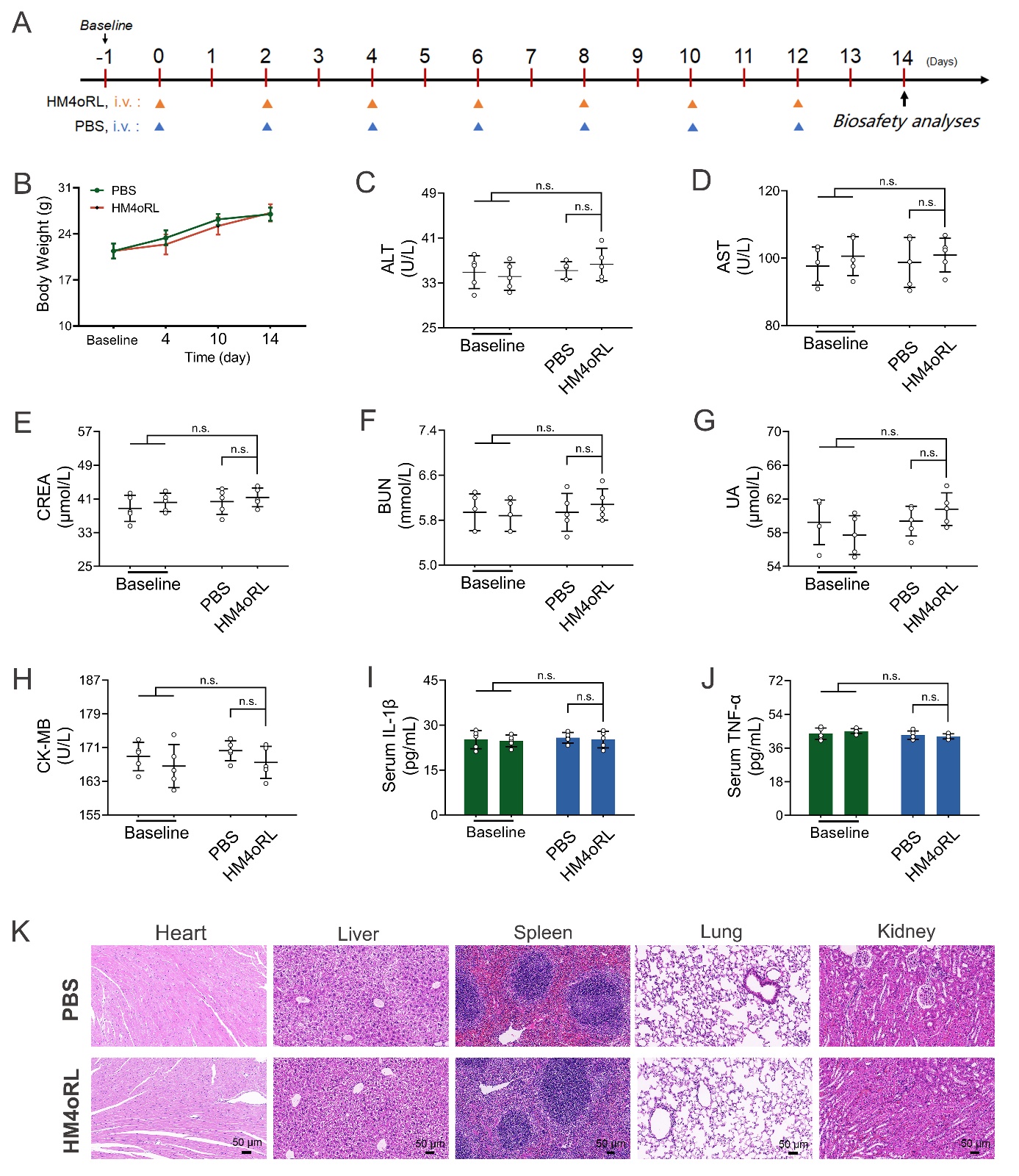
**Figure S22.** (A) Timeline for the animal study. (B) Body weight of mice at indicated time points for each group. n=5. (C-G) Quantitative analysis of serum hematological and biochemical parameters after indicated treatments. n=5. Serum myocardial enzyme CK-MB (H), IL-1β(I), and TNF-α(J) levels after indicated treatments. n=5. (K) H&E staining images of heart, lung, liver, spleen, and kidney collected from mice after indicated treatments. Data are presented as mean ± SD, * indicates *p* < 0.05. Statistical methods: One-way ANOVA with Tukey’s post-test (A), or Tamhane’s post-test (J). In all panels, * indicates *p* < 0.05, and 'n. s.' indicates no significance.

**Supplement- Materials and Methods**

*Materials*: 1,2-Distearoyl-sn-glycero-3-phosphorylcholine (DSPC), cholesterol, 4-Octyl Itaconate (4-OI), 2’,7’-Dichlorodihydrofluorescein diacetate (DCFH-DA) were purchased from MedChemExpress (USA). Tannic acid (TA), MnCl_2_**^.^**4H_2_O, IR780 iodide (IR780) were obtained from Sigma-Aldrich (USA). 1,2-Distearoyl-sn-glycero-3-phosphoethanolamine-thioketal-(polyethylene glycol) (DSPE-TK-PEG, 2000 Da) was customized at Xi’an Ruixi Biotech Co., Ltd (Xian, China). Percoll was purchased from GE Healthcare (USA). Minute™ Plasma Membrane Protein Isolation and Cell Fractionation Kit were obtained from Invent Biotechnologies, Inc. (USA).

*Preparation of Liposomes*: Liposomes were synthesized through the ethanol rapid injection method according to a previously published protocol with slight modifications.^[1]^ Each component-DSPC, cholesterol, DSPE-TK-PEG, 4-OI, and IR780-was dissolved separately in ethanol. DSPC, cholesterol, and DSPE-TK-PEG, in various molar ratios, were then combined with 4-OI or IR780 to a total volume of 0.2 mL, forming the organic phase. This organic phase was rapidly injected into 1.8 mL phosphate-buffered saline (PBS) stirred at 400 rpm with a 200 μL pipette. Immediately after injection, the mixture was subjected to probe ultrasound sonication (JY92-Ⅱ N, Scientz biotechnology, China) under 200 W for a total duration of 5 minutes. Afterwards, the sample solution was stirred in a water bath at 40 ℃. After 4 hours of stirring, the sample was centrifuged at 2000 rpm for 10 minutes, then transferred to a dialysis bag (MWCO 10 KD, Sangon Biotech, China) for 12 hours of dialysis to collect purified 4-OI-loaded liposomes (4-OI@liposomes, 4oRL), IR780-labeled liposomes (IR780@liposomes, IrRL) or unloaded liposomes (unloaded@liposomes, ERL) nanocrystals.

*Synthesis Of MPN-coated Nanoparticles*: Briefly, 2 mL of the above nano solution was first added to 2.38 mL PBS stirred at 700 rpm at room temperature. Next, 0.1 mL of fresh TA solution (10 mg/mL) was added, followed by the addition of 0.5 mL MOPS buffer (0.1 M, pH 8.0) to increase the pH. Immediately afterwards, 0.025 mL of fresh MnCl_2_^.^4H_2_O solution (10 mg/mL) was slowly added to the stirred mixture. After stirring at room temperature for 1 hours, the particles were collected by centrifugation (20 000 rcf, 20 min), washed with PBS three times, and then lyophilized to obtain MPN-coated 4oRL (MPN@4-OI@liposomes, M4oRL), MPN-coated IrRL (MPN@IR780@liposomes, MIrRL) or MPN-coated ERL (MPN@unloaded@liposomes, MRL) nanoparticles.

*Extraction and Activation Protocols of* *Neutrophils from Mouse Bone Marrow*: A density gradient centrifugation method was used to isolate neutrophils from mouse bone marrow.^[2]^ A healthy C57BL/6 mouse, aged 6-8 weeks, was euthanized, and bone marrow cells were harvested from the femurs and tibias. Meanwhile, Percoll separation media were prepared in a sterile 15 mL conical tube with density layers of 52%, 64%, and 72% from top to bottom. Then, 2 mL of the whole bone marrow cell suspension was slowly and gently layered on top of the aforementioned gradient. Horizontal centrifugation was performed at 1545 × g for 30 minutes, yielding a pure, mature neutrophil population retained at the 64%-72% interface. Flow cytometry was used to confirm the purity of the isolated neutrophils. Finally, the collected neutrophils were cultured in 1 × RPMI 1640 medium, which contained 10% FBS and 1% penicillin/streptomycin in a 37℃, 5% CO_2_ incubator.

Given the observation that succinate is the predominant metabolite elevated in the peripheral venous blood of ST-Elevation Myocardial Infarction (STEMI) patients,^[3]^ succinate and lipopolysaccharide (LPS) were utilized to replicate the activation stimulus of neutrophils. Briefly, the collected neutrophils were then stimulated with succinate (1 mM) and LPS (100 ng/mL) for 12 hours to induce activation.

*Extraction of the cell* *plasma membrane*: As described in a previous publication,^[4]^ the plasma membranes of activated neutrophils and untreated RAW 264.7 cells were extracted using the Minute plasma membrane protein isolation and cell fractionation kit, following the manufacturer’s protocol respectively. The extracted plasma membrane proteins were quantified by a BCA protein assay kit (Beyotime, P0010), then stored at -80°C for subsequent studies.

*Preparation of Hybrid Membrane-Coated* *Nanoparticles*: Activated neutrophil plasma membrane (ANM), macrophage plasma membrane (MM), M4oRL or MIrRL were each dissolved in 4°C PBS, respectively. Based on a mass ratio of 300 μg: 300 μg: 600 μg,^[5]^ ANM, NM and nanoparticles were uniformly mixed in 1 mL PBS. The resulting mixture was then sequentially extruded through polycarbonate membranes with pore sizes of 400 nm and 200 nm for 10 passes using a filter (Avanti Polar Lipids, Inc). Excess free ANM, NM were removed by centrifugation (20 000 rcf, 20 min), and the precipitates were washed with 4°C PBS to obtain hybrid membrane-coated M4oRL (HM@MPN@4-OI@liposomes, HM4oRL) or hybrid membrane-coated MIrRL (HM@MPN@IR780@liposomes, HMIrRL).

*Characterization of Nanoparticles*: Nanoparticle hydrodynamic size, size distribution and surface zeta potential were measured by a dynamic light scattering (DLS) using a Nanobrook Zeta Plus analyzer (Brookhaven Instruments). Transmission electron microscope (TEM) (JEOL JEM-2100F) was employed to visualize the morphology of nanoparticles. Fourier transform infrared spectroscopy (FT-IR) spectra were recorded on the Thermo Nicolet iS5 spectrometer in the 4000-400 cm^-1^ range. X-ray photoelectron spectroscopy (XPS) spectra was determined on a Thermo Scientific Nexsa surface analysis system. ICP-MS was performed on Agilent 7800 ICP-MS. T1 relaxation time was obtained from a 9.4 T Micro MR scanner (BioSpec 94/30 USR, Bruker, Germany).

The powder yield of the nanoparticles was calculated by comparing the total weight of the collected dry nanoparticle powder to the total weight of raw materials added to the liquid reaction system.^[6]^

Total weight of the collected dry nanoparticle powder

Total weight of added raw materials

×100

Powder yield (%)＝

*Co-Localization of Hybrid Membrane with Nanoparticles*: After extracting the cell plasma membrane, the ANM was labeled with DiO, and the MM was labeled with DiI. Subsequently, the ANM and MM were co-extruded with MIrRL according to the aforementioned process. After centrifugation to remove the excess membrane, HMIrRL was visualized by confocal laser scanning microscopy (Leica, Germany).

*Antioxidant Activity Evaluation of* *Nanoparticles*: According to a rapid 2, 2′-azinobis-3-ethylbenzthiazoline-6-sulfonic acid (ABTS) method reported in a previous study,^[7]^ the antioxidant capacity of nanoparticles was measured via an antioxidant capacity assay kit (Beyotime Technology Inc., China). Briefly, ABTS**^·^**^+^ radical solution was mixed separately with 4-OI, 4oRL, M4oRL, MRL, or HM4oRL. After 20-minute reaction at room temperature, the absorbance at 414 nm was measured via a cell imaging multi-mode reader.

Electron paramagnetic resonance (EPR) analysis was used to measure the **^·^**OH radical generated by the irradiation of H_2_O_2_ (25 mM, 50 μL) with a Xenon lamp, or the **^·^**O_2_¯ radical generated from the production of xanthine oxidase (0.4 U/mL, 50 μL) and xanthine (2 mM, 50 μL) based on the previously described method.^[7]^ The generated **^·^**OH radical was immediately mixed with a selected radical scavenger (100 μL, 100 μg/mL) and DMPO (25 mM, 50 μL). The generated **^·^**O_2_¯ radical was immediately mixed with the aforementioned tested radical scavenger and BMPO (50 mg/mL, 10 μL). Finally, the EPR data were recorded on a Bruker A300 EPR spectrometer.

*MR Relaxivity Measurements*: The concentration of Mn ions in HM4oRL NPs was measured by ICP-MS. Then, HM4oRL NPs solutions (pH 7.4 and pH 6.4) with various concentrations of Mn ions (0-0.4 mM), were prepared and placed at room temperature for 4 hours before being imaged with a T1 map sequence by a 9.4 T Micro MR scanner. The longitudinal relaxivity (r1) was obtained from the slopes of 1/T1 (s^-1^) to the Mn ions concentrations.

*Drug Loading and Release Assays*: The standard curve for 4-OI was established by high-performance liquid chromatography (HPLC) (Agilent 1260, USA). The chromatographic conditions were as follows: Ultimate XB-C18 (150×4.6mm, 5um), column temperature at 35℃, mobile phase consisting of methanol and HClO_4_ (75 : 25, v/v), an injection volume of 20 µL, a flow rate of 1.0 mL/min, and a detection wavelength of 210 nm. The NPs were lysed with 4% TritonX-100, and the supernatant was taken for HPLC after centrifugation. The drug loading efficiency was calculated as the following formula:

Drug loading efficiency (%)＝Weight of loaded drug / Weight of NPs × 100%

*In vitro* release experiments were conducted to assess the drug release property of M4oRL and HM4oR using the dialysis method. M4oRL and HM4oR NPs were dispersed in 3 mL of PBS (pH = 7.4), then placed into a dialysis bag (MWCO = 10 KD). The basic release medium was PBS containing 0.5% Tween-80 at pH = 7.4. Depending on the experimental purpose, hydrochloric acid was added to lower the pH of the solution, or H_2_O_2_ was added to simulate the ROS environment. The dialysis bag was completely submerged in the adjusted release medium and shaken at 100 rpm at 37°C. At different time intervals, 0.5 mL of dialysate was extracted to detect the amount of released 4-OI by HPLC, and an equal volume was replenished to maintain a constant volume.

*Cell Culture*: Murine RAW 264.7 (RAW) macrophage cell line and H9c2 cardiomyocyte cell line were obtained from the Cell Bank of the Chinese Academy of Sciences (Shanghai, China), while human umbilical vein endothelial cells (HUVECs) were from Procell (Wuhan, China). RAW 264.7 and H9c2 were cultured in Dulbecco’s modified Eagle’s medium (DMEM, Gibco, USA) containing 4.5 g/L D-glucose supplemented with 10% foetal bovine serum (FBS, Gibco, USA), 1% penicillin/streptomycin. HUVECs were cultured in HUVEC cell-specific medium purchased from Procell (CM-0122, Wuhan, China). All cells were maintained at 37 °C in a humidified 5% CO_2_ atmosphere.

*Cell treatment*: The hypoxia-induced cardiomyocyte injury model, created by Anaero Pack method,^[8]^ was used to mimic the MI model *in vitro*. Briefly, when H9c2 cells seeded in a 6-well plate reached a confluency of more than 80%, the culture medium was changed to serum-free, low-glucose DMEM to serve as the anoxic solution. The cells were then placed in a sealed container with an Anaero Pack (Mitsubishi Gas Company, Tokyo, Japan) at 37°C for 12 h. The treatment measures for each group were as follows: (1) Control group (Control): normal air with conventional culture medium; (2) hypoxia group (Hyp): hypoxia with anoxic solution and an equivalent volume of PBS; (3) 4-OI group (4-OI): hypoxia with anoxic solution containing 75 μM 4-OI; (4) 4oRL group (4oRL): hypoxia with anoxic solution containing 4oRL NPs (75 μM 4-OI); (5) M4oRL group (M4oRL): hypoxia with anoxic solution containing M4oRL NPs (at a concentration equivalent to 4oRL); (6) HM4oRL group (HM4oRL): hypoxia with anoxic solution containing HM4oRL NPs (at a concentration equivalent to 4oRL).

For RAW 264.7 cells, a stimulation model was established by treating the cells with 200 ng/mL LPS for 24 hours, maintained at 37 °C in 5% CO_2_ atmosphere. The treatment protocols for each group were as follows: (1) Control group (Control): fresh conventional culture medium; (2) LPS group (LPS): co-treated with LPS and an equivalent volume of PBS; (3) 4-OI group (4-OI): co-treated with LPS and 75 μM 4-OI; (4) 4oRL group (4oRL): co-treated with LPS and 4oRL NPs (75 μM 4-OI); (5) M4oRL group (M4oRL): co-treated with LPS and M4oRL NPs (at a concentration equivalent to 4oRL); (6) HM4oRL group (HM4oRL): co-treated with LPS and HM4oRL NPs (at a concentration equivalent to 4oRL).

*Cell Viability Measurement*: Cell viability assessment, following cytotoxicity and cell injury tests, was performed using the Cell Counting Kit-8 (CCK-8) assay. The cytotoxicity of 4oRL, M4oRL, and HM4oRL NPs was tested on the H9c2 cardiomyocyte cell line, the HUVECs cell line, and RAW 264.7 cell, respectively. Cells were seeded in 96-well plates and, once they reached a confluency of more than 80%, the culture medium was replaced with different concentrations of the 4oRL, M4oRL, and HM4oRL NPs in fresh culture medium. The cells were then incubated for 24 hours at 37 °C in a humidified 5% CO_2_ atmosphere. This was followed by the CCK-8 test based on protocols. For cells after stimulation, the CCK-8 test was performed immediately after the end of treatment.

*Cellular Uptake*: IR780-labeled MIrRL (MPN@IR780@liposomes, MIrRL) and HMIrRL (HM@MPN@IR780@liposomes, HMIrRL) NPs were used to evaluate the uptake of these NPs by impaired cardiomyocytes (hypoxia-exposed H9c2 cells) and inflammatory macrophages (LPS-treated RAW 264.7 cells). Flow cytometry (Beckman Coulter, USA) was used for quantification, while laser confocal microscopy was employed for qualification.

Cells were seeded in 6-well plates or confocal dishes. Once established, the media was replaced with fresh media containing the desired concentrations of MIrRL or HMIrRL NPs. The cells then underwent the stimulation model for either 4 or 12 hours, depending on the specific experimental requirements.

*Lacate Dehydrogenase (LDH) Activity Assay*: LDH activity was measured as an indicator of cardiomyocyte damage post-hypoxia using an LDH activity assay kit (Solarbio, China). After hypoxia, the cell supernatant from each group was separately centrifuged and then tested using the kit in accordance with the provided instructions.

*Superoxide Dismutase (SOD) Activity Assay*: The antioxidant capacity of H9c2 cells and infarcted myocardial tissue was evaluated by antioxidant enzyme SOD via a SOD assay kit (Beyotime, China). In accordance with the instructions, the SOD activity of treated H9c2 cells or myocardial tissue samples was quantified in each group.

*ROS Detection*: *In vitro*, intracellular ROS levels were assessed using DCFH-DA, while mitochondrial superoxide production was analyzed with the MitoSOX fluorescent probe (Yeasen Biotechnology, China). For flow cytometry, following the treatment, the cells were trypsinized, washed and centrifuged, resuspended in 0.2 mL PBS and stained with 5μM DCFH-DA for 30 min at 37℃. After washing the cultured cells three times with PBS, intracellular ROS levels were quantified using a flow cytometer. For laser confocal imaging, the tested cells were incubated with 5μM MitoSOX Red reagent for 10 min at 37℃ in absence of light, followed by gentle washing three times. Images were observed using a fluorescence microscope and analyzed using ImageJ software.

*In situ* ROS production in cardiac tissue was evaluated using dihydroethidium (DHE). Briefly, flash-frozen cardiac tissue sections were incubated with DHE, and images were captured using a confocal microscope.

*Cell Apoptosis Assay*: The apoptosis of H9c2 cells induced by hypoxia was detected using an Annexin V/propidium iodide (PI) Apoptosis Detection Kit (KeyGEN, China). The H9c2 cells were digested with EDTA-free trypsin, washed, and then stained with Annexin V and PI reagent working solution following the provided instructions. Finally, approximately 10,000 cells were collected for each sample and analyzed by flow cytometer.

*Cellular ATP Assay*: After exposure to hypoxia, the intracellular ATP level of H9c2 cells were determined by an ATP Assay Kit (S0026, Beyotime, China). The tested H9c2 cells were thoroughly lysed on ice. The supernatant was collected after centrifugation, then mixed with the ATP detection working solution in a black 96-well plate (3603, Corning, USA) according to the manufacturer’s protocol. The relative luminescence units (RLU) were measured using a microplate reader with a luminometer function (Tecan Infinite® 200 PRO, Switzerland). Finally, the ATP concentration of each sample was calculated based on the calibration curve.

*Enzyme‑Linked Immunosorbent Assay (ELISA)*: The supernatants from the treated cell culture or the serum from mice were collected for the detection of IL-1β (VAL601 and RLB00, Novus Biologicals, USA), TNF-α (abs520010, Absin, China), and IL-10 (VAL605, Novus Biologicals, USA) according to their respective protocols.

*MI Surgery and Treatment*: The mouse MI model was established by permanent ligation of the left anterior descending (LAD) coronary artery through open thoracic surgery. Anesthesia was maintained with 0.8-1% isoflurane inhalation using a ventilator (SAR-1000, YUYAN INSTRUMENTS, Shanghai, China) throughout the thoracotomy. The needle was inserted into the left ventricular myocardium, on the left side of the anterior interventricular groove-shaped area of the heart surface, approximately 1 mm below the left atrial appendage. A 6-0 silk suture was then passed under the interventricular groove, and emerged on the right side, encircling the path of the coronary artery, and a knot was tied to occlude the LAD. Air was expelled from the chest prior to closure. Successful ligation of LAD was confirmed by an animal electrocardiogram machine (HB-A3, Guangdong, China). Half an hour after the chest was closed, the ventilator was removed. Subsequently, the mice were randomly allocated to different groups, and treatments were administered in accordance with the established protocol.

When determining the dosage of 4-OI, we referred to the latest review and studies related to inflammation and ischemic injury, which provided a basis for selecting doses of 25, 50, and 200 mg/kg.^[9]^ The primary objective of this study is to effectively regulate the acute immune-inflammatory response, which predominantly occurs within the first 3-7 days following an acute MI. Therefore, we administered 50 mg/kg of 4-OI daily for the first 7 days post-MI using intraperitoneal injection. The intervention groups were organized as follows: (1) Sham group (Sham): a suture was passed under the LAD without occlusion; (2) MI group (MI): LAD was ligated, and 200 μL PBS was administered intravenously every other day; (3) 4-OI group (4-OI): LAD was ligated, and 50 mg/kg of 4-OI was administered via intraperitoneal injection daily; (4) 4oRL group (4oRL): LAD was ligated, and 4oRL NPs was administered intravenously (at a concentration equivalent to HM4oRL) every other day; (5) M4oRL group (M4oRL): LAD was ligated, and M4oRL NPs was administered intravenously (at a concentration equivalent to HM4oRL) every other day; (6) HM4oRL group (HM4oRL): LAD was ligated, and HM4oRL NPs was administered intravenously every other day.

*Assessment of Myocardial Targeting Ability and Distribution of Nanoparticles*: To assess the myocardial targeting ability and distribution of nanoparticles, MIrRL or HMIrRL NPs were injected via the tail vein. At different time points, the treated mice were anesthetized, and fluorescence images of both the precardiac region *in vivo* and the ex vivo heart were captured using an IVIS imaging system (In-Vivo Master, China). At the 24-hour and 48-hour time points post-injection, the mice were euthanized, and the kidneys, lungs, spleen, and liver were collected from each animal to observe the fluorescence distribution via the IVIS imaging system. The analysis and quantification of the obtained fluorescence images were performed by ImageJ.

For immunohistochemical staining to assess nanoparticle co-localization, hearts from treated mice were harvested 12 hours post-MI and prepared as frozen sections. These sections were stained for markers including α-actinin (cardiomyocytes), CD68, and F4/80 (monocyte/macrophage markers). Co-localization of HMIrRL with these markers was then examined using fluorescence microscopy.

*Triphenyltetrazolium Chloride Staining*: After freezing the harvested heart at -80°C for 30 minutes, it was sectioned into approximately 1-2 mm slices, perpendicular to the long axis of the heart. The slices were then subjected to fluorescence imaging, followed by incubation in a 1.5% triphenyltetrazolium chloride (TTC) solution at 37°C for 10 minutes, and then fixed in 4% (*w/v*) paraformaldehyde.

*In Vivo Cardiac MR Imaging and Data Analysis*: *In vivo* mouse cardiac MRI scans were performed 3 days after the induction of MI with a 9.4 T Micro MR scanner (BioSpec 94/30 USR, Bruker, Germany) by intravenous injection of HM4oRL nanoparticles or MnCl_2_ ([Mn] = 10 mM/Kg, 150 μL). Short-axis T1-weighted images were acquired at designed time points with ECG-triggering and respiratory gating. The detailed parameters for T1-weighted FLASH sequence were set as follows: TE = 1.6 ms; TR = 45 ms; slice thickness = 1 mm; FOV = 2.5 cm × 2.5 cm; FA = 40°; matrix = 192 × 192. The contrast to noise ratios (CNR) were calculated as μ / σ noise (μ indicated the mean signal intensity of the infarcted or remote area; σ noise indicated the standard deviation of the noise).^[10]^

*Cardiac Function Assessment*: The cardiac function of mice was measured using a Vevo 2100 imaging system (VisualSonics, Toronto, Canada), with anesthesia maintained through an inhalation mixture of isoflurane and oxygen. The left ventricle ejection fraction (EF) and fractional shortening (FS) were measured by a two-dimensional M-mode and high-frequency transducer in either the long axis or short axis view of the left ventricle in mice.

*Fructose-Bisphosphate Aldolase Activity*: The activity of fructose-bisphosphate aldolase (FBA) in myocardial tissue was quantified using a fructose-1,6-bisphosphate aldolase (EC4.1.2.13) assay kit (BC2275, Solarbio, China). After removing the blood and clots from the harvested myocardial tissue, the tissue was weighed. The myocardial tissue was then lysed according to the reagent kit instructions, and the total FBA enzyme activity was measured in the resulting lysate. Finally, the enzyme activity was calculated relative to the sample mass.

*Serum Free Fatty Acid Assay*: The concentration of free fatty acids in mouse serum was measured using the Amplex Red Free Fatty Acid Assay Kit, following the manufacturer’s protocol (S0215, Beyotime, China).

*Histological Staining*: The tested hearts fixed with 4% polyformaldehyde, sectioned approximately 5 μm thick, then deparaffinized and stained with Sirius red or Masson’s trichrome. The scar area in each sample was measured by the ImageJ according to the method reported in previous study.^[11]^

*Immunofluorescence Staining*: Sections made from polyformaldehyde-fixed hearts were blocked with 5% bovine serum albumin (BSA) for half an hour, washed, and then incubated with the primary antibody: NLRP3, α-actinin, F4/80, CD206, Collagen I, Collagen III, CD31, VEGF, CD68, GSDMD, CD4, α-SMA, and FoxP3 overnight at 4℃. For histochemistry, the corresponding sections were incubated with HRP-conjugated antibodies. For immunofluorescence, the sections mentioned above were washed with PBS and subsequently incubated with the corresponding fluorescent secondary antibodies, followed by nuclear staining using DAPI. An *in situ* apoptosis detection kit (G1501, Servicebio Technology Co., Ltd. Wuhan, China) was performed for terminal deoxynucleotidyl transferase dUTP nick end labeling (TUNEL) staining. The myocardial infarction area was imaged using a fluorescence microscopy (Nikon, Tokyo, Japan), and subsequent data quantification was performed through ImageJ.

*Biochemical Parameter and Histomorphology Analyses*: After the entire course of treatment, the mice were euthanized, and blood serum as well as major organs were collected. The serum alanine aminotransferase (ALT), aspartate aminotransferase (AST), creatinine (CREA), uric acid (UA), blood urea nitrogen (BUN) levels were measured using an automatic biochemical analysis (Rayto Life and Analytical Sciences, China). Meanwhile, the myocardial enzyme CK-MB in mouse serum was also measured by this automatic biochemical analysis. The harvested organs (heart, liver, spleen, lung, kidney) were fixed with 4% polyformaldehyde and then stained with H&E.

*Untargeted Metabolomics*: The metabolites of the infarcted myocardial tissue were extracted with 50% methanol buffer. Pooled quality control (QC) samples were also prepared by respectively mixing 10 μL of supernatants from the aforementioned extraction mixture. An ACQUITY UPLC T3 column (100mm*2.1mm, 1.8µm) (Waters, Milford, USA) equipped with a Vanquish Flex UHPLC system (Thermo Fisher Scientific, Bremen, Germany) was used for all chromatographic separations of all the samples. A high-resolution tandem mass spectrometer Q-Exactive (Thermo Scientific, Germany), operated in both positive and negative ion modes, was employed for the detection of eluted metabolites from the column. The peak grouping, peak picking, retention time correction, second peak grouping, annotation of isotopes and adducts were performed using XCMS software. The metabolomic analyses were performed by LC-Bio (Hangzhou, China), while the metabolites with variable importance in projection (VIP) values ≥ 1 and p value < 0.05 were used as criteria to identify differentially expressed metabolites.

*Transcriptome Sequencing*: RNA sequencing was conducted on the illumina Novaseq^TM^ 6000 platform by LC Bio Technology CO.,Ltd. Total RNA was separately extracted from the treated neutrophils or infarcted myocardial tissue using Trizol reagent (15596018, Thermo Fisher Scientific). The quantity and purity of total extracted RNA were assessed using NanoDrop ND-1000 (NanoDrop, Wilmington, USA), and the integrity of RNA was evaluated by Bioanalyzer 2100 (Agilent, USA). Subsequently, the 2×150bp paired-end sequencing (PE150) was performed on an Illumina Novaseq™ 6000 according to the manufacturer’s instructions. The initial RNA sequencing data were filtered to obtain high-quality sequencing data (Clean Data). The Clean Data were aligned with the mouse reference genome data, followed by bioinformatics analysis, which included the quantification of gene abundance, differentially expressed genes (DEGs) analysis, relationship analysis of samples, GO enrichment analysis, pathway enrichment analysis (KEGG), gene set enrichment analysis (GSEA), alternative splicing analysis and single-nucleotide polymorphism (SNP) analysis.

*Integrative analysis of metabolomics and transcriptomics*: Based on the original data from the Sham group, MI group and HM4oRL group, integrated analyses of metabolomics (n = 6 per group) and transcriptomics (n = 4 per group) was performed by LC Bio Technology CO.,Ltd. Common pathway information was obtained from the KEGG database, where all DEGs and differentially abundant metabolites (DAMs) were simultaneously mapped through the Venn diagram.

*Western Blot*: Proteins were extracted from infarcted cardiac tissue, H9c2 cells, activated neutrophil plasma membrane, and macrophage plasma membranes. The concentrations of the extracted proteins were then determined. Then, the denatured proteins (30 μg protein/lane) were run onto 10% SDS-PAGE and the separated proteins were transferred to PVDF membranes using a Mini-PROTEAN Tetra System. After blocking with Buffer (P0023B, Beyotime Technology Inc., China), the primary antibodies were diluted with the SignalUp™ Primary Antibody Dilution Buffer (P0273, Beyotime Technology Inc., China) and incubated overnight. These primary antibodies included NLRP3 (1:1000, ab263899, Abcam), phospho AMPK alpha (1:2000, ab133448, Abcam), IL-1 beta (1:1000, ab234437/ab254360, Abcam), GSDMD (1:1000, ab209845/ab219800, Abcam), AMPK alpha (1:2000, 10929-2-AP, Proteintech), Caspase 1/p20/p10 (1:3000, 22915-1-AP, Proteintech), CPT2 (1:3000, 226555-1-AP, Proteintech), Integrin β-1 (1:10000, 12594-1-AP, Proteintech), Integrin α 5 (1:2000, 10569-1-AP, Proteintech), Integrin α X (1:500, PAB39261, Bioswamp), and Beta Actin (1:20000, 81115-1-RR, Proteintech). The obtained membranes were washed and incubated with horseradish peroxidase-conjugated secondary antibody, then visualized using a Tanon 5200 Multi imaging system (Tanon, Shanghai, China). Finally, the intensity of the bands was analyzed via ImageJ.

*Flow Cytometry*: Single cell suspensions were generated from the ventricular myocardium at the level of the ligation line and the subsequent ventricular tissue, using a Heart-specific Tissue Dissociation Kit (Bio-Leader Incorporation, Jiangxi, China). 0.2 mL of FACS buffer containing 1×10^6^ cells was loaded into seven EP tubes, respectively, each blocked with anti-CD16/32 antibody (101320, BioLegend). The cells were incubated with the following reagents separately: Fixable Viability Dye (423106, BioLegend), FITC anti-mouse CD45 (103108, BioLegend), PerCP/Cyanine5.5 anti-mouse CD64 (139308, BioLegend), Brilliant Violet 510™ anti-mouse Ly-6C (128033, BioLegend), Alexa Fluor® 647 anti-mouse CX3CR1 (149004, BioLegend), PE anti-mouse CD192 (CCR2) (150610, BioLegend) and Brilliant Violet 421™ anti-mouse I-A/I-E (107631, BioLegend), then ran on the BD FACSymphony^TM^ A5 platform to adjust the flow cytometry voltage compensation for the following samples. For sample, after being blocked, the above seven reagents were added, then analysed by FACSymphony^TM^ A5 platform. All acquired data were analysed using FlowJo v.10 (Tree Star.).

**References**

[1] E. Pipó-Ollé, P. Walke, M. K. Notabi, R. B. El-Houri, M. Østergaard Andersen, D. Needham, E. C. Arnspang, *J Vis Exp* **2019**, (144).

[2] N. D. J. Ubags, B. T. Suratt, *Methods Mol Biol* **2018**, *1809*, 45.

[3] M. Kohlhauer, S. Dawkins, A. S. H. Costa, R. Lee, T. Young, V. R. Pell, R. P. Choudhury, A. P. Banning, R. K. Kharbanda, K. Saeb-Parsy, M. P. Murphy, C. Frezza, T. Krieg, K. M. Channon, *J Am Heart Assoc* **2018**, *7* (8).

[4] a) F. Li, T. Chen, F. Wang, J. Chen, Y. Zhang, D. Song, N. Li, X. H. Lin, L. Lin, J. Zhuang, *ACS Appl Mater Interfaces* **2022**, *14* (19), 21860; b) X. Xu, R. Zhang, X. Yang, Y. Lu, Z. Yang, M. Peng, Z. Ma, J. Jiao, L. Li, *Adv Healthc Mater* **2021**, *10* (18), e2100518.

[5] Y. Yin, W. Tang, X. Ma, L. Tang, Y. Zhang, M. Yang, F. Hu, G. Li, Y. Wang, *Chemical Engineering Journal* **2022**, *433*.

[6] S. S. Ang, Y. Y. Thoo, L. F. Siow, *Food Bioproc Tech* **2023**, 1.

[7] J. Sheng, Z. Zu, Y. Zhang, H. Zhu, J. Qi, T. Zheng, Y. Tian, L. Zhang, *J Mater Chem B* **2022**, *10* (31), 5925.

[8] M. Masaki, M. Izumi, Y. Oshima, Y. Nakaoka, T. Kuroda, R. Kimura, S. Sugiyama, K. Terai, M. Kitakaze, K. Yamauchi-Takihara, I. Kawase, H. Hirota, *Circulation* **2005**, *111* (21), 2752.

[9] a) J. Lin, J. Ren, D. S. Gao, Y. Dai, L. Yu, *Front Chem* **2021**, *9*, 669308; b) S. T. Ni, Q. Li, Y. Chen, F. L. Shi, T. S. Wong, L. S. Yuan, R. Xu, Y. Q. Gan, N. Lu, Y. P. Li, Z. Y. Zhou, L. H. Xu, X. H. He, B. Hu, D. Y. Ouyang, *Inflammation* **2024**, *47* (1), 285.

[10] Q. Xie, S. Li, X. Feng, J. Shi, Y. Li, G. Yuan, C. Yang, Y. Shen, L. Kong, Z. Zhang, *J Nanobiotechnology* **2022**, *20* (1), 226.

[11] S. P. Kwon, B. H. Hwang, E. H. Park, H. Y. Kim, J. R. Lee, M. Kang, S. Y. Song, M. Jung, H. S. Sohn, E. Kim, C. W. Kim, K. Y. Lee, G. C. Oh, E. Choo, S. Lim, Y. Chung, K. Chang, B. S. Kim, *Small* **2021**, *17* (32), e2101207.
